# Supplementary material for: Health Literacy in Africa—A Scoping Review of Scientific Publications
Source: Int J Environ Res Public Health. 2024 Oct 31;21(11):1456. doi: 10.3390/ijerph21111456 (PMC11594271; doi:10.3390/ijerph21111456)
Supplement: Supplementary file 1 [file ijerph-21-01456-s001.zip › AfricanHL_Supplement file S1.pdf]

Supplement File S1: Health literacy in Africa

Search strategy:

("health literacy"[Title/Abstract] OR "littératie en santé"[Title/Abstract] OR "compétences en matière de santé"[Title/Abstract])

AND

(Afric*[Title/Abstract] OR Afriq*[Title/Abstract] OR Algeria*[Title/Abstract] OR Angola*[Title/Abstract] OR Benin*[Title/Abstract] OR Botswana*[Title/Abstract] OR Burkin*[Title/Abstract] OR “Burkina Faso” [Title/Abstract] OR Burundi*[Title/Abstract] OR “Cape Verde*”[Title/Abstract] OR “Cabo Verde*”[Title/Abstract] OR Verde*[Title/Abstract] OR Cameroon*[Title/Abstract] OR Chad*[Title/Abstract] OR “Central African*”[Title/Abstract] or “Central African Republic” [Title/Abstract] OR Comor*[Title/Abstract] OR Congo*[Title/Abstract] OR DRC[Title/Abstract] OR “Democratic Republic Congo”[Title/Abstract] OR Djibouti*[Title/Abstract] OR Egypt*[Title/Abstract] OR Equato*[Title/Abstract] OR Eritrea*[Title/Abstract] OR Eswatini[Title/Abstract] OR Swazi*[Title/Abstract] OR Ethiopia*[Title/Abstract] OR Gabon*[Title/Abstract] OR Gambia*[Title/Abstract] OR Ghana*[Title/Abstract] OR Guinea*[Title/Abstract] OR Guinea-Bissau[Title/Abstract] OR Guinea-Bissau*[Title/Abstract] or Bissau-Guinean*[Title/Abstract] OR "Ivory Coast"[Title/Abstract] OR Ivorian*[Title/Abstract] OR Côte d’Ivoire[Title/Abstract] OR Kenya*[Title/Abstract] OR Lesotho[Title/Abstract] OR Basotho*[Title/Abstract] OR Liberia*[Title/Abstract] OR Libya*[Title/Abstract] OR Madagasca*[Title/Abstract] OR Malagasy*[Title/Abstract] OR Malawi*[Title/Abstract] OR Mali[Title/Abstract] OR Malian*[Title/Abstract] OR Mauritania*[Title/Abstract] OR Mauriti*[Title/Abstract] OR Morocc*[Title/Abstract] OR Mozambi*[Title/Abstract] OR Namibia*[Title/Abstract] OR Niger*[Title/Abstract] OR Nigeria*[Title/Abstract] OR Rwanda*[Title/Abstract] OR "São Tomé"[Title/Abstract] OR "Sao Tom*"[Title/Abstract] OR Senegal*[Title/Abstract] OR Seychell*[Title/Abstract] OR Sierra Leon*[Title/Abstract] OR Somali*[Title/Abstract] OR "South Africa*"[Title/Abstract] OR "South Sudan*"[Title/Abstract] OR Sudan*[Title/Abstract] OR Tanzania*[Title/Abstract] OR Togo*[Title/Abstract] OR Tunisia*[Title/Abstract] OR Uganda*[Title/Abstract] OR Zambia*[Title/Abstract] OR Zimbabwe*[Title/Abstract])

NOT

("African American*"[Title/Abstract])
