# Supplementary material for: Health Literacy in Africa—A Scoping Review of Scientific Publications
Source: Int J Environ Res Public Health. 2024 Oct 31;21(11):1456. doi: 10.3390/ijerph21111456 (PMC11594271; doi:10.3390/ijerph21111456)
Supplement: Supplementary file 1 [file ijerph-21-01456-s001.zip › AfricanHL_supplement file S2.pdf]

Supplement File S2: HL in Africa – overview of included articles

Author	Country	Year of Pub.	Title	Study aim
Azmat et al.[44]	Africa and Latin America, not country specific	2021	Scoping review on the impact of outbreaks on sexual and reproductive health services: Proposed frameworks for pre-, intra-, and postoutbreak situations.	Yet, to date, there is no review studying the possible impact of outbreaks on sexual and reproductive health
Ganasen et al.[45]	Africa multiple countries not specific	2008	Mental health literacy: focus on developing countries.	This is a non-systematic review of published articles on mental health literacy in the general population and among primary healthcare workers, in particular, in developing countries
Gresh et al.[46]	Africa not country specific	2021	Postpartum care content and delivery throughout the African continent: An integrative review.	The objective of this review was to describe and evaluate the content of postpartum care and models of delivery throughout the African continent.
Ogunbodede et al.[47]	Africa, no country specific	2015	Oral health inequalities between rural and urban populations of the African and Middle East Region.	The purpose of this review is to highlight some of the conceptual issues relating to urban-rural inequalities in oral health, especially in the African and Middle East Region (AMER) of the International Association for Dental Research as defined in 2003
Park[48]	Africa, no country specified	2021	The effect of advances in transportation on the spread of the coronavirus disease: The last is Africa and endemic.	The objective of this review was to evaluate the historical events related to advances in transportation and the spread of infectious diseases to help address these questions, and to rationally assess and present the progress on COVID-19, transportation, and population movement to date.
Afolabi et al.[49]	Africa, not specified	2021	What constitutes a palliative care need in people with serious illnesses across Africa? A mixed-methods systematic review of the concept and evidence.	To synthesise primary evidence from Africa for palliative care needs among patients and families with serious illness.
Chidzonga et al.[50]	Africa, not specified	2015	Determinants of oral diseases in the African and Middle East Region.	The aim of this report is to review literature on determinants of oral diseases and apply the concepts to promoting oral health in the African countries in the African and Middle East region (AMER).
Lucero-Prisno et al.[51]	Africa, not specified	2020	Current efforts and challenges facing responses to 2019-nCoV in Africa.	This article aims at providing a critical commentary on the current efforts against 2019-nCoV pandemic and the challenges facing its responses in the African continent.
Vearey et al.[52]	Africa, not specified	2019	Urban health in Africa: a critical global public health priority.	This paper highlights the importance of action in the African continent for achieving global public health targets. Specifically, we argue that a focus on urban health in Africa is urgently required in order to support progress on the Sustainable Development Goals (SDGs) and other global and regional public health targets, including Universal Health Coverage (UHC), the new Urban Agenda, and the African Union's Agenda 2063.
Ouedraogo et al.[53]	Africa, overall, not country specific (review)	2022	A systematic review on improving health literacy in rural Africa using mobile serious games.	This paper reviews mobile serious games in healthcare education, especially those intended to improve health literacy in rural Africa.
Ezenwankwo et al.[54]	Africa, overall	2022	Behavioural oncology research in Africa: Lessons from the last two decades and key considerations moving forward.	To analyse the state of behavioural oncology research in Africa and outline key considerations for future research.
Okereke[55]	Africa_rural Africa	2021	COVID-19 misinformation and infodemic in rural Africa.	There is an urgent need to investigate the overall impact of the COVID-19 pandemic in Africa as well as provide novel insights on access to information on COVID-19 in rural Africa.

Kickbusch[56]	Africa, worldwide	2001	Health literacy: addressing the health and education divide.	Review of concepts and definitions of literacy and health literacy
Acheampong et al.[57]	Africa: 43 countries	2019	Priority setting towards achieving under-five mortality target in Africa in context of sustainable development goals: an ordinary least squares (OLS) analysis.	The primary objective of our study was to examine the socioeconomic, healthcare, and environmental determinants that most account for U5MR disparities between African countries.
Petersen et al.[58]	Africa: Ethiopia, Nigeria, South Africa, Uganda	2017	Strengthening mental health system governance in six low- and middle-income countries in Africa and South Asia: challenges, needs and potential strategies.	The aim of this study was to identify key governance challenges, needs and potential strategies that could facilitate adequate integration of mental health into primary health care settings in low- and middle-income countries.
Basu et al.[59]	African countries	2014	Patient guardians as an instrument for person centered care.	not explicitly specified
Ramos et al.[60]	Angola	2022	Building a health literacy indicator from Angola Demographic and Health Survey in 2015/2016.	This study aimed to develop a health literacy indicator for Angola and to analyze pertinent demographic characteristics related to it.
Inegbenosun and Azodo[61]	Benin	2020	Association between oral health literacy, gingival health and oral hygiene among dental patients.	To determine the association between oral health literacy, oral hygiene and gingival health status.
Tsekane and Amone-P'olak[62]	Botswana	2019	Self-efficacy as a predictor of alcohol use among students at a University in Botswana.	This study assessed the influence of self-efficacy on alcohol use among students at a university in Botswana.
Banke-Thomas et al.[63]	Burkina Faso	2013	Knowledge of obstetric fistula prevention amongst young women in urban and rural Burkina Faso: a cross-sectional study.	This study aims to evaluate knowledge on obstetric fistula among young women in a health district of Burkina Faso, comparing rural and urban communities.
Diendéré et al.[64]	Burkina Faso	2022	Oral hygiene practices and their sociodemographic correlates among adults in Burkina Faso: results from the First National Survey.	This study aims to describe oral hygiene practices and associated sociodemographic factors in the Burkinabè population using the first nationally representative data.
Some et al.[65]	Burkina Faso	2022	Interactive voice response service to improve high school students Covid-19 literacy in Burkina Faso: a usability study.	In this study, we present a Mobile based Interactive Voice Response service designed particularly for low-literate people which provides validated Covid-19 related health information in local African languages. We conducted a field study, among high school students, through a usability study to assess users' perception.
Moukam et al.[66]	Cameroon	2021	"Cervical cancer screening: awareness is not enough". Understanding barriers to screening among women in West Cameroon-a qualitative study using focus groups.	Our first and main objective was to understand the barriers affecting women's decision-making process regarding participation in a cervical cancer screening program in the Dschang district (West Cameroon). Second, we aimed to explore the acceptability and perception of a single-visit approach (screen and treat).
Costa et al. [67]	Cape Verde	2021	I-DECIDE: A social prescribing and digital intervention protocol to promote sexual and reproductive health and quality of life among young Cape Verdeans.	In this paper, we present a protocol of a community-based social prescribing and digital intervention to promote wellbeing and quality of life across the life course of young Cape Verdeans, with a specific focus on Sexual and Reproductive Health (SRH) related behaviors.
Mensah et al.[68]	Côte d'Ivoire	2020	Acceptability of HPV screening among HIV-infected women attending an HIV-dedicated clinic in Abidjan, Côte d’Ivoire.	Our study aims to assess the preintervention acceptability of HPV screening among HIV-infected women in Abidjan, Côte d’Ivoire.

Malamba-Lez et al.[69]	Democratic Republic of Congo	2018	Heart failure etiologies and challenges to care in the developing world: an observational study in the Democratic Republic of Congo.	Limited data are available regarding causes and outcomes of heart failure as well as organization of care in the developing world.
Stroeken et al.[70]	Eastern and Southern Africa	2011	HIV among out-of-school youth in Eastern and Southern Africa: a review.	a systematic review of research relating out-of-school youth and HIV seems timely
Almaleh et al.[71]	Egypt	2017	Assessment of health literacy among outpatient clinics attendees at Ain Shams University Hospitals, Egypt: a cross-sectional study.	The aim of conducting this study is to measure health literacy (HL) and to investigate the factors associated with inadequate HL in a sample of outpatient clinics attendees (i.e. patients and companions) at Ain Shams University (ASU) Hospitals.
Alseraty[72]	Egypt	2015	Parents' Socioeconomic Status and Health Literacy Domains among Shokrof Preparatory School Students, Shokrof Village, Algarbia Governorate, Egypt.	This study aimed to assess the correlation between parents' socioeconomic status and health literacy domains among Shokrof preparatory school students
Anwar et al.[73]	Egypt	2020	Health literacy strengths and limitations among rural fishing communities in Egypt using the Health Literacy Questionnaire (HLQ).	The aim of this study was to use a multi-dimensional measurement tool to describe the health literacy of people living in a fishing community in northern Egypt.
Anwar et al.[74]	Egypt	2021	Health Literacy Co-Design in a Low Resource Setting: Harnessing Local Wisdom to Inform Interventions across Fishing Villages in Egypt to Improve Health and Equity.	This study aimed to implement a co-design process as a step towards developing health literacy interventions to improve health and equity in the Borollos Lake region of northern Egypt, a low resource setting with a high prevalence of chronic diseases.
McEwan et al.[75]	Egypt	2019	"Injustice! That is the cause": a qualitative study of the social, economic, and structural determinants of late diagnosis and treatment of breast cancer in Egypt.	We conducted a qualitative study to deepen our understanding of women's experiences with diagnosis and treatment delays and highlight nuances not identifiable in the quantitative studies.
Mostafa et al.[76]	Egypt	2021	Is health literacy associated with antibiotic use, knowledge and awareness of antimicrobial resistance among non-medical university students in Egypt? A cross-sectional study.	This study examined the levels of health literacy (HL) and their association with antibiotic use, knowledge of antibiotics and awareness of antibiotic resistance among university students in Egypt.
Wahba et al.[77]	Egypt	2017	Consumer-oriented evaluation of the service provided by the department of health education and information in Alexandria, Egypt.	A simple random sample of 400 participants was interviewed using an interview questionnaire assessing respondents' socioeconomic characteristics, health literacy, attitude toward health education services, and satisfaction with the health education service provided by the DHEI.
Ngwenya et al.[78]	Eswatini	2022	Urban-Rural Differences in Depression Literacy Among High School Teachers in the Kingdom of Eswatini.	This study aims to investigate adolescent depression literacy among teachers in Eswatini and to explore the role of urbanicity
Asamrew et al.[79]	Ethiopia	2020	Level of Patient Satisfaction with Inpatient Services and Its Determinants: A Study of a Specialized Hospital in Ethiopia.	This study was performed with the aim of assessing the level of patient satisfaction with inpatient services and its determinants in Black Lion Specialized Hospital, Addis Ababa, Ethiopia.
Asemahagn[80]	Ethiopia	2021	Sputum smear conversion and associated factors among smear-positive pulmonary tuberculosis patients in East Gojjam Zone, Northwest Ethiopia: a longitudinal study.	This study aimed at estimating sputum smear conversion and identifying factors hindering sputum smear conversion among bacteriologically confirmed PTB cases in East Gojjam Zone, Northwest Ethiopia.
Asemahagn et al.[81]	Ethiopia	2020	A Qualitative Insight into Barriers to Tuberculosis Case Detection in East Gojjam Zone, Ethiopia.	The objective of this study was to identify possible barriers to TCD in East Gojjam Zone, northwest Ethiopia.

Avan et al.[82]	Ethiopia	2021	Embedding Community-Based Newborn Care in the Ethiopian health system: lessons from a 4-year programme evaluation.	Using the CHW functionality model by WHO, this study evaluates the health system response to the programme, including quality of care.
Ayele et al.[83]	Ethiopia	2019	Magnitude and determinants for place of postnatal care utilization among mothers who delivered at home in Ethiopia: a multinomial analysis from the 2016 Ethiopian demographic health survey.	Therefore, this study assessed the magnitude and determinants for place of postnatal care service utilization among mothers who delivered at home in Ethiopia.
Ayode et al.[84]	Ethiopia	2012	The association of beliefs about heredity with preventive and interpersonal behaviors in communities affected by podoconiosis in rural Ethiopia.	Little is known about how beliefs about heredity as a cause of health conditions might influence preventive and interpersonal behaviors among those individuals with low genetic and health literacy.
Chereka et al.[85]	Ethiopia	2022	Digital health literacy to share COVID-19 related information and associated factors among healthcare providers worked at COVID-19 treatment centers in Amhara region, Ethiopia: A cross-sectional survey.	Therefore, this study aimed to assess digital health literacy to share COVID-19 related information and associated factors among healthcare providers who worked at COVID-19 treatment centers in the Amhara region, Northwest Ethiopia.
Dugasa[86]	Ethiopia	2022	Level of Patient Health Literacy and Associated Factors Among Adult Admitted Patients at Public Hospitals of West Shoa Oromia, Ethiopia.	The aim of this study was to assess level of patient health literacy and associated factors among adult admitted patients at public hospitals of West Shoa zone, Oromia, Ethiopia.
Gedefaw et al.[87]	Ethiopia	2020	Information Seeking Behavior About Cancer and Associated Factors Among University Students, Ethiopia: A Cross-Sectional Study.	This study aimed to assess cancer information seeking behavior (CISB) and its associated factors among students in Debre Tabor University, Ethiopia.
Gonete et al.[88]	Ethiopia	2021	Malnutrition and contributing factors among newborns delivered at the University of Gondar Hospital, Northwest Ethiopia: a cross-sectional study.	To estimate the prevalence of various indicators of malnutrition (stunting, wasting, low birth weight, concurrent stunting and wasting, overweight/obesity and double burden malnutrition) among newborns and to investigate factors associated with these nutritional disorders.
Gurmu et al.[89]	Ethiopia	2018	Factors associated with self-care practice among adult diabetes patients in West Shoa Zone, Oromia Regional State, Ethiopia.	The aim of this study was to assess factors associated with self-care practice among adult diabetes patients in public hospitals of West Shoa Zone, Oromia Regional State, Ethiopia.
Kassahun et al.[90]	Ethiopia	2016	Diabetes related knowledge, self-care behaviours and adherence to medications among diabetic patients in Southwest Ethiopia: a cross-sectional survey.	We assessed levels of knowledge about type 2 diabetes mellitus (T2DM), self-care behaviours and adherence to medication among DM patients.
Kassie et al.[91]	Ethiopia	2022	Information seeking about COVID-19 and associated factors among chronic patients in Bahir Dar city public hospitals, Northwest Ethiopia: a cross-sectional study.	Therefore, this study aimed to assess information seeking about COVID-19 and associated factors among chronic patients.
Kebede and Wabe[92]	Ethiopia	2012	Medication adherence and its determinants among patients on concomitant tuberculosis and antiretroviral therapy in South west Ethiopia.	The study was designed to assess the degree of drug adherence and its determinants in patients living with HIV/AIDS and TB comorbidity.
Menberu et al.[93]	Ethiopia	2018	Health care seeking behavior for depression in Northeast Ethiopia: depression is not considered as illness by more than half of the participants.	The aim of this study was to assess the level of professional help-seeking behavior and associated factors among individuals with depression.

Mengiste et al.[94]	Ethiopia	2021	Information-seeking behavior and its associated factors among patients with diabetes in a resource-limited country: a cross-sectional study	This study aims to assess the diabetes information-seeking behavior and its associated factors among patients with diabetes in Debre Markos Referral Hospital, Amhara Region, Northwest Ethiopia.
Posso et al.[95]	Ethiopia	2021	Community-level health programs and child labor: Evidence from Ethiopia.	Using Ethiopian data, we investigate if exposure to a community-level health program delivered by Health Extension Workers (HEWs) lowers child labor.
Shahvisi et al.[96]	Ethiopia	2018	A Human Right to Shoes? Establishing Rights and Duties in the Prevention and Treatment of Podoconiosis.	In this paper, we consider the human rights violations that cause, and are caused by, podoconiosis in Ethiopia.
Shiferaw et al.[94]	Ethiopia	2020	E-health literacy and associated factors among chronic patients in a low-income country: a cross-sectional survey.	The aim of this study was to assess eHealth literacy level and associated factors among internet user chronic patients in North-west Ethiopia.
Tefera et al.[97]	Ethiopia	2020	Diabetic health literacy and its association with glycemic control among adult patients with type 2 diabetes mellitus attending the outpatient clinic of a university hospital in Ethiopia.	This study was aimed to assess the diabetic health literacy level and its association with glycemic control among adult patients with type 2 diabetes mellitus attending the outpatient clinic of University of Gondar Comprehensive Specialized Hospital (UOGCSH): Northwest Ethiopia.
Teklu et al.[98]	Ethiopia	2022	Awareness of diagnosis, treatment plan and prognosis among patients attending public hospitals and health centers in Addis Ababa, Ethiopia.	The aim of this study was to examine awareness of diagnosis, treatment plan and prognosis among patients at the time of their exit from public hospitals and health centers.
Tesfaye et al.[99]	Ethiopia	2021	Knowledge of the community regarding mental health problems: a cross-sectional study.	This study aimed to assess the knowledge regarding mental health problems and associated factors among communities of Jimma Zone, Oromia, Ethiopia.
Tilahun et al.[100]	Ethiopia	2021	Communicative health literacy in patients with non-communicable diseases in Ethiopia: a cross-sectional study.	Therefore, this study aimed to assess communicative health literacy and associated factors in patients with NCDs on follow-up at Jimma Medical Center (JMC), Ethiopia.
Tilahun et al.[101]	Ethiopia	2021	Functional Health Literacy in Patients with Cardiovascular Diseases: Cross-Sectional Study in Ethiopia.	This study assessed functional health literacy and associated factors among adult patients with cardiovascular diseases (CVDs) in Ethiopia.
Tora et al.[102]	Ethiopia	2017	Health beliefs of school-age rural children in podoconiosis-affected families: A qualitative study in Southern Ethiopia.	This study therefore aimed to explore the health beliefs of school-age rural children in podoconiosis-affected families.
Adu et al.[103]	Ghana	2021	Mental health literacy in Ghana: Implications for religiosity, education and stigmatization.	This vignette study was conducted to explore the relationships between religiosity, education, stigmatization and MHL among Ghanaians using a sample of laypeople (N = 409)
Amoah[104]	Ghana	2018	Social participation, health literacy, and health and well-being: A cross-sectional study in Ghana.	In particular, existing studies have not explored the relationship between social participation and health literacy and how it affects health and well-being adequately. This paper addresses this gap
Amoah[105]	Ghana	2019	The Relationship among Functional Health Literacy, Self-Rated Health, and Social Support among Younger and Older Adults in Ghana.	It examines the impact each type of support makes on the relation between functional health literacy (FHL) and self-rated health status among younger and older adults in Ghana
Amoah and Phillips[106]	Ghana	2018	Health literacy and health: rethinking the strategies for universal health coverage in Ghana.	However, the role of HL in the relationships between elements of UHC such as access to health care and health insurance has not been widely explored.
Amoah and Phillips[107]	Ghana	2020	Socio-demographic and behavioral correlates of health literacy: a gender perspective in Ghana.	The present analyses examined the role of socio-economic, health and behavioral factors associated with HL among men and women in the Ashanti Region of Ghana

Amoah et al.[108]	Ghana	2022	Health Behaviors and Health Literacy: Questing the Role of Weak Social Ties Among Older Persons in Rural and Urban Ghana.	This study investigated the moderating role of weak social ties (bridging social capital) in the relationship between health behaviors, such as smoking, alcohol intake, voluntary body check-up and physical exercise, and health literacy among older persons in rural and urban Ghana.
Amoah et al.[109]	Ghana	2022	Association of Health Literacy and Socioeconomic Status with Oral Health Among Older Adults in Ghana: A Moderation Analysis of Social Capital.	We examined the moderating role of social capital (SC) in the association of socioeconomic status (SES) and health literacy (HL) with oral health (OH) status and the intentions to use OH services (IUOHS) among older Ghanaians.
Arthur et al.[110]	Ghana	2020	Effectiveness of a Problem-Solving, Story-Bridge Mental Health Literacy Programme in Improving Ghanaian Community Leaders' Attitudes towards People with Mental Illness: A Cluster Randomised Controlled Trial.	The aim of the study was to assess the effectiveness of a mental health literacy programme in improving community leaders' attitudes toward people with mental disorders.
Arthur et al.[111]	Ghana	2020	Cluster randomised controlled trial of a problem-solving, Story-bridge mental health literacy programme for improving Ghanaian community leaders' knowledge of depression.	The aim of this study was to assess the effectiveness of a problem-solving, Story-bridge mental health literacy programme, in improving community leaders' knowledge about helpful interventions for, and recognition of, depression.
Arthur et al.[112]	Ghana	2021	Qualitative process evaluation of a problem-solving and Story-bridge based mental health literacy program with community leaders in Ghana.	The aims of our study were to evaluate the perspectives of community leaders about the usefulness of a cluster randomized trial of a problem-solving and Story-bridge based mental health literacy (MHL) programme and to understand whether they utilized the knowledge acquired from the programme in their usual interactions with people with mental illness and their families
Arthur et al.[113]	Ghana	2018	Evaluation of a mental health literacy program on community leaders’ knowledge about and attitudes towards people with mental disorder in the Brong-Ahafo region in Ghana: Cluster randomised controlled trial.	The broad aim of this study was to evaluate the effectiveness of a mental health literacy programme on Ghanaian assembly members’ knowledge about and attitudes toward people with mental disorders.
Boateng et al.[114]	Ghana	2020	Translation, cultural adaptation and psychometric properties of the Ghanaian language (Akan; Asante Twi) version of the Health Literacy Questionnaire.	The HLQ has been translated and validated in diverse contexts but has so far not been assessed in any country in sub-Saharan Africa. We sought to translate this tool into the most common language used in Ghana and assess its validity.
Boateng et al.[115]	Ghana	2021	Co-creation and prototyping of an intervention focusing on health literacy in management of malaria at community-level in Ghana.	This study used a local needs driven approach to develop a health literacy intervention for caregivers in Ghana concerning management of malaria in children under 5 years.
Evans et al.[116]	Ghana	2019	Comprehensive Health Literacy Among Undergraduates: A Ghanaian University-Based Cross-Sectional Study.	This study aimed to ascertain the levels of health literacy and its sociodemographic determinants among undergraduate university students of Kwame Nkrumah University of Science and Technology.
Gupta et al.[117]	Ghana	2018	Self-reported functional, communicative, and critical health literacy on foodborne diseases in Accra, Ghana.	This study, therefore, examines health literacy on foodborne diseases and the relative effects of health literacy on self-rated health.
Koduah et al.[118]	Ghana	2019	"I Sometimes Ask Patients to Consider Spiritual Care": Health Literacy and Culture in Mental Health Nursing Practice.	This paper examines how local precepts, within culture and language, shape mental health nurses' (MHNs) practice and understanding of patients' health literacy level in Ghana.
Koduah et al.[119]	Ghana	2021	A Comparative Analysis of Student and Practising Nurses' Health Literacy Knowledge in Ghana.	This study examined student and practising nurses' health literacy knowledge, and its correlates in Ghana
Kpobi and Swartz[120]	Ghana	2018	Explanatory models of mental disorders among traditional and faith healers in Ghana.	With the concept of explanatory models of illness as a framework, we examined the notions of different categories of traditional and faith healers about mental disorders.

Kugbey et al.[121]	Ghana	2019	Access to health information, health literacy and health-related quality of life among women living with breast cancer: Depression and anxiety as mediators.	This study examined the direct and indirect influences of health literacy and access to health information on the quality of life among 205 women living with breast cancer in Ghana.
Lori et al.[122]	Ghana	2016	Use of a facilitated discussion model for antenatal care to improve communication.	To examine the usefulness and feasibility of providing focused antenatal care (FANC) in a group setting using picture cards to improve patient-provider communication, patient engagement, and improve health literacy.
Lori et al.[123]	Ghana	2017	Improving health literacy through group antenatal care: a prospective cohort study.	To examine whether exposure to group antenatal care increased women's health literacy by improving their ability to interpret and utilize health messages compared to women who received standard, individual antenatal care in Ghana.
Lori et al.[124]	Ghana	2014	Examining antenatal health literacy in Ghana.	To explore Ghanaian pregnant women's understanding and recognition of danger signs in pregnancy, birth preparedness and complication readiness, and their understanding of newborn care.
Nangsangna and Vroom[125]	Ghana	2019	Factors influencing online health information seeking behaviour among patients in Kwahu West Municipal, Nkawkaw, Ghana.	This cross sectional study was conducted in the Kwahu West Municipal to determine factors influencing online health information seeking behaviours among patients.
Seneadza et al.[126]	Ghana	2022	Neonatal jaundice in Ghanaian children: Assessing maternal knowledge, attitude, and perceptions.	As part of a project to evaluate a screening tool for NNJ, we assessed the knowledge, attitude, and perceptions of Ghanaian mothers on NNJ at baseline.
Tutu et al.[127]	Ghana	2019	Exploring the development of a household cholera-focused health literacy scale in James Town, Accra.	This study attempts to develop and pilot a tool to measure household health literacy among the urban poor in James Town, a cholera endemic neighborhood.
Tutu et al.[128]	Ghana	2019	Examining health literacy on cholera in an endemic community in Accra, Ghana: a cross-sectional study.	This study, therefore, assesses health literacy on cholera and the association between health literacy competency and health outcome.
Darteh et al.[129]	Ghana/Kenya	2020	Understanding the Socio-demographic Factors Surrounding Young Peoples' Risky Sexual Behaviour in Ghana and Kenya.	The study examined risky sexual behaviours among the youth in Ghana and Kenya in relation to socio-demographic characteristics. T
McGinn and Allen[130]	Guinea	2006	Improving refugees' reproductive health thorough literacy in Guinea.	Adult literacy programmes, particularly literacy-for-health programmes that integrate health material in their curricula, are gaining momentum as a means to improve women's and children's health and increase women's empowerment. However, the relationship between literacy skills and these benefits remains unclear.
Dyrehave et al.[131]	Guinea-Bissau	2016	Nonadherence is Associated with Lack of HIV-Related Knowledge: A Cross-Sectional Study among HIV-Infected Individuals in Guinea-Bissau.	assessment of adherence and HIV-related knowledge.
Abajobir et al.[132]	Kenya	2021	The impact of i-PUSH on maternal and child health care utilization, health outcomes, and financial protection: study protocol for a cluster randomized controlled trial based on financial and health diaries data.	This study aims to evaluate the impact of i-PUSH on maternal and child health care utilization, women's health including their knowledge, behavior, and uptake of respective services, as well as women's empowerment and financial protection. It also aims to evaluate the impact of the LEAP training tool on empowering and enhancing community health volunteers' health literacy and to evaluate the impact of the M-TIBA health wallet on savings for health and health insurance uptake.
Kassaman et al.[133]	Kenya	2022	Fear, faith and finances: health literacy experiences of English and Swahili speaking women newly diagnosed with breast and cervical cancer.	This exploratory qualitative study investigated the HL experiences of accessing and using health information in women with any stage of breast or cervical cancer presenting at the Aga Khan University Hospital (private) or Kenyatta National Hospital (public) in Nairobi, Kenya.
Khares et al.[134]	Kenya	2018	Quality of care provided to adolescents aged between 10 and 19 years in Kenyatta National Hospital, Nairobi, Kenya	This study explored the nature of Adolescent Health Care, in both the inpatient and outpatient settings at Kenyatta National Hospital, the largest referral hospital in Kenya.
Marangu et al.[135]	Kenya	2021	Assessing mental health literacy of primary health care workers in Kenya: a cross-sectional survey.	To assess mental health literacy of health workers in primary health care services in Kenya.

McMahon et al.[136]	Kenya	2022	Barriers and facilitators to chemotherapy initiation and adherence for patients with HIV-associated Kaposi's sarcoma in Kenya: a qualitative study.	The objective of this qualitative study conducted with people living with HIV-associated KS in Kenya was to identify and understand barriers and facilitators to chemotherapy initiation and adherence.
Muga et al.[137]	Kenya	2018	A Pilot Study Exploring Nursing Knowledge of Depression and Suicidal Ideation in Kenya.	This cross-sectional study assessed mental health literacy (depression and suicidal ideation) among nurses in a private urban referral hospital in Kenya
Mutiso et al.[138]	Kenya	2018	Changing patterns of mental health knowledge in rural Kenya after intervention using the WHO mhGAP-Intervention Guide.	This study sought to determine the feasibility of using the World Health Organization mhGAP-Intervention Guide (IG) as an educational tool for one-on-one contact in a clinical setting to increase literacy on the specified mental disorders.
Mwaisaka et al.[139]	Kenya	2021	Young People's Experiences Using an On-Demand Mobile Health Sexual and Reproductive Health Text Message Intervention in Kenya: Qualitative Study.	The objective of this study was to explore young people's experiences using an on-demand SRH mHealth platform in Kenya.
Mwiti et al.[140]	Kenya	2019	'*Bottom up*' approach: A community-based intervention in fighting non-communicable diseases in urban informal settlements Kenya	Objective: The main objective of this study was to identify challenges faced by healthcare front-liners towards health promotion in the prevention and management of Non-Communicable Diseases (NCDs) amongst the urban 25 - 59-year olds living in urban informal settlements within Kamukunji, Nairobi County.
Raufman et al.[141]	Kenya	2020	Environmental health literacy and household air pollution-associated symptoms in Kenya: a cross-sectional study.	Our objective was to evaluate the association between environmental health literacy (EHL), a domain of health literacy (HL) that describes the ability to use environmental health information to reduce health risks, and symptoms associated with HAP.
Sripad et al.[142]	Kenya	2022	Determining a Trusting Environment for Maternity Care: A Framework Based on Perspectives of Women, Communities, Service Providers, and Managers in Peri-Urban Kenya.	We used a theoretically driven qualitative approach to explore trust determinants in a maternity setting across patient-provider, inter-provider, and community-policymaker interactions and relationships in peri-urban Kenya.
Mugomeri et al.[143]	Lesotho	2016	Knowledge of disease condition and medications among hypertension patients in Lesotho.	This study evaluated the levels of knowledge of hypertension and the associated medications among hypertension patients in Lesotho and assessed the significance of these indicators on hypertension treatment outcomes
Reid et al.[144]	Lesotho	2019	Development of a Sesotho health literacy test in a South African context.	The researchers aimed to develop an appropriate HL test for use among South African public health service users with Sesotho as their first language.
Brick et al.[145]	Liberia	2021	Training-of-Trainers Neuroscience and Mental Health Teacher Education in Liberia Improves Self-Reported Support for Students.	The aim of this study was to determine if a program combining an understanding of neuroscience with mental health literacy content could increase teachers' awareness of students' mental health issues and produce changes in teacher attitudes and classroom practices.
Asgary et al.[146]	Madagascar	2015	Malnutrition prevalence and nutrition barriers in children under 5 years: a mixed methods study in Madagascar.	We aimed to assess the point prevalence of childhood malnutrition in the Anivorano region, and to explore mothers' perceptions of barriers to proper nutrition
Harimbola and Mizumoto[147]	Madagascar	2018	Individual and Household Risk Factors for Severe Acute Malnutrition among Under-Five Children in the Analamanga Region, Madagascar.	This study explores the risk factors for SAM among children hospitalized with SAM in the Analamanga region of Madagascar.
Jumbe et al.[148]	Malawi	2022	We do not talk about it': Engaging youth in Malawi to inform adaptation of a mental health literacy intervention.	We share our experience of engaging youth in Malawi through advocacy organisations to inform cultural adaptation of a mental health literacy intervention.
Kalanda et al.[149]	Malawi	2005	Catch-up growth in Malawian babies, a longitudinal study of normal and low birthweight babies born in a malarious endemic area.	To describe growth patterns in infants with low and normal birthweight and determine maternal risk factors for infant undernutrition.

Kohler et al.[150]	Malawi	2020	Cohort profile: the mature adults cohort of the Malawi longitudinal study of families and health (MLSFH-MAC).	The Mature Adults Cohort of the Malawi Longitudinal Study of Families and Health (MLSFH-MAC) contributes to global ageing studies by providing a rare opportunity to study the processes of individual and population ageing, the public health and social challenges associated with ageing and the coincident shifts in disease burdens, in a low-income, high HIV prevalence, sub-Saharan African (SSA) context.
Kululanga et al.[151]	Malawi	2020	Knowledge deficit on health promotion activities during pregnancy: the case for adolescent pregnant women at Chiladzulu District, Malawi.	Therefore, the aim of the study was to explore knowledge of pregnant adolescents on importance of antenatal care and health promotion during pregnancy.
Kutcher et al.[152]	Malawi	2017	Clinic outcomes of the Pathway to Care Model: A cross-sectional survey of adolescent depression in Malawi.	To address this need, a Canadian-developed youth depression Pathway to Care Model, linking school-based mental health literacy interventions to training of community healthcare providers, was adapted for use in Malawi and successfully applied.
Kutcher et al.[153]	Malawi	2015	Improving Malawian teachers' mental health knowledge and attitudes: an integrated school mental health literacy approach.	we culturally adapted a previously demonstrated effective Canadian school mental health curriculum resource (the Guide) for use in Malawi, the African Guide: Malawi version (AGMv), and evaluated its impact on enhancing mental health literacy for educators (teachers and youth club leaders)
Ogunrinu et al.[154]	Malawi	2017	A qualitative study of health education experiences and self-management practices among patients with type 2 diabetes at Malamulo Adventist Hospital in Thyolo District, Malawi.	The aim of this study was to understand the perceptions and experiences of health education and self-management practices on Malamulo Adventist Hospital type 2 diabetic patients
Patel et al.[155]	Malawi	2020	Addressing COVID-19 in Malawi.	In this commentary, we provide a quick analysis of the current state of the COVID-19 pandemic in Malawi and its efforts to address it.
Tilly et al.[156]	Malawi	2022	Implementation and Evaluation of Educational Videos to Improve Cancer Knowledge and Patient Empowerment.	Low health literacy is a leading cause of treatment abandonment among patients receiving cancer care at Kamuzu Central Hospital (KCH) in Malawi.
Uwamahoro et al.[157]	Malawi	2019	Health literacy among Malawian HIV-positive youth: a qualitative needs assessment and conceptualization.	We aimed to assess health literacy-related needs of young people living with HIV (YPLHIV) and adapt existing health literacy frameworks to the context of HIV/AIDS in Malawi
Ménard et al.[158]	Morocco	2018	Family caregivers' reported nonadherence to the controller medication of asthma in children in Casablanca (Morocco): Extent and associated factors.	This study aims 1) to assess the extent of children's nonadherence to the controller treatment of asthma in an urban region of Morocco as reported by a family caregiver, and 2) to identify the associated factors.
Park et al.[159]	Morocco	2021	Improving Treatment Adherence with Integrated Patient Management for TB Patients in Morocco.	This study aims to examine the delivery model of TB management and the outcomes of an integrated patient management system that uses a patient-centered and community-based approach, along with mobile health technology.
Howard et al.[160]	Mozambique	2014	Health literacy predicts pediatric dosing accuracy for liquid zidovudine.	We aimed to characterize the frequency of dosing errors for liquid zidovudine using two dosing devices and to evaluate the association between HIV literacy and dosing errors in adults living with HIV infection.
Lindberg et al.[161]	Mozambique	2021	A qualitative study of mothers' health literacy related to malnutrition in under 5-year-old children in southern Mozambique.	To explore mothers' perceptions of malnutrition and its causes in U-5's in Mozambique, as well as their ability to recognise, prevent and act on signs of malnutrition.
Tique et al.[162]	Mozambique	2017	Measuring Health Literacy Among Adults with HIV Infection in Mozambique: Development and Validation of the HIV Literacy Test.	The objective of this study was to develop and rigorously test the psycho-metric properties of a novel measure of health literacy for adults with HIV, the HIV Literacy Test (HIV-LT).
Nair et al.[163]	multi-country approach: Benin	2015	Improving the Quality of Health Care Services for Adolescents, Globally: A Standards-Driven Approach.	The World Health Organization (WHO) undertook an extensive and elaborate process to develop eight Global Standards to improve quality of health care services for adolescents. The objectives of this article are to present the Global Standards and their method of development.

Theron et al.[164]	multiple African countries: South Africa, Zimbabwe, Zambia, Tanzania	2015	Psychological distress and its relationship with non-adherence to TB treatment: a multicentre study.	There are limited data regarding correlates of psychological distress and their association with non-adherence to anti-TB treatment.
Hanlon et al.[165]	multiple countries incl. Ethiopia, South Africa, Uganda	2014	Challenges and opportunities for implementing integrated mental health care: a district level situation analysis from five low- and middle-income countries.	The purpose was to inform development and implementation of a comprehensive district plan to integrate mental health into primary care.
Ramazanu et al.[166]	multiple countries incl. Malawi, Kenya	2022	Challenges and Opportunities in Stroke Nursing Research: Global Views From a Panel of Nurse Researchers.	This article reflects panel insights on challenges and opportunities for nurse-led stroke research
Grady et al.[167]	multiple countries incl. Algeria	2019	Health Care Professionals' Clinical Perspectives and Acceptance of a Blood Glucose Meter and Mobile App Featuring a Dynamic Color Range Indicator and Blood Sugar Mentor: Online Evaluation in Seven Countries.	To gather current self-management perceptions of HCPs in seven countries and investigate HCP satisfaction with a new glucose meter and mobile app featuring a dynamic color range indicator and a blood sugar mentor.
Aung et al.[168]	multiple countries incl. Sudan	2021	Community responses to COVID-19 pandemic first wave containment measures: a multinational study.	This study explored community responses to COVID-19 containment measures in different countries and synthesized a model.
Robertson et al.[169]	multiple countries incl. Uganda	2021	Development and Validity Assessment of a Chronic Obstructive Pulmonary Disease Knowledge Questionnaire in Low- and Middle-Income Countries.	To develop and assess the validity and reliability of a COPD-KQ among individuals with COPD in three LMIC settings.
Pleasant and Kuruvilla[170]	multiple countries: incl. Ghana	2008	A tale of two health literacies: public health and clinical approaches to health literacy.	This article reports on an initial attempt to begin creating a measure based on the public health approach to health literacy. A brief discussion on the putative links between literacy, knowledge and health, from both clinical and public health approaches, provides a useful background for this effort.
Mogobe et al.[171]	multiple countries: Botswana	2016	Language and Culture in Health Literacy for People Living with HIV: Perspectives of Health Care Providers and Professional Care Team Members.	The purpose of this paper was to examine the perspectives of health care providers and professional care team members regarding health literacy in HIV disease
McClintock et al.[172]	multiple countries: Cameroon, Democratic Republic of the Congo, Ethiopia, Ghana, Guinea, Ivory Coast, Lesotho,	2020	Constructing a measure of health literacy in Sub-Saharan African countries.	We sought to develop and evaluate a health literacy measure in a multi-national study and to examine demographic characteristics associated with health literacy.

	Rwanda, Niger, Namibia, Sierra Leone, Swaziland, Togo and Zambia			
Nachega et al.[173]	multiple countries: Côte D'Ivoire, South Africa	2012	HIV treatment adherence, patient health literacy, and health care provider-patient communication: results from the 2010 AIDS Treatment for Life International Survey.	Little is known about patients' health literacy regarding antiretroviral therapy (ART) adherence and drug resistance and patient-provider communication about these topics.
Popoola[174]	multiple countries: Nigeria	2019	Involving libraries in improving health literacy to achieve Sustainable Development Goal-3 in developing economies: a literature review.	The review focused on identifying actions that can be implemented by libraries to enhance health literacy and access to information among health care practitioners and consumers to support the achievement of SDG-3 in developing countries, especially Nigeria.
Weist et al.[175]	multiple countries: Liberia	2017	School Mental Health Promotion and Intervention: Experiences from Four Nations	In this article we provide general background on SMH in four nations, two showing strong progress (the United States and Canada), one showing moderate progress (Norway), and one beginning the work (Liberia). Following general background for each country, actions in relation to the SMHILE themes are reviewed. The article concludes with plans and ideas for future global collaboration towards advancement of the SMH field.
Bowser et al.[176]	multiple countries: MENA: Egypt, North Africa + UAE	2017	Health system barriers and enablers to early access to breast cancer screening, detection, and diagnosis: a global analysis applied to the MENA region.	To identify barriers and enablers that impact access to early screening, detection, and diagnosis of breast cancer both globally and more specifically in the Middle East and North Africa (MENA) region (with a specific focus on Egypt, Jordan, Oman, Saudi Arabia, United Arab Emirates [UAE], and Kuwait) with a specific focus on the health system.
Kutcher et al.[177]	Multiple countries: Malawi, Tanzania	2019	Creating Evidence-Based Youth Mental Health Policy in Sub-Saharan Africa: A Description of the Integrated Approach to Addressing the Issue of Youth Depression in Malawi and Tanzania.	We here describe an innovative approach that addresses these issues simultaneously while concurrently strengthening key mental health components in existing education and health-care systems as successfully applied in Malawi and replicated in Tanzania
Hirvonen et al.[178]	Multiple countries: Namibia	2020	Screening everyday health information literacy among four populations.	This study aims to examine the applicability of a multidimensional Everyday Health Information Literacy (EHIL) screening tool in detecting people with challenges in accessing, understanding, evaluating and using health information in everyday situations.
Pavarini et al.[179]	Multiple countries: Nigeria, South Africa, Burundi	2022	Agents of Change for Mental Health: A Survey of Young People's Aspirations for Participation Across Five Low- and Middle-Income Countries.	To identify pathways for young people's participation in promoting MHWB in low- and middle-income countries (LMICs), this study surveyed young people's aspirations for engagement, their spheres of influence, capacity building needs, and key barriers to participation.
Seytre et al.[180]	Multiple countries: Burkina Faso, Cabo Verde, Guinea-Bissau, Ivory Coast, Sierra Leone	2021	Revisiting COVID-19 Communication in Western Africa: A Health Literacy-based Approach to Health Communication.	We conducted a quantitative socio-anthropological study of the knowledge of the severe acute respiratory syndrome coronavirus 2 (SARS-CoV-2) infection and perception of the prevention messages in Burkina Faso, Cabo Verde, Guinea-Bissau, Ivory Coast, and Sierra Leone.

Al-Rousan et al.[181]	Multiple countries: Cameroon, Malawi	2020	Patients' perceptions of self-management of high blood pressure in three low- and middle-income countries: findings from the BPMONITOR study.	Self-management of high blood pressure (BP) through self-monitoring and self-titration of medications, has proved to be one successful and cost-effective tool to achieve better BP control in many high-income countries but not much is known about its potential in low- and middle-income countries (LMICs).
Louyeh et al.[182]	Multiple countries: Nigeria, Egypt, Malawi, Ghana,	2020	Patterns of the Social Approach to Health in Selected Countries and Iran: A Comparative Study.	This comparative study aimed at examining the patterns of the social approach to health in 9 selected countries.
Velden et al.[183]	Multiple countries: South Africa	2014	Patients with Sore Throat: A Survey of Self-Management and Healthcare-Seeking Behavior in 13 Countries Worldwide.	The objective of this study was to investigate patients' attitudes related to healthcare-seeking behavior and self-management of sore throat.
Austvoll-Dahlgren et al.[184]	Multiple countries: Uganda	2017	Measuring ability to assess claims about treatment effects: a latent trait analysis of items from the 'Claim Evaluation Tools' database using Rasch modelling.	To describe the development of the Claim Evaluation Tools, a set of flexible items to measure people's ability to assess claims about treatment effects.
Korhonen et al.[185]	Multiple countries: South Africa / Zambia	2022	Construct validity and internal consistency of the revised Mental Health Literacy Scale in South African and Zambian contexts.	The aim of this study was to evaluate the construct validity and internal consistency of the revised Mental health literacy Scale (MHLS) in South Africa (SA) and Zambia.
Lahti et al.[186]	Multiple countries: South Africa / Zambia	2020	Design and Development Process of a Youth Depression Screening m-Health Application for Primary Health Care Workers in South Africa and Zambia: An Overview of the MEGA Project.	In phase one, we will investigate the mental health literacy of PHC practitioners to identify areas in need of development.
Kagee et al.[187]	Multiple countries: Southern Africa	2011	Structural barriers to ART adherence in Southern Africa: Challenges and potential ways forward.	not explicitly specified
Paschen-Wolff et al.[188]	Multiple countries Southern Africa: Botswana, Namibia, South Africa, Zimbabwe	2020	HIV and sexually transmitted infection knowledge among women who have sex with women in four Southern African countries.	This study examined the demographic and social factors contributing to female-to-female STI/HIV transmission knowledge among Southern African women who have sex with women using an integrated model of health literacy.
Laisser et al.[189]	Multiple countries: Tanzania / Zambia	2022	The tipping point of antenatal engagement: A qualitative grounded theory in Tanzania and Zambia.	To gain understanding of women's antenatal experiences in Tanzania and Zambia, and the factors that influence antenatal engagement.

Bedrosian et al.[190]	Multiple countries: West Africa	2016	Lessons of Risk Communication and Health Promotion - West Africa and United States.	not explicitly specified
Fowler et al.[191]	Multiple countries: West Africa	2014	Caring for critically ill patients with Ebola virus disease. Perspectives from West Africa.	not explicitly specified
Munangatire et al.[192]	Namibia	2022	Nursing students' understanding of health literacy and health practices: a cross-sectional study at a university in Namibia.	This study explored nursing students' understanding of the concept of ' health literacy' and their health practices at a university in Namibia.
Li et al.[193]	Niger	2016	Using WeChat official accounts to improve malaria health literacy among Chinese expatriates in Niger: an intervention study.	Health education is a recommended intervention for prevention of malaria among non-immune travellers and expatriate workers. It is urgent to develop an effective and feasible way for these populations to obtain information about the prevention and treatment of malaria.
Abaraogu et al.[194]	Nigeria	2016	Work-related back discomfort and associated factors among automotive maintenance mechanics in Eastern Nigeria: A cross sectional study.	To investigate the prevalence, pattern and severity of back pain among automotive maintenance mechanics, as well as the personal and job variables associated with or predicting occurrence of back pain.
Abiodun et al.[195]	Nigeria	2011	Detecting child psychiatric disorders during routine clinic work: A pre-interventional study of primary care physicians in Ilorin, Nigeria	To explore the existing level of ability of PCPs in our primary care unit to identify children with mental health problems.
Adanri et al.[196]	Nigeria	2017	Maternal health literacy, antenatal care, and pregnancy outcomes in Lagos, Nigeria.	Guided by the social cognitive theory and health belief model, the purpose of this cross-sectional quantitative study was to determine if there is a relationship between maternal health literacy, antenatal care visits, development of medical conditions during pregnancy, and pregnancy outcomes (measured by healthy or unhealthy baby) in Lagos, Nigeria.
Adedimeji et al.[197]	Nigeria	2017	Improving outcomes in cancer diagnosis, prevention and control: barriers, facilitators and the need for health literacy in Ibadan Nigeria.	Studies suggest increasing health literacy and empowering individuals to take preventive action will improve outcomes and mitigate impact on a weak health system.
Adefabi[198]	Nigeria	2018	Inclusive economic growth: the pathway to sustainable development	This paper therefore examines conceptual issues in inclusive economic growth and submits that among its major impediments in Nigeria are deficient physical infrastructure, bleak economic situation and financial exclusion, among others
Adekoya-Cole et al.[199]	Nigeria	2015	Poor Health Literacy in Nigeria: Causes, Consequences and Measures to improve it.	This review aims to discuss the factors that influence health literacy, effects of low health literacy on the individual and on the community and to proffer measures on how to improve health literacy in our communities
Ajuwon and Ajuwon[200]	Nigeria	2019	Teaching high school students to use online consumer health resources on mobile phones: outcome of a pilot project in Oyo State, Nigeria.	This project evaluated the outcomes of training high school students to deliver consumer health information to their peers.
Akangbe et al.[201]	Nigeria	2015	An assessment of health practises among small-scale farmers in Kwara State, Nigeria.	The study examines the personal health practices of small scale farmers in Kwara State, Nigeria.
Al-Mujtaba et al.[202]	Nigeria	2020	Assessing the acceptability of village health workers' roles in improving maternal health care in Gombe State, Nigeria a qualitative exploration from women beneficiaries.	We assessed the acceptability of VHW services among women beneficiaries of the Program.
Aluh et al.[203]	Nigeria	2018	Mental health literacy among Nigerian teachers.	This study aims to assess mental health literacy among teachers with focus on their knowledge of depression.
Aluh et al.[204]	Nigeria	2019	Cross-sectional survey of mental health literacy among undergraduate students of the University of Nigeria.	This study sought to assess knowledge of schizophrenia and help-seeking behaviour among undergraduate students of a Nigerian university. Sociodemographic predictors of correct recognition were also explored.

Aluh et al.[205]	Nigeria	2020	Comparison of pharmacists' mental health literacy: Developed versus developing countries.	To compare the mental health literacy (MHL) of pharmacists in Nigeria and their comfort rendering pharmacy services to patients with mental illness with that of pharmacists in developed countries.
Aluh et al.[203]	Nigeria	2018	Mental health literacy: what do Nigerian adolescents know about depression?	While there is a growing literature on the mental health literacy of adults, there has not been a parallel interest in the mental health literacy of young people in Nigeria.
Anchang & Mbunwe [206]	Nigeria	2019	A stated preference discrete choice health literacy intervention framework for the control of non-communicable diseases (NCDs) in Africa.	Not specified
Arulogun et al.[207]	Nigeria	2016	Experience of Using an Interdisciplinary Task Force to Develop a Culturally Sensitive Multipronged Tool to Improve Stroke Outcomes in Nigeria.	This paper describes the unique experience in Sub-Saharan Africa of utilizing of an interdisciplinary Task Force to facilitate the development of the multipronged behavioral intervention aimed at enhancing stroke outcomes in a low-middle income country.
Atilola and Olayiwola[208]	Nigeria	2012	The Nigerian home video boom: should Nigerian psychiatrists be worried? Lessons from content review and views of community dwellers.	To assess the popularity of Nigerian home videos among Nigerian community dwellers and the frequency of their exposure to scenes depicting mental illness.
Atiloloa and Olayiwola[209]	Nigeria	2011	Stigmatisation of mental illness in Nigerian home videos.	In an effort to answer these questions, community dwellers participating in a 2009 public health campaign in Ibadan, Nigeria, were asked via in-person interviews how often they see scenes depicting “madness” in Nigerian films and about their views of the accuracy of such depictions.
Bella-Awusah et al.[210]	Nigeria	2014	The impact of a mental health teaching programme on rural and urban secondary school students' perceptions of mental illness in southwest Nigeria.	This study aimed to assess the impact of a school based mental health awareness programme aimed at increasing mental health literacy and reducing negative views about persons with mental illness.
Dogra et al.[211]	Nigeria	2012	Nigerian secondary school children's knowledge of and attitudes to mental health and illness.	To establish the views and knowledge about mental health and illness in pupils at four secondary schools in rural and urban Southwest Nigeria.
Eguzo and Camazine[212]	Nigeria	2013	Beyond limitations: practical strategies for improving cancer care in Nigeria.	Despite the rising incidence and public health importance, Nigeria lacks an organized and comprehensive strategy to deal with cancers.(...) This review led to the formulation of a proposal for Nigerian National Cancer Policy, mainly drawn from effective strategies used in Canada, Brazil and Kenya.
Etokidem et al.[213]	Nigeria	2021	Potential barriers to and facilitators of civil society organization engagement in increasing immunization coverage in Odukpani Local Government Area of Cross River State, Nigeria: an implementation research.	This study explored the potential barriers to and facilitators of CSO engagement in increasing immunization coverage in Odukpani Local Government Area of Cross River State, Nigeria.
Eze et al.[214]	Nigeria	2016	Public's knowledge of the differences between ophthalmologists and optometrists: a critical issue in eye care service utilisation.	To assess the public's knowledge of the differences between ophthalmologists and optometrists and identify the factors associated with knowledge.
Famuyiwa and Entwistle[215]	Nigeria	2021	Characterising and communicating the potential hazard posed by potentially toxic elements in indoor dusts from schools across Lagos, Nigeria.	The aim of the study was to assess the potential hazard posed by PTE in indoor dusts and to develop a suitable risk communication strategy to inform and educate the public, promoting environmental health literacy.
Gabriel et al.[216]	Nigeria	2021	Feasibility of a socio-spiritual intervention to improve quality of life of adult Nigerians with cancer and their family caregivers: Protocol for a randomised controlled trial.	This protocol tests the feasibility of a randomised controlled trial on the efficacy of a socio-spiritual intervention to improve the quality of life of adult Nigerians living with cancer and their family caregivers.
Gabriel et al.[217]	Nigeria	2021	Quality of life and associated factors among adults living with cancer and their family caregivers.	This study examined the association of needs, health literacy, and quality of life among adult Nigerians with cancer and family caregivers

Hamilton-Ekeke et al.[218]	Nigeria	2020	Health Literacy in the Promotion of Wellness among Secondary School Students in Bayelsa State, Nigeria	The study determined the level of health literacy in terms of knowledge of drug abuse as well as its application in making healthy decisions among secondary school students in Ogbia Local Government Area of Bayelsa State, Nigeria
Igwesi-Chidobe et al.[219]	Nigeria	2020	Evidence, theory and context: using intervention mapping in the development of a community-based self-management program for chronic low back pain in a rural African primary care setti~~ng~~ g - the good back program.	This paper presents the application of the intervention mapping (IM) approach in the development of a complex behavior change intervention - The Good Back program, aimed at reducing non-specific chronic low back pain (CLBP) disability in rural Nigeria.
Ikwuka et al.[220]	Nigeria	2016	Ideological vs. Instrumental Barriers to Accessing Formal Mental Health care in the Developing World: Focus on South-eastern Nigeria.	The study aimed to establish the relative weight, significance and determinants of instrumental and ideological barriers for prioritised policy interventions
Kuyinu et al.[221]	Nigeria	2020	Health literacy: Prevalence and determinants in Lagos State, Nigeria.	The study was conducted to measure the prevalence of health literacy and its determinants among Lagos State residents.
Liu et al.[222]	Nigeria	2020	Enhancing Knowledge in Informal Settlements: Assessing Health Beliefs and Behaviors in Nigeria: A cross-sectional survey assessment of perceptions, practices, and resources in underserved urban communities in Lagos.	This project aimed to determine the baseline level of health literacy, behavioral practices, and accessibility to resources in a set of 16 informal settlements located around Lagos, Nigeria in order to identify topics that should be emphasized in a new teaching curriculum directed at local Community Health Educators.
Mojoyinola[223]	Nigeria	2011	Influence of Maternal Health Literacy on Healthy Pregnancy and Pregnancy Outcomes of Women Attending Public Hospitals in Ibadan, Oyo State, Nigeria Abstract ...	The present study investigated the influence of maternal health literacy on healthy pregnancy and pregnancy outcomes
Nwaozuru et al.[224]	Nigeria	2020	Addressing Stroke Literacy in Nigeria Through Music: A Qualitative Study of Community Perspectives	We sought to understand community-level perspectives on using African music to promote acute stroke literacy.
Obaremi and Olatokun[225]	Nigeria	2021	A survey of health information source use in rural communities identifies complex health literacy barriers.	The study assessed sources and challenges concerning health information access and use among residents of five rural communities in Ibadan, Nigeria.
Obasola and Mabawonku[226]	Nigeria	2018	Mothers' perception of maternal and child health information disseminated via different modes of ICT in Nigeria.	To investigate mothers' perceived usefulness of ICT and MCH information disseminated through e-health projects in Nigeria.
Ofole and Ohakwe[227]	Nigeria	2021	Therapeutic Outcome of Self-Control and Social Interaction Interventions on Negative Body Image among In-School Adolescents with Low Health Literacy in Southwest Nigeria	This study examined the effectiveness of Self-Control Therapy (SCT) and Social Interaction Skills Training (SIST) in managing negative body image among senior secondary school students with low health literacy in Southwest Nigeria.
Ogunrin et al.[228]	Nigeria	2019	Genomic Literacy and Awareness of Ethical Guidance for Genomic Research in Sub-Saharan Africa: How Prepared Are Biomedical Researchers?	We explored the level of preparedness of biomedical researchers in a sub-Saharan African country using in-depth interviews to obtain data on their understanding of genomics and genomic research and assess their awareness of the scope of the country's code of health research ethics.
Ohaeri and Fido[229]	Nigeria	2001	The opinion of caregivers on aspects of schizophrenia and major affective disorders in a Nigerian setting.	To assess the opinion of relatives of 75 schizophrenics and 20 major affective disorder cases on aspects of the disease and compare with the responses of relatives of cancer, infertility and sickle cell disease (SCD) cases.

Oladunjoye et al.[230]	Nigeria	2013	Health Literacy Amongst Tuberculosis Patient in a General Hospital	This study aims to bridge the existing knowledge gap by assessing health literacy among patients with TB in a rural town in Northern Nigeria.
Olusegun[231]	Nigeria	2019	The Roles of Yoruba Songs on Pregnancy, Labour and Baby Care in Antenatal and Postnatal Clinic in Southwestern Nigerian Hospital	This paper therefore, examines the roles of Yoruba health related songs on pregnancy, labour, delivery and baby care during the health literacy classes in selected hospitals in southwestern Nigeria
Sokefun and Atulomah[232]	Nigeria	2020	Predictors of infant-survival practices among mothers attending paediatric clinics in Ijebu-Ode, Ogun State, Nigeria.	This study was undertaken to provide better understanding of the dynamics of predictors of infant-survival practices among mothers with infants attending paediatric clinics.
Ukpabi [233]	Nigeria	2021	Relationship among health literacy, superstitious/cultural beliefs, and self-care among diabetic patients in Warri, Nigeria.	This study was conducted to determine the moderating effects of superstitious or cultural beliefs on the relationship between health literacy and self-care adoption among diabetes patients in Warri, Nigeria.
Ukwenya et al.[234]	Nigeria	2021	COVID-19 health literacy, coping strategies and perception of COVID-19 containment measures among community members in a southwestern state in Nigeria.	This study aimed to describe COVID-19 health literacy, coping strategies and perception of COVID-19 containment measures among community members in a Southwestern state in Nigeria.
Uwatt et al.[235]	Nigeria	2010	Literacy and Health Seeking Behaviours among Patients in Benue and Cross River States of Nigeria	determine the relationship between outpatients literate status and their health seeking behaviour
Väisänen et al.[236]	Nigeria	2021	Sexual and Reproductive Health Literacy, Misoprostol Knowledge and Use of Medication Abortion in Lagos State, Nigeria: A Mixed Methods Study.	Little is known about the link between health literacy and women's ability to safely and successfully use misoprostol to self-induce an abortion.
Mbada et al.[237]	Nigeria (Yoruba)	2022	Cultural adaptation and psychometric evaluation of the Yoruba version of the Health Literacy Questionnaire.	The objective of this study was to culturally adapt and establish the psychometric properties of the Yoruba version of the health literacy Questionnaire.
Cubaka et al.[238]	Rwanda	2018	He should feel your pain': Patient insights on patient-provider communication in Rwanda.	This study explored patients' communication preferences and perceptions on what factors influence the patient-provider communication in primary health care settings in Rwanda.
Ingabire et al.[239]	Rwanda	2016	Using an intervention mapping approach for planning, implementing and assessing a community-led project towards malaria elimination in the Eastern Province of Rwanda.	This paper describes development, implementation and evaluation of a community-based malaria elimination project in Ruhuha sector, Bugesera district, Eastern province of Rwanda.
Linden et al.[240]	Rwanda	2016	Validation of a community-based survey assessing nonobstetric surgical conditions in Burera District, Rwanda.	The goal of this study was to create and assess the validity of a community-based questionnaire collecting data on untreated surgically correctable disease throughout Burera District, Rwanda, to accurately plan for surgical services at a district hospital.
Lygidakis et al.[241]	Rwanda	2019	Community- and mHealth-based integrated management of diabetes in primary healthcare in Rwanda (D²Rwanda): the protocol of a mixed-methods study including a cluster randomised controlled trial.	The D²Rwanda study aims at: (a) determining the efficacy of an integrated programme for the management of diabetes in Rwanda, which will provide monthly patient assessments by HBCPs, and an educational and self-management mHealth patient tool, and; (b) exploring qualitatively the ways the interventions will have been enacted, their challenges and effects, and changes in the patients' health behaviours and HBCPs' work satisfaction.
Mukanoheli et al.[242]	Rwanda	2020	Functional Health Literacy and Self-Care Behaviors Among Type 2 Diabetic Patients at a University Teaching Hospital in Kigali Abstract PDF ...	To determine if functional health literacy is associated with self-care behaviors among T2DM patients.

Umubyeyi et al.[243]	Rwanda	2016	Help-seeking behaviours, barriers to care and self-efficacy for seeking mental health care: a population-based study in Rwanda.	Mental disorders commonly affect young people but usually go unrecognized and untreated. This study aimed to investigate help-seeking behaviours, barriers to care and self-efficacy for seeking mental health care among young adults with current depression and/or suicidality in a low-income setting.
Dieng et al.[244]	Senegal	2020	Mothers' oral health literacy and children's oral health status in Pikine, Senegal: A pilot study.	The objective of this study is to estimate the level of mothers' OHL in Senegal and its relation to the dental health of their children.
Glik et al.[245]	Senegal	2016	Health-related media use among youth audiences in Senegal.	The purpose of this study was to better understand how younger audiences are navigating traditional and newer forms of media technologies, with particular emphasis on the skills and competencies needed to obtain, evaluate and apply health-related information, also defined as health and media literacy.
Kim et al.[246]	Senegal	2019	The Role of Health Literacy in Family Planning Use among Senegalese Women.	In this research, we investigate health literacy as an auxiliary component of health communication. We test the validity of the health literacy Skills Framework by examining the correlation of health literacy indicators to family planning use among Senegalese women in the 2014 Demographic Health Survey
Kennedy et al.[247]	Sierra Leone	2022	Factors associated with adherence to treatment in patients with open angle glaucoma in Sierra Leone, West Africa: patient demographics and questionnaire.	We aim to establish the factors contributing to late presentation, treatment non-adherence and disease progression in glaucoma patients in Sierra Leone.
Keles et al.[248]	Somalia	2021	Clinical characteristics of acute liver failure associated with hepatitis A infection in children in Mogadishu, Somalia: a hospital-based retrospective study.	The aim of the study was therefore to investigate the Hepatitis A infection and its rare complication of acute liver failure in children in Somalia.
Aggarwal et al.[249]	South Africa	2017	Evaluation of modified patient health questionnaire-9 teen in South African adolescents.	This report describes the findings of the survey conducted by South African Depression and Anxiety Group (SADAG) in a cohort of disadvantaged South African adolescent scholars in which PHQ-9 teen version was modified and adapted (PHQ-9M) to improve the response rate.
Aggarwal et al.[250]	South Africa	2016	South African adolescents' beliefs about depression.	This study explores the depression literacy in adolescents in South Africa.
Andersson et al.[251]	South Africa	2013	Help-seeking behaviour, barriers to care and experiences of care among persons with depression in Eastern Cape, South Africa.	Little is known about the help-seeking behaviour and barriers to care among people with depression in poor resource settings in Sub-Saharan Africa.
Babatunde et al.[252]	South Africa	2020	Stakeholders' perceptions of child and adolescent mental health services in a South African district: a qualitative study.	Hence this study sought to explore multisectoral dynamics in providing CAMH care in one resource-constrained South African district as a case study, towards informing the development of a model for district mental health plan and generating lessons for mental health systems strengthening to support CAMH services using the Health Systems Dynamics (HSD) framework.
Babatunde et al.[253]	South Africa	2022	Identifying multilevel and multisectoral strategies to develop a Theory of Change for improving child and adolescent mental health services in a case-study district in South Africa.	Together with key stakeholders across multiple sectors, this study aims to (i) co-identify causal factors and potential strategies to overcome bottlenecks in one district in SA as a case study; and (ii) Co-develop a Theory of Change (ToC) for increasing access to CAMH services within the resource constraints of a remote resource-scarce district as a case study.
Bennin and Rother[254]	South Africa	2017	"But it's just paracetamol": Caregivers' ability to administer over-the-counter painkillers to children with the information provided.	Objective: To determine whether caregivers are able to make informed decisions about their families' use of over-the-counter (OTC) painkillers through access to and use of three mechanisms of information provision.
Bobbins et al.[255]	South Africa	2019	Balanced nutrition and hand hygiene for children in South Africa.	The aim of this research was to design and implement a participatory health promotion intervention for caregivers at the Rhodes Day Care Centre (RDCC) that highlighted these important public health issues.
Braathen et al.[256]	South Africa	2013	Understanding the local context for the application of global mental health: a rural South African experience.	It is only through rich and detailed understandings of local contexts and individual experiences that the challenges global mental health faces can be fully appreciated.

Campbell et al.[257]	South Africa	2021	Evaluating Community Engagement Strategies to Manage Stigma in Two African Genomics Studies Involving People Living with Schizophrenia or Rheumatic Heart Disease.	This article reports on community engagement evaluation strategies in two African genomics studies: the Stigma in African Genomics Research study and the Genomics of Schizophrenia in South African Xhosa People (SAX) study.
Clarke and Voss[258]	South Africa	2016	The role of a multidisciplinary student team in the community management of chronic obstructive pulmonary disease.	To determine whether a community-based, multidisciplinary team consisting of home-based caregivers and supervised students could improve the functional status and quality of life of patients living with chronic obstructive pulmonary disease (COPD) in a low-income, peri-urban setting in South Africa
Davis and Jansen[259]	South Africa	2021	Deploying a Fotonovela to Combat Methamphetamine Abuse among South Africans with Varying Levels of Health Literacy.	In the first part of a two-part study, we compared a health-related fotonovela about MA to an existing brochure group and a control group.
De et al[260]	South Africa	2012	Maximizing health literacy and client recall in a developing context: speech-language therapist and client perspectives.	This study aimed to investigate (1) issues around clients' health literacy and recall of information; and (2) how these issues can be overcome in speech-language therapy in a developing context.
Sorsdahl et al.[261]	South Africa	2010	Explanatory models of mental disorders and treatment practices among traditional healers in Mpumalanga, South Africa.	The present study examines traditional healers' explanatory models (EMs) and treatment practices for psychotic and non-psychotic mental illnesses.
Devillé and Tempelman[262]	South Africa	2019	Feasibility and robustness of an oral HIV self-test in a rural community in South-Africa: An observational diagnostic study.	The primary objective of this study was to evaluate the ability of untrained persons to correctly interpret the OraQuick HIV Self-Test results with oral fluid compared with results obtained by trained users using the matched lot OraQuick Rapid HIV-1/2 Antibody Test and blinded to the results of the Self-Test.
Dowse et al.[263]	South Africa	2010	Applicability of the REALM health literacy test to an English second-language South African population.	To investigate health literacy in an English second language population using the REALM test, to evaluate its appropriateness and to compare health literacy between four different education categories.
Ducray et al.[264]	South Africa	2021	Cervical cancer knowledge and screening uptake by marginalized population of women in inner-city Durban, South Africa: Insights into the need for increased health literacy.	This study explored the levels of knowledge and screening rates of cervical cancer among vulnerable women living in the inner-city of Durban, South Africa.
Engelbrecht et al.[265]	South Africa	2022	Factors Associated with Limited Vaccine Literacy: Lessons Learnt from COVID-19.	Therefore, this study aimed to assess levels of VL among the adult population in South Africa, as well as to identify factors associated with limited VL.
Etheredge et al.[266]	South Africa	2018	Opt-in or opt-out to increase organ donation in South Africa? Appraising proposed strategies using an empirical ethics analysis.	we evaluate the merits of systems proposed to increase deceased organ donation in South Africa (SA)
Field et al.[267]	South Africa	2019	Accessible continued professional development for maternal mental health	The aim of this study was to determine whether the Bettercare Maternal Mental Health book significantly improves knowledge and decreases stigma around mental health for care providers from the health and social development sectors.
Fincham et al.[268]	South Africa	2008	Dietary and Fluid Adherence among Haemodialysis Patients Attending Public Sector Hospitals in the Western Cape	The purpose of this paper was to determine the applicability of the Theory of Planned Behaviour (TPB) in predicting dietary and fluid adherence among a sample of haemodialysis patients attending public sector hospitals in the Western Cape.
Galappaththi-Arachchige et al.[269]	South Africa	2018	Reproductive health problems in rural South African young women: risk behaviour and risk factors.	This study seeks to examine the underlying factors that may be associated with these four adverse reproductive health outcomes.
Gordon et al.[270]	South Africa	2020	Socio-economic inequalities in the multiple dimensions of access to healthcare: the case of South Africa.	This paper provides a diagnosis of the extent of socio-economic inequalities in health and healthcare using an integrated conceptual framework.
Haricharan et al.[271]	South Africa	2017	Health promotion via SMS improves hypertension knowledge for deaf South Africans.	Additionally, the study aimed to assess the acceptability of using SMSs for health promotion targeting Deaf people.

Hathorn et al.[272]	South Africa	2021	Help-Seeking Intention in Obsessive-Compulsive Disorder: Predictors and Barriers in South Africa.	Using the health belief model as a conceptual framework, study aims were to identify predictors of and barriers to help-seeking among South Africans with OCD
Hunter-Adams et al.[273]	South Africa	2017	A Qualitative study of language barriers between South African health care providers and cross-border migrants.	In this article, we explore the complexity of health communication from the perspective of cross-border migrants seeking antenatal care in Cape Town, South Africa in order to highlight the importance of high quality medical interpretation.
Hussain-Alkhateeb et al.[274]	South Africa	2015	Local perceptions of causes of death in rural South Africa: a comparison of perceived and verbal autopsy causes of death.	This study describes the agreement between VA-derived causes of death and informant-perceived causes and associated influential factors, which also reflects lay health literacy in this setting.
Joubert and Githinji[275]	South Africa	2014	Quality and readability of information pamphlets on hearing and paediatric hearing loss in the Gauteng Province, South Africa.	The objectives of this study were therefore to determine the availability of information pamphlets on hearing and hearing loss in children at public hospitals in the Gauteng Province of South Africa. In addition, the quality and readability levels of these pamphlets were determined.
Kagee[276]	South Africa	2008	Adherence to antiretroviral therapy in the context of the national roll-out in South Africa: Defining a research agenda for psychology.	I set a tentative agenda for psychosocial research with a view to enhancing the likelihood of optimal adherence among AIDS patients.
Keikelame and Swartz[277]	South Africa	2013	Lost opportunities to improve health literacy: observations in a chronic illness clinic providing care for patients with epilepsy in Cape Town South Africa.	We used observation to gain a picture of interactions between patients with epilepsy and health care providers in a chronic illness clinic serving the marginalized population groups in Cape Town.
Kemp et al.[278]	South Africa	2021	Pushing the bench: A mixed methods study of barriers to and facilitators of identification and referral into depression care by professional nurses in KwaZulu-Natal, South Africa.	This study explored barriers to and facilitators of nurse identification and referral of patients with depressive symptoms as part of integrated mental health service delivery in KwaZulu-Natal, South Africa.
Kometsi et al.[279]	South Africa	2020	Mental health literacy: Conceptions of mental illness among African residents of Sisonke District in KwaZulu-Natal, South Africa.	This study investigated conceptions and aetiological beliefs about mental illness among 787 randomly selected African residents of Sisonke District using a survey
Korhonen et al.[280]	South Africa	2019	Content validation of Mental Health Literacy Scale (MHLS) for primary healthcare workers in South Africa and Zambia ― a heterogeneous expert panel method.	This study's aim was to adapt the content validity of the Mental health literacy Scale (MHLS) developed by O'Connor & Casey (2015) with researchers and primary health-care workers in low- and middle-income contexts in South Africa (SA) and in Zambia
Kubheka et al.[281]	South Africa	2020	Social media health promotion in South Africa: Opportunities and challenges.	The aim of the review was conduct an analysis on the opportunities and challenges of the use of social media for health promotion in South Africa.
Lopes and McKay[282]	South Africa	2020	Adult Learning and Education as a Tool to Contain Pandemics: The COVID-19 Experience	This article explains why ALE, especially the promotion of health literacy as part of ALE (which is itself part of lifelong learning), is necessary to enable individuals to make informed health-related decisions.
Lothe et al.[283]	South Africa	2018	Treating schistosomiasis among South African high school pupils in an endemic area, a qualitative study.	This study aims to explore attitudes towards anti- schistosomal treatment.
Madlala et al.[284]	South Africa	2022	Community mental health literacy in Tshwane region 1: A quantitative study.	To assess the literacy of three mental disorders, namely major depressive disorder (MDD), schizophrenia and generalised anxiety disorder (GAD) and to compare the resultant assumed literacy level between urban and townships participants.
Mafutha et al.[285]	South Africa	2017	Development of a Hypertension Health Literacy Assessment Tool for use in primary healthcare clinics in South Africa, Gauteng.	To develop a Hypertension Heath Literacy Assessment Tool to establish patients' comprehension of the health education they receive in primary healthcare (PHC) clinics in Tshwane, Gauteng, South Africa.

Mall et al.[286]	South Africa	2013	Mental health in primary human immunodeficiency virus care in South Africa: a study of provider knowledge, attitudes, and practice.	Two vignettes portraying HIV patients with depression and substance use (specifically problematic alcohol use) were presented to respondents to investigate their mental health literacy and attitudes toward mental disorders.
Manhanzva et al.[287]	South Africa	2017	Gender and leadership for health literacy to combat the epidemic rise of noncommunicable diseases.	This case study highlights the burden of disease
Masuku et al.[288]	South Africa	2018	I felt pain. Deep pain…': Experiences of primary caregivers of stroke survivors with aphasia in a South African township.	The aim of the study was to describe the caregiving experience of female caregivers of PWA residing in Tembisa, a township situated in the east of Johannesburg.
Matima et al.[289]	South Africa	2018	A qualitative study on the experiences and perspectives of public sector patients in Cape Town in managing the workload of demands of HIV and type 2 diabetes multimorbidity.	how patients with chronic multimorbidities currently experience the (re)-organisation of health services and what their perceived needs are
Medina-Marino et al.[290]	South Africa	2020	The role of trust and health literacy in nurse-delivered point-of-care STI testing for pregnant women living with HIV, Tshwane District, South Africa.	we explored the role patient-provider communications may play in supporting treatment adherence and STI disclosure to sexual partners.
Mhlongo et al.[291]	South Africa	2018	Health education on diabetes at a South African national science festival	To determine the effects of a health education programme on increasing knowledge about diabetes and encouraging preventive measures.
Minty et al.[292]	South Africa	2021	Mental illness attitudes and knowledge in non-specialist medical doctors working in state and private sectors.	To investigate aspects of knowledge and attitudes towards mental illness of a group of private and state-employed non-specialist medical doctors.
Mohamed-Kaloo and Lather[293]	South Africa	2014	Perceptions of mental illness among Muslim general practitioners in South Africa.	To investigate perceptions of mental illness in a sample of ten South African Muslim GPs (five male, five female) in the Lenasia area (Johannesburg, South Africa).
Mokwena and Ndlovu[294]	South Africa	2021	Why Do Patients with Mental Disorders Default Treatment? A Qualitative Enquiry in Rural Kwazulu-Natal, South Africa.	The objective of this study was to determine the reasons for treatment defaulting at Manguzi Hospital, KwaZulu-Natal Province, South Africa.
Molete et al.[295]	South Africa	2013	Oral health promotion in Gauteng: a qualitative study.	This study aimed to assess how health promoters in Gauteng integrate oral health into their general health promotion activities.
Monnapula-Mazabane and Petersen[296]	South Africa	2021	Mental health stigma experiences among caregivers and service users in South Africa: a qualitative investigation.	The study aimed to understand the nature and context of mental health stigma among people living with a mental health condition and the subsequent effect on their caregivers.
Moodley et al.[297]	South Africa	2020	They are inconveniencing us' - exploring how gaps in patient education and patient centred approaches interfere with TB treatment adherence: perspectives from patients and clinicians in the Free State Province, South Africa.	We explored clinician and patient perspectives of the gaps in TB messaging that influence TB treatment LTFU.
Moroe[298]	South Africa	2020	Occupational noise induced hearing loss in the mining sector in South Africa: Perspectives from occupational health practitioners on how mineworkers are trained.	The aim of this study was to explore the perspectives of occupational health practitioners (OHPs) regarding education and training of mineworkers on occupational noise induced hearing loss (ONIHL) and its impact on mineworkers' health

Müller et al.[299]	South Africa	2019	Effects of a School-Based Health Intervention Program in Marginalized Communities of Port Elizabeth, South Africa (the KaziBantu Study): Protocol for a Randomized Controlled Trial.	The aim of the KaziBantu project is to assess the effect of a school-based health intervention package on risk factors for NCDs, health behaviors, and psychosocial health in primary school children in disadvantaged communities in Port Elizabeth, South Africa. In addition, we aim to test a workplace health intervention for teachers.
Murphy et al.[300]	South Africa	2015	A qualitative study of the experiences of care and motivation for effective self-management among diabetic and hypertensive patients attending public sector primary health care services in South Africa.	The aim of this study was to explore patients' current experiences of chronic care, as well as their motivation and capacity for self-management and lifestyle change.
Musakwa et al.[301]	South Africa	2021	Perceived barriers to the uptake of health services among first-year university students in Johannesburg, South Africa.	The study aimed to identify perceived barriers to the uptake of health services among young adults entering the tertiary education system in South Africa.
Naidoo and Taylor[302]	South Africa	2015	HIV health literacy, sexual behaviour and self-reports of having tested for HIV among students.	The HIV prevalence among young South African adults makes it important to understand their HIV knowledge, sexual behaviour and HIV counselling and testing (HCT) behaviour in this group. This paper presents the demographics, knowledge, sexual behaviour and cues to action as reported by sexually active students' who had HCT.
Noncungu and Chipps[303]	South Africa	2020	Information-seeking in first visit pregnant women in Khayelitsha, South Africa.	This study describes the health education needs, information barriers and health information-seeking behaviour of pregnant women on their first visit to antenatal clinics in a low-income setting in the Western Cape.
Norris et al.[304]	South Africa	2022	Building knowledge, optimising physical and mental health and setting up healthier life trajectories in South African women (*Bukhali*): a preconception randomised control trial part of the Healthy Life Trajectories Initiative (HeLTI).	Bukhali is the first individual randomised controlled trial in Africa to test the efficacy of a complex continuum of care intervention and forms part of the Healthy Life Trajectories Initiative (HeLTI) consortium implementing harmonised trials in Canada, China, India and SA
Patel and Dowse[305]	South Africa	2015	Understanding the medicines information-seeking behaviour and information needs of South African long-term patients with limited literacy skills.	To investigate medicine information-seeking behaviour and information needs in patients with limited literacy.
Petersen and Lund[306]	South Africa	2011	Mental health service delivery in South Africa from 2000 to 2010: one step forward, one step back.	To identify progress and challenges in mental healthcare in South Africa, as well as future mental health services research priorities.
Rensburg[307]	South Africa	2020	Levels of health literacy and English comprehension in patients presenting to South African primary healthcare facilities.	The aim of this was to investigate, describe and compare health literacy and English comprehension levels of PHC patients using three locally adapted REALM-R (SA) tools and the LAB.
Sande et al.[308]	South Africa	2007	The relationship between patient education and glycaemic control in a South African township.	To investigate if there is a relationship between patients' perceived diabetes education and their glycaemic control.
Selohilwe et al.[309]	South Africa	2019	Evaluating the role of levels of exposure to a task shared depression counselling intervention led by behavioural health counsellors: outcome and process evaluation.	This study evaluated the relationship between levels of exposure to a task-shared counselling intervention and psychosocial outcomes (depression, functional disability, internalised stigma and social support) in chronic care service users with comorbid depression in South Africa guided by the Medical Research Council process evaluation framework. Implementation and participant-level factors that promote greater exposure were also investigated.
Smythe et al.[310]	South Africa	2022	Access to health care for people with stroke in South Africa: a qualitative study of community perspectives.	We aimed to explore the perspectives and experiences of (people with stroke) PWS related to stroke care services to inform health system strengthening measures.
Sorsdahl et al.[311]	South Africa	2010	Perspectives towards mental illness in people living with HIV/AIDS in South Africa.	We aimed to investigate the attitudes of PLWHA in South Africa towards people with psychiatric disorders, as well as mental health literacy in relation to four major disorders (depression, schizophrenia, PTSD and substance abuse).

Sorsdahl et al.[312]	South Africa	2012	Negative attributions towards people with substance use disorders in South Africa: variation across substances and by gender.	Therefore, the present study examined the attributions made by the general South African population about people who use substances and whether these attributions differ by the type of substance being used, the gender of the person using the substance, or the characteristics of the person making the attribution.
Sorsdahl et al.[313]	South Africa	2012	Mental Health Services in South Africa: Scaling up and future directions	We review key evidence regarding the burden and risk factors for mental disorders in South Africa and crucial challenges for local mental health services and research.
Spedding et al.[314]	South Africa	2018	Pregnant women's mental health literacy and perceptions of perinatal mental disorders in the Western Cape, South Africa.	However, little is known about how pregnant women perceive mental disorders during this time, particularly in low- and middle-income countries such as South Africa.
Strecker et al.[315]	South Africa	2014	Health rights pamphlets: critical literacy and inclusive citizenship, South Africa.	The research reported here analyses how the pamphlets, coupled with directed training, strengthened skills, promoted critical literacy and supported inclusive citizenship
Surka et al.[316]	South Africa	2015	Knowledge and perceptions of risk for cardiovascular disease: Findings of a qualitative investigation from a low-income peri-urban community in the Western Cape, South Africa.	We investigated the knowledge and perceptions about risk for cardiovascular disease in a community.
Thutloa and Stroud[317]	South Africa	2013	Does active participation in health enhance health outcomes and health care delivery systems	This research report focuses on work produced as part of the first author's doctoral project, exploring the phenomenon of consumption of health resources for health citizenship in the private health insurance industry.
Treffry-Goatley et al. [318]	South Africa	2018	Community engagement with HIV drug adherence in rural South Africa: a transdisciplinary approach.	Our aim was to use narratives of people living with HIV on antiretroviral therapy (ART) to stimulate dialogue among the wider community and to encourage reflection on the contextual factors that influence ART adherence in this setting. We also wanted to explore whether exposure to the personal narratives might influence health literacy around HIV and ART.
Wasserman et al.[319]	South Africa	2010	Assessment of the English literacy level of patients in primary health. Care Services in Tshwane; Gauteng Province: Part 2	The aim of this study was to assess the English literacy levels of primary health care patients using the Learning Ability Battery (LAB) and the adapted Rapid Estimate of Adult Literacy, Revised (REALM-R) and to determine how the results of the adapted REALM-R correlate with those of the LAB.
Wasserman et al.[320]	South Africa	2010	Adaptation of the Rapid Estimate of Adult Literacy in Medicine Revised (REALM-R) to the South African context: Part 1	The purpose of this paper was to adapt and validate the Rapid Estimate of Adult Literacy in Medicine Revised (REALM-R) to the South African context.
Worley et al.[321]	South Africa	2009	Wellness programmes for persons living with HIV/AIDS: experiences from Eastern Cape province, South Africa.	This paper reviews components of wellness services for people living with HIV/AIDS including: voluntary counselling and testing, disclosure and prevention, ongoing counselling, health literacy and peer education, community- and home-based care, adherence support, and associated comprehensive care continuums.
Wouters et al.[322]	South Africa	2009	Public-sector ART in the Free State Province, South Africa: community support as an important determinant of outcome.	This study aimed to extend the current literature by investigating how immunological and virological responses to ART, measured at three points in time (after six, 12, and 24 months of ART), are influenced by patient characteristics (age, sex), health literacy (educational level and knowledge about HIV/AIDS), baseline CD4 cell count, baseline viral load, and three forms of community support
Zanoni et al.[323]	South Africa	2021	Development and validation of the HIV adolescent readiness for transition scale (HARTS) in South Africa.	In this study, we developed and validated a tool to identify adolescent transition readiness.
Atilola[324]	Sub-Saharan Africa	2016	Mental health service utilization in sub-Saharan Africa: is public mental health literacy the problem? Setting the perspectives right.	not explicitly specified
Atilola[325]	Sub-Saharan Africa	2015	Level of community mental health literacy in sub-Saharan Africa: current studies are limited in number, scope, spread, and cognizance of cultural nuances.	To conduct a systematic review of the findings of studies that have examined aspects of mental health literacy among community dwellers in sub-Saharan Africa.

Beia et al.[326]	Sub-Saharan Africa	2021	Changing men or changing health systems? A scoping review of interventions, services and programmes targeting men's health in sub-Saharan Africa.	Within these studies, we identify dominant conceptualisations of men and men's health and how these have influenced the design of men's health interventions and services.
Dzifa et al.[327]	Sub-Saharan Africa	2018	Predictors and outcome of systemic lupus erythematosus (SLE) admission rates in a large teaching hospital in sub-Saharan Africa.	This retrospective study examines characteristics and outcomes of hospitalized systemic lupus erythematosus patients over a two-year period and serves as a baseline for comparison for future studies to examine the outcomes with the provision of more dedicated care.
Mutebi et al.[328]	Sub-Saharan Africa	2020	Engaging Patients for Clinical Trials in Africa: Patient-Centered Approaches.	not explicitly specified
Okumu et al.[329]	Sub-Saharan Africa	2021	Alleviating psychological distress and promoting mental wellbeing among adolescents living with HIV in sub-Saharan Africa, during and after COVID-19.	our commentary highlights mental health challenges faced by ALHIV and their caregivers in SSA.
Sarfo and Ovbiagele[330]	Sub-Saharan Africa	2017	Stroke minimization through additive anti-atherosclerotic agents in routine treatment (SMAART): A pilot trial concept for improving stroke outcomes in sub-Saharan Africa.	In this conceptual article, we review the data supporting the rationale for a polypill to improve stroke outcomes in SSA and propose the conduct of a Stroke Minimization through Additive Anti-atherosclerotic Agent in Routine Treatment (SMAART) pilot study to determine the impact of a polypill such as the Polycap DS® in reducing future vascular risk compared to usual care in recent stroke in SSA.
Thompson et al.[331]	Sub-Saharan Africa	2015	The health literacy needs of women living with HIV/AIDS	The purpose of the study was to explore and describe the health literacy needs of women living with HIV
Wiginton et al.[332]	Sub-Saharan Africa	2022	Hearing From Men Living With HIV: Experiences With HIV Testing, Treatment, and Viral Load Suppression in Four High-Prevalence Countries in Sub-Saharan Africa.	There is a critical need to better understand facilitators of men's successful engagement with HIV services and assess if there are similarities across contexts.
Mohale et al.[333]	Sub-Saharan Africa, Australia	2017	Maternity health care: The experiences of Sub-Saharan African women in Sub-Saharan Africa and Australia.	The study aimed to examine the maternity experiences of Sub-Saharan African women who had given birth in both Sub-Saharan Africa and in Australia.
Bastien[334]	Tanzania	2009	Access, agency and ambiguity: communication about AIDS among young people in Northern Tanzania.	The aim of this paper is to build on study findings suggesting that significant differences exist between young people in- and out-of-school in exposure to AIDS-related information and communication in Kilimanjaro
Dalsmo et al.[335]	Tanzania	2021	"Now I Feel That I Can Achieve Something": Young Tanzanian Women's Experiences of Empowerment by Participating in Health Promotion Campaigns.	The aim of this qualitative study was to explore how some of these young Tanzanian women experience participating in health promotion campaigns
Higgins[336]	Tanzania	2014	Constructing Identities through Literacy Events in HIV/AIDS Education	This article examines the literacy events in HIV/AIDS education in Tanzania to investigate how they construct social identities for participants and to what extent they provide opportunities for critical health literacies
Higgins[337]	Tanzania	2010	Discursive Enactments of the World Health Organization's Policies: Competing Cultural Models in Tanzanian HIV/AIDS Prevention	This article seeks to expand this body of research by exploring how health policies and health literacies are languaged in HIV/AIDS educational sessions in Tanzania.
Hovland et al.[338]	Tanzania	2021	Experiences from cross-cultural collaboration in health campaigns in Tanzania: a qualitative study.	The aim of this study was to explore the participants' experiences with the cross-cultural collaboration in the planning and delivery of TICC's health campaigns.

Kutcher et al.[339]	Tanzania	2017	The African Guide: One Year Impact and Outcomes from the Implementation of a School Mental Health Literacy Curriculum Resource in Tanzania	Following training, survey data addressing teacher reported AG impact and MHL outcomes was collected at three time points over a one year period.
Kutcher et al.[340]	Tanzania	2016	A school mental health literacy curriculum resource training approach: effects on Tanzanian teachers' mental health knowledge, stigma and help-seeking efficacy.	not specified: Successful application of a school MHL curriculum resource may be an effective way to increase teacher MHL and therefore help to improve mental health outcomes for students.
Pallangyo et al.[341]	Tanzania	2020	Knowledge of cardiovascular risk factors among caretakers of outpatients attending a tertiary cardiovascular center in Tanzania: a cross-sectional survey.	In view of the projected low health literacy in Tanzania, we conducted this cross-sectional survey to assess for CVD risk knowledge and its associated factors among patient escorts.
Perri-Moore et al.[342]	Tanzania	2015	Using an eIMCI-Derived Decision Support Protocol to Improve Provider-Caretaker Communication for Treatment of Children Under 5 in Tanzania.	This study examines whether the use of mobile technology can leverage a standardized treatment protocol to improve the impact of counseling for children’s caretakers and result in better understanding of what needs to be done at home after the clinical visit.
Stone et al.[343]	Tanzania	2011	"I didn't know that ..." patient perceptions of print information, education, and communication related to HIV/AIDS treatment.	In this paper we present the results of those focus group discussions and introduce the print IEC materials as a pilot intervention in a Kiswahili-speaking setting where a need for additional health literacy exists
York et al.[344]	Tanzania	2015	Factors affecting community participation in the CDTI program in Morogoro, Tanzania.	To explore community-perceived factors related to participation in and sustainability of the CDTI program in southwest Tanzania.
Jaiteh et al.[345]	The Gambia	2019	Community perspectives on treating asymptomatic infections for malaria elimination in The Gambia.	This mixed methods study explored people's attitudes towards the reactive treatment of compound contacts of malaria cases with a 3-day course of dihydroartemisinin-piperaquine (DHAP), the socio-cultural representations of asymptomatic infections, and more specifically their treatment.
Touray et al.[346]	The Gambia	2018	Incidence and Outcomes after Out-of-Hospital Medical Emergencies in Gambia: A Case for the Integration of Prehospital Care and Emergency Medical Services in Primary Health Care.	Data on self-reported medical emergencies among adults in a selection of Gambian communities are presented in this report.
Akena et al.[347]	Uganda	2021	The effectiveness of a psycho-education intervention on mental health literacy in communities affected by the COVID-19 pandemic-a cluster randomized trial of 24 villages in central Uganda-a research protocol.	For the proposed study, we will determine effectiveness of a psycho-education intervention delivered by village health team (VHT) members.
Bakeera et al.[348]	Uganda	2009	Community perceptions and factors influencing utilization of health services in Uganda.	To explore community perceptions among three different wealth categories on factors influencing healthcare utilization in Eastern Uganda.
Cattamanchi et al.[349]	Uganda	2015	Health worker perspectives on barriers to delivery of routine tuberculosis diagnostic evaluation services in Uganda: a qualitative study to guide clinic-based interventions.	Health worker perspectives on barriers to improving TB diagnostic evaluation are critical for developing clinic-level interventions to improve guideline implementation.
Chang et al.[350]	Uganda	2018	Motivations of women in Uganda living with rheumatic heart disease: A mixed methods study of experiences in stigma, childbearing, anticoagulation, and contraception.	It is unclear whether this suboptimal contraception and anticoagulant use during pregnancy is due to lack of health system resources, limited health literacy, or social pressure to bear children.
Guttersrud et al.[351]	Uganda	2015	Measuring Maternal Health Literacy in Adolescents Attending Antenatal Care in Uganda: Exploring the	The purpose of this article is to test the requirement of "local independence" in the newly developed "Maternal health literacy" (MaHeLi) composite scale measuring health literacy in pregnant adolescents attending antenatal care.

			Dimensionality of the Health Literacy Concept Studying a Composite Scale.	
Jatho et al.[352]	Uganda	2021	Capacity building for cancer prevention and early detection in the Ugandan primary healthcare facilities: Working toward reducing the unmet needs of cancer control services.	We aimed to contribute to reducing the unmet needs of cancer prevention and early detection services in Uganda through capacity building.
Jatho et al.[353]	Uganda	2020	Socio-culturally mediated factors and lower level of education are the main influencers of functional cervical cancer literacy among women in Mayuge, Eastern Uganda.	We also assessed the factors associated with cervical cancer literacy and awareness about currently available cervical cancer preventive services.
Jones and Norton[354]	Uganda	2007	On the Limits of Sexual Health Literacy: Insights from Ugandan Schoolgirls	the questions to be addressed in this article are as follows: to what extent are the young women in our study informed of the risks, responsibilities, outcomes, and impacts of sexual actions? To what extent are these young women free from sexual abuse and discrimination, and can they pursue abstinence where appropriate? To what extent do these young women have the freedom and opportunity to embrace their sexuality and make choices concerning reproduction?
Kaddumukasa et al.[355]	Uganda	2021	Epilepsy beliefs and misconceptions among patient and community samples in Uganda.	The objective of the study was to characterize and compare the attitudes, beliefs, and perceptions about epilepsy across community and patient cohorts in Uganda.
Kendrick and Mutonyi[356]	Uganda	2007	Meeting the Challenge of Health Literacy in Rural Uganda: The Critical Role of Women and Local Modes of Communication	This article seeks to better understand the relation between local and traditional modes of communication and health literacy within the context of a rural West Nile community in Northern Uganda.
Lynch et al.[357]	Uganda	2019	Exploring patient experiences with and attitudes towards hypertension at a private hospital in Uganda: a qualitative study.	The ways in which patients' knowledge and attitudes toward hypertension determine their engagement with and adherence to available care, however, remains unclear
Miller et al.[358]	Uganda	2021	Not Enough Money and Too Many Thoughts: Exploring Perceptions of Mental Health in Two Ugandan Districts Through the Mental Health Literacy Framework.	The purpose of the overarching qualitative study was to explore community-level (i.e., FGDs) and individual-level (i.e., IDIs) understanding of the local conceptualizations, perceptions, and practices on health and social issues.
Miller et al.[359]	Uganda	2020	Representation of Mental Illness in Leading Ugandan Daily Newspapers: A Content Analysis.	This study investigated the coverage of mental illness in the two largest circulation newspapers in Uganda:
Naigaga et al.[360]	Uganda	2015	Measuring maternal health literacy in adolescents attending antenatal care in a developing country - the impact of selected demographic characteristics.	The aim of this paper is to describe how selected demographic characteristics 'explain' the observed variance in the distribution of maternal health literacy estimates in adolescents attending antenatal care in Uganda, as estimated by the 'Maternal health literacy scale'.
Nalukenge et al.[361]	Uganda	2019	Knowledge and causal attributions for mental disorders in HIV-positive children and adolescents: results from rural and urban Uganda.	We investigated knowledge and causal attributions for mental disorders in HIV-positive children and adolescents in rural and urban Uganda
Nsangi et al.[362]	Uganda	2020	Effects of the Informed Health Choices primary school intervention on the ability of children in Uganda to assess the reliability of claims about treatment effects: a cluster-randomised controlled trial.	We aimed to evaluate an intervention designed to teach primary school children to assess claims about the effects of treatments (ie, any action intended to maintain or improve health).
Nwosu et al.[363]	Uganda	2020	Influence of Caretakers' Health Literacy on Delays to Traumatic Brain Injury Care in Uganda.	This study seeks to determine the factors that impact TBI patient caretakers' health literacy and examine how these factors influence delays in care.

Okello et al.[364]	Uganda	2014	Mental health literacy among secondary school students in North and Central Uganda: a qualitative study.	The objective of this study was to explore the mental health of young people in secondary schools in Northern and Central Uganda.
Patterson et al.[365]	Uganda	2020	Acute gastrointestinal illness in an African Indigenous population: the lived experience of Uganda's Batwa.	Using a mixed methods approach, this study characterized the lived experience of acute gastrointestinal illness (AGI) in an Indigenous Batwa population in south-western Uganda.
Semakula et al.[366]	Uganda	2020	Effects of the Informed Health Choices podcast on the ability of parents of primary school children in Uganda to assess the trustworthiness of claims about treatment effects: one-year follow up of a randomised trial.	The overall aims of this follow-up study were to evaluate the impact of our intervention 1 year after it was administered, and to assess retention of learning and behaviour regarding claims about treatments.
Swahn et al.[367]	Uganda	2014	Demographic and psychosocial characteristics of mobile phone ownership and usage among youth living in the slums of Kampala, Uganda.	The purpose of this study is to determine the prevalence of mobile phone ownership and use in this high-risk population and to identify psychosocial characteristics that may differentiate those owning and using a phone from those who do not.
Wandera and Kasumba[368]	Uganda	2017	"Ebinyo"-The Practice of Infant Oral Mutilation in Uganda.	The paper explains reason for the persistence of the practice, and to further inform on IOM (infant oral mutilation) to health practitioners who were previously unaware of the practice.
Yantzi et al.[369]	Uganda	2019	The disease isn't listening to the drug': The socio-cultural context of antibiotic use for viral respiratory infections in rural Uganda.	To identify factors precipitating antibiotic misuse and discuss how to promote safe antibiotics use and curb antibiotic resistance.
Davies et al.[370]	Zambia	2009	Testing the health literacy of nurses in Zambia	This article describes an investigation of the literacy levels of three groups of Zambian nurses: Enrolled Nurses (two-year trained), Registered Nurses (three-year trained) and student Registered Nurses (post-initial training).
Mwambwa-Johnson[371]	Zambia	2021	Mental health literacy among rural and urban young adults in Zambia.	The purpose of this quantitative cross-sectional study was to investigate the relationship between levels of MHL and attitudes and beliefs about mental illness and health-seeking behaviors of Zambian young adults aged 18 to 24.
Schrauben and Wiebe[372]	Zambia	2015	Health literacy assessment in developing countries: a case study in Zambia.	We aimed to derive a measure of HL in data from the Demographic and Health Surveys (DHS) Program administered by the United States Agency for International Development (USAID), which includes items representing domains of HL as defined by the IOM
Sharpe et al.[373]	Zambia	2021	Mental health and wellbeing implications of the COVID-19 quarantine for disabled and disadvantaged children and young people: evidence from a cross-cultural study in Zambia and Sierra Leone.	This study examined mental health of CYP during the first COVID-19 lockdown in Zambia and Sierra Leone.
Topp et al.[374]	Zambia	2018	The health system accountability impact of prison health committees in Zambia.	We present findings from a nested evaluation of the impact of eight Prison Health Committees PrHCs 18 months after programme initiation.
Underwood et al.[375]	Zambia	2007	Health communication in multilingual contexts: a study of reading preferences, practices, and proficiencies among literate adults in Zambia.	This article reports the results of a survey of 2,009 literate Zambian adults who were tested for reading comprehension of health materials written at fourth- and eighth-grade levels.
Smythe et al.[376]	Zimbabwe	2022	A path toward disability-inclusive health in Zimbabwe Part 1: A qualitative study on access to healthcare.	Our objectives were to explore the experiences of people with disabilities in accessing care and identify opportunities for the health system to be designed for inclusion in Zimbabwe.
Terry et al.[377]	Zimbabwe	2005	HIV/AIDS health literacy in Zimbabwe–focus group findings from university students.	This qualitative study was designed to assess program needs and evaluate and improve HIV/AIDS prevention efforts at the University of Zimbabwe.

44. Azmat, S.K.; Ali, M.; Siddiqui, F.J.; Tirmizi, S.F.A.; Kiarie, J. Scoping Review on the Impact of Outbreaks on Sexual and Reproductive Health Services: Proposed Frameworks for Pre-, Intra-, and Postoutbreak Situations. *BioMed Research International* **2021**, 1–21, doi:10.1155/2021/9989478.
45. Ganasen, K.A.; Parker, S.; Hugo, C.J.; Stein, D.J.; Emily, R.A.; Seedat, S. Mental Health Literacy: Focus on Developing Countries. *African Journal of Psychiatry* **2008**, *11*, 23–28.
46. Gresh, A.; Cohen, M.; Anderson, J.; Glass, N. Postpartum Care Content and Delivery throughout the African Continent: An Integrative Review. *Midwifery* **2021**, *97*, 102976, doi:10.1016/j.midw.2021.102976.
47. Ogunbodede, E.O.; Kida, I.A.; Madjapa, H.S.; Amedari, M.; Ehizele, A.; Mutave, R.; Sodipo, B.; Temilola, S.; Okoye, L. Oral Health Inequalities between Rural and Urban Populations of the African and Middle East Region. *Advances in dental research* **2015**, *27*, 18–25, doi:10.1177/0022034515575538.
48. Park, M.B. The Effect of Advances in Transportation on the Spread of the Coronavirus Disease: The Last Is Africa and Endemic. *Journal of public health research* **2021**, *10*, doi:10.4081/JPHR.2021.2058.
49. Afolabi, O.A.; Nkhoma, K.; Maddocks, M.; Harding, R. What Constitutes a Palliative Care Need in People with Serious Illnesses across Africa? A Mixed-Methods Systematic Review of the Concept and Evidence. *Palliative medicine* **2021**, *35*, 1052–1070, doi:10.1177/02692163211008784.
50. Chidzonga, M.M.; Carneiro, L.C.; Kalyanyama, B.M.; Kwamin, F.; Oginni, F.O. Determinants of Oral Diseases in the African and Middle East Region. *Advances in Dental Research* **2015**, *27*, 26–31, doi:10.1177/0022034515581645.
51. Lucero-Prisno, D.E.; Adebisi, Y.A.; Lin, X. Current Efforts and Challenges Facing Responses to 2019-nCoV in Africa. *Global Health Research and Policy* **2020**, *5*, 21, doi:10.1186/s41256-020-00148-1.
52. Vearey, J.; Luginaah, I.; Magitta, N.F.; Shilla, D.J.; Oni, T. Urban Health in Africa: A Critical Global Public Health Priority. *BMC public health* **2019**, *19*, doi:10.1186/S12889-019-6674-8.
53. Ouedraogo, I.; Some, M.J.; Benedikter, R.; Diallo, G. A Systematic Review on Improving Health Literacy in Rural Africa Using Mobile Serious Games. *Studies in health technology and informatics* **2022**, *295*, 140–143, doi:10.3233/SHTI220681.
54. Ezenwankwo, E.F.; Ogbodo, V.E.; Alom, G.O.; Nwadilibe, I.B.; Ofodum, C.M.; Nwankwo, C.A.; Okigbo, C.C.; Omeje, C.A.; Onyebuchi, S.J.; Oladoyimbo, C.A.; et al. Behavioural Oncology Research in Africa: Lessons from the Last Two Decades and Key Considerations Moving Forward. *European journal of cancer care* **2022**, *31*, e13545, doi:10.1111/ecc.13545.
55. Okereke, M.; Ukor, N.A.; Ngaruiya, L.M.; Mwansa, C.; Alhaj, S.M.; Ogunkola, I.O.; Jaber, H.M.; Isa, M.A.; Ekpenyong, A.; Lucero-Prisno, D.E. COVID-19 Misinformation and Infodemic in Rural Africa. *The American Journal of Tropical Medicine and Hygiene* **2021**, *104*, 453–456, doi:10.4269/ajtmh.20-1488.
56. Kickbusch, I. Health Iteracy: Addressing the Health and Education Divide. *Health promotion international* **2001**, *16*.
57. Acheampong, M.; Ejiofor, C.; Salinas-Miranda, A.; Wall, B.; Yu, Q. Priority Setting towards Achieving Under-Five Mortality Target in Africa in Context of Sustainable Development Goals: An Ordinary Least Squares (OLS) Analysis. *Global health research and policy* **2019**, *4*, doi:10.1186/S41256-019-0108-0.
58. Petersen, I.; Marais, D.; Abdulmalik, J.; Ahuja, S.; Alem, A.; Chisholm, D.; Egbe, C.; Gureje, O.; Hanlon, C.; Lund, C.; et al. Strengthening Mental Health System Governance in Six Low- and Middle-Income Countries in Africa and South Asia: Challenges, Needs and Potential Strategies. *Health Policy and Planning* **2017**, *32*, 699–709, doi:10.1093/heapol/czx014.
59. Basu, L.; Frescas, R.; Kiwelu, H. Patient Guardians as an Instrument for Person Centered Care. *Globalization and health* **2014**, *10*, doi:10.1186/1744-8603-10-33.
60. Ramos, N.N.V.; Fronteira, I.; Martins, M.R.O. Building a Health Literacy Indicator from Angola Demographic and Health Survey in 2015/2016. *International journal of environmental research and public health* **2022**, *19*, doi:10.3390/IJERPH19052882.
61. Inegbenosun, H.; Azodo, C.C. Association between Oral Health Literacy, Gingival Health and Oral Hygiene among Dental Patients. *Nigerian Journal of Dental Research* **2020**, *5*.
62. Tsekane, M.; Amone-P'olak, K. *Self-Efficacy as a Predictor of Alcohol Use among Students at a University in Botswana*; 2019; Vol. 18, p. 2019;.
63. Banke-Thomas, A.O.; Kouraogo, S.F.; Siribie, A.; Taddese, H.B.; Mueller, J.E. Knowledge of Obstetric Fistula Prevention amongst Young Women in Urban and Rural Burkina Faso: A Cross-Sectional Study. *PloS one* **2013**, *8*, doi:10.1371/JOURNAL.PONE.0085921.
64. Diendéré, J.; Ouattara, S.; Kaboré, J.; Traoré, I.; Zeba, A.N.; Kouanda, S. Oral Hygiene Practices and Their Sociodemographic Correlates among Adults in Burkina Faso: Results from the First National Survey. *BMC oral health* **2022**, *22*, 86, doi:10.1186/S12903-022-02118-0.
65. Some, M.J.; Ouedraogo, I.; Benedikter, R.; Yameogo, R.; Atemezing, G.; Traoré, I.; Diallo, G. Interactive Voice Response Service to Improve High School Students Covid-19 Literacy in Burkina Faso: A Usability Study. *Studies in health technology and informatics* **2022**, *295*, doi:10.3233/SHTI220763.
66. Moukam, A.M.D.; Owono, M.S.E.; Kenfack, B.; Vassilakos, P.; Petignat, P.; Sormani, J.; Schmidt, N.C. "Cervical Cancer Screening: Awareness Is Not Enough". Understanding Barriers to Screening among Women in West Cameroon-a Qualitative Study Using Focus Groups. *Reproductive health* **2021**, *18*, doi:10.1186/S12978-021-01186-9.
67. Costa, A.; Mourão, S.; Santos, O.; Alarcão, V.; Virgolino, A.; Nogueira, P.; Bettencourt, M.R.; Reis, S.; Graça, A.; Henriques, A. I-DECIDE: A Social Prescribing and Digital Intervention Protocol to Promote Sexual and Reproductive Health and Quality of Life among Young Cape Verdeans. *International journal of environmental research and public health* **2021**, *18*, 1–11, doi:10.3390/IJERPH18030850.
68. Mensah, K.; Assoumou, N.; Duchesne, V.; Pourette, D.; Debeaudrap, P.; Dumont, A. Acceptability of HPV Screening among HIV-Infected Women Attending an HIV-Dedicated Clinic in Abidjan, Côte d'Ivoire. *BMC women's health* **2020**, *20*, doi:10.1186/S12905-020-01021-6.
69. Malamba-Lez, D.; Ngoy-Nkulu, D.; Steels, P.; Tshala-Katumbay, D.; Mullens, W. Heart Failure Etiologies and Challenges to Care in the Developing World: An Observational Study in the Democratic Republic of Congo. *Journal of Cardiac Failure* **2018**, *24*, 854–859, doi:10.1016/j.cardfail.2018.10.008.
70. Stroeken, K.; Remes, P.; De Koker, P.; Michielsen, K.; Van Vossole, A.; Temmerman, M. HIV among Out-of-School Youth in Eastern and Southern Africa: A Review. *AIDS Care* **2012**, *24*, 186–194, doi:10.1080/09540121.2011.596519.

71. Almaleh, R.; Helmy, Y.; Farhat, E.; Hasan, H.; Abdelhafez, A. Assessment of Health Literacy among Outpatient Clinics Attendees at Ain Shams University Hospitals, Egypt: A Cross-Sectional Study. *Public health* **2017**, *151*, 137–145, doi:10.1016/j.puhe.2017.06.024.
72. Alseraty, W.H. Parents' Socioeconomic Status and Health Literacy Domains among Shokrof Preparatory School Students , Shokrof Village, Algarbia Governorate, Egypt. *Journal of Education and Practice* **2015**, *6*, 1–8.
73. Anwar, W.A.; Mostafa, N.S.; Hakim, S.A.; Sos, D.G.; Abozaid, D.A.; Osborne, R.H. Health Literacy Strengths and Limitations among Rural Fishing Communities in Egypt Using the Health Literacy Questionnaire (HLQ). *PloS one* **2020**, *15*, doi:10.1371/JOURNAL.PONE.0235550.
74. Anwar, W.A.; Mostafa, N.S.; Hakim, S.A.; Sos, D.G.; Cheng, C.; Osborne, R.H. Health Literacy Co-Design in a Low Resource Setting: Harnessing Local Wisdom to Inform Interventions across Fishing Villages in Egypt to Improve Health and Equity. *International journal of environmental research and public health* **2021**, *18*, doi:10.3390/IJERPH18094518.
75. McEwan, J.; Underwood, C.; Corbex, M. "Injustice! That Is the Cause": A Qualitative Study of the Social, Economic, and Structural Determinants of Late Diagnosis and Treatment of Breast Cancer in Egypt. *Cancer nursing* **2014**, *37*, 468–475, doi:10.1097/NCC.0000000000000118.
76. Mostafa, A.; Abdelzaher, A.; Rashed, S.; Alkhawaga, S.I.; Afifi, S.K.; Abdelalim, S.; Mostafa, S.A.; Zidan, T.A. Is Health Literacy Associated with Antibiotic Use, Knowledge and Awareness of Antimicrobial Resistance among Non-Medical University Students in Egypt? A Cross-Sectional Study. *BMJ open* **2021**, *11*, doi:10.1136/BMJOPEN-2020-046453.
77. Wahba, M.S.; Abdel-Gawwad, E.S.; El-Bourgy, M.D.; Abdel-Kader, H.Z.; Abou-Zeid, A.A. Consumer-Oriented Evaluation of the Service Provided by the Department of Health Education and Information in Alexandria, Egypt. *The Journal of the Egyptian Public Health Association* **2017**, *92*, 116–127, doi:10.21608/EPX.2018.8949.
78. Ngwenya, T.Z.; Huang, N.; Wang, I.A.; Chen, C.Y. Urban-Rural Differences in Depression Literacy Among High School Teachers in the Kingdom of Eswatini. *Journal of School Health* **2022**, *92*, 561–569, doi:10.1111/josh.13173.
79. Asamrew, N.; Endris, A.A.; Tadesse, M. Level of Patient Satisfaction with Inpatient Services and Its Determinants: A Study of a Specialized Hospital in Ethiopia. *Journal of environmental and public health* **2020**, *2020*, doi:10.1155/2020/2473469.
80. Asemahagn, M.A. Sputum Smear Conversion and Associated Factors among Smear-Positive Pulmonary Tuberculosis Patients in East Gojjam Zone, Northwest Ethiopia: A Longitudinal Study. *BMC pulmonary medicine* **2021**, *21*, doi:10.1186/S12890-021-01483-W.
81. Asemahagn, M.A.; Alene, G.D.; Yimer, S.A. A Qualitative Insight into Barriers to Tuberculosis Case Detection in East Gojjam Zone, Ethiopia. *The American journal of tropical medicine and hygiene* **2020**, *103*, 1455–1465, doi:10.4269/AJTMH.20-0050.
82. Avan, B.I.; Berhanu, D.; Mekonnen, Y.; Beaumont, E.; Tomlin, K.; Allen, E.; Schellenberg, J. Embedding Community-Based Newborn Care in the Ethiopian Health System: Lessons from a 4-Year Programme Evaluation. *Health policy and planning* **2021**, *36*, I22–I32, doi:10.1093/HEAPOL/CZAB085.
83. Ayele, B.G.; Woldu, M.A.; Gebrehiwot, H.W.; Gebre-Egziabher, E.G.; Gebretnsae, H.; Hadgu, T.; Abrha, A.A.; Medhanyie, A.A. Magnitude and Determinants for Place of Postnatal Care Utilization among Mothers Who Delivered at Home in Ethiopia: A Multinomial Analysis from the 2016 Ethiopian Demographic Health Survey. *Reproductive health* **2019**, *16*, doi:10.1186/S12978-019-0818-2.
84. Ayode, D.; McBride, C.M.; Heer, H.D.; Watanabe, E.; Gebreyesus, T.; Tadele, G.; Tora, A.; Davey, G. The Association of Beliefs about Heredity with Preventive and Interpersonal Behaviors in Communities Affected by Podoconiosis in Rural Ethiopia. *The American journal of tropical medicine and hygiene* **2012**, *87*, 623–630, doi:10.4269/AJTMH.2012.12-0204.
85. Chereka, A.A.; Demsash, A.W.; Ngusie, H.S.; Kassie, S.Y. Digital Health Literacy to Share COVID-19 Related Information and Associated Factors among Healthcare Providers Worked at COVID-19 Treatment Centers in Amhara Region, Ethiopia: A Cross-Sectional Survey. *Informatics in medicine unlocked* **2022**, *30*, doi:10.1016/J.IMU.2022.100934.
86. Dugasa, Y.G. Level of Patient Health Literacy and Associated Factors Among Adult Admitted Patients at Public Hospitals of West Shoa Oromia, Ethiopia. *Patient preference and adherence* **2022**, *16*, 853–859, doi:10.2147/PPA.S357741.
87. Gedefaw, A.; Yilma, T.M.; Endehabtu, B.F. Information Seeking Behavior About Cancer and Associated Factors Among University Students, Ethiopia: A Cross-Sectional Study. *Cancer management and research* **2020**, *12*, 4829–4839, doi:10.2147/CMAR.S259849.
88. Gonete, A.T.; Alemu, T.G.; Mekonnen, E.G.; Takele, W.W. Malnutrition and Contributing Factors among Newborns Delivered at the University of Gondar Hospital, Northwest Ethiopia: A Cross-Sectional Study. *BMJ open* **2021**, *11*, doi:10.1136/BMJOPEN-2021-053577.
89. Gurmu, Y.; Gela, D.; Aga, F. Factors Associated with Self-Care Practice among Adult Diabetes Patients in West Shoa Zone, Oromia Regional State, Ethiopia. *BMC health services research* **2018**, *18*, doi:10.1186/S12913-018-3448-4.
90. Kassahun, T.; Gesesew, H.; Mwanri, L.; Eshetie, T. Diabetes Related Knowledge, Self-Care Behaviours and Adherence to Medications among Diabetic Patients in Southwest Ethiopia: A Cross-Sectional Survey. *BMC endocrine disorders* **2016**, *16*, doi:10.1186/S12902-016-0114-X.
91. Kassie, S.Y.; Melese, T.; Handebo, S.; Sebastian, Y.; Ngusie, H.S. Information Seeking about COVID-19 and Associated Factors among Chronic Patients in Bahir Dar City Public Hospitals, Northwest Ethiopia: A Cross-Sectional Study. *BMC infectious diseases* **2022**, *22*, doi:10.1186/S12879-022-07315-4.
92. Kebede, A.; Wabe, N.T. Medication Adherence and Its Determinants among Patients on Concomitant Tuberculosis and Antiretroviral Therapy in South West Ethiopia. *North American Journal of Medical Sciences* **2012**, *4*, 67–71, doi:10.4103/1947-2714.93376.
93. Menberu, M.; Mekonen, T.; Azale, T.; Ayano, G.; Yimer, S.; Getnet, A.; Belete, A.; Kerie, S.; Fekadu, W. Health Care Seeking Behavior for Depression in Northeast Ethiopia: Depression Is Not Considered as Illness by More than Half of the Participants. *Annals of general psychiatry* **2018**, *17*, doi:10.1186/S12991-018-0205-3.
94. Shiferaw, K.B.; Tilahun, B.C.; Endehabtu, B.F.; Gullslett, M.K.; Mengiste, S.A. E-Health Literacy and Associated Factors among Chronic Patients in a Low-Income Country: A Cross-Sectional Survey. *BMC medical informatics and decision making* **2020**, *20*, doi:10.1186/S12911-020-01202-1.

95. Posso, A.; Perera, U.D.S.; Mishra, A. Community-Level Health Programs and Child Labor: Evidence from Ethiopia. *Health economics* **2021**, *30*, 2995–3015, doi:10.1002/HEC.4429.
96. Shahvisi, A.; Meskele, E.; Davey, G. A Human Right to Shoes? Establishing Rights and Duties in the Prevention and Treatment of Podoconiosis. *Health and human rights* **2018**, *20*, 53–65.
97. Tefera, Y.G.; Gebresillassie, B.M.; Emiru, Y.K.; Yilma, R.; Hafiz, F.; Akalu, H.; Ayele, A.A. Diabetic Health Literacy and Its Association with Glycemic Control among Adult Patients with Type 2 Diabetes Mellitus Attending the Outpatient Clinic of a University Hospital in Ethiopia. *PloS one* **2020**, *15*, doi:10.1371/JOURNAL.PONE.0231291.
98. Teklu, A.M.; Abraha, M.; Legesse, T.; Bekele, M.; Getachew, A.; Aseffa, B.; Molla, M.; Belachew, F.; Haregu, T.N. Awareness of Diagnosis, Treatment Plan and Prognosis among Patients Attending Public Hospitals and Health Centers in Addis Ababa, Ethiopia. *PloS one* **2022**, *17*, e0270397, doi:10.1371/JOURNAL.PONE.0270397.
99. Tesfaye, Y.; Agenagnew, L.; Anand, S.; Tucho, G.T.; Birhanu, Z.; Ahmed, G.; Getnet, M.; Yitbarek, K. Knowledge of the Community Regarding Mental Health Problems: A Cross-Sectional Study. *BMC psychology* **2021**, *9*, doi:10.1186/S40359-021-00607-5.
100. Tilahun, D.; Abera, A.; Nemera, G. Communicative Health Literacy in Patients with Non-Communicable Diseases in Ethiopia: A Cross-Sectional Study. *Trop Med Health* **2021**, *49*, 57, doi:10.1186/s41182-021-00345-9.
101. Tilahun, D.; Gezahegn, A.; Tegenu, K.; Fenta, B. Functional Health Literacy in Patients with Cardiovascular Diseases: Cross-Sectional Study in Ethiopia. *International journal of general medicine* **2021**, *14*, 1967–1974, doi:10.2147/IJGM.S304007.
102. Tora, A.; Tadele, G.; Aseffa, A.; McBride, C.M.; Davey, G. Health Beliefs of School-Age Rural Children in Podoconiosis-Affected Families: A Qualitative Study in Southern Ethiopia. *PLoS neglected tropical diseases* **2017**, *11*, doi:10.1371/JOURNAL.PNTD.0005564.
103. Adu, P.; Jurcik, T.; Dmitry, G. Mental Health Literacy in Ghana: Implications for Religiosity, Education and Stigmatization. *Transcultural Psychiatry* **2021**, *58*, 516–531, doi:10.1177/13634615211022177.
104. Amoah, P.A. Social Participation, Health Literacy, and Health and Well-Being: A Cross-Sectional Study in Ghana. *SSM - population health* **2018**, *4*, 263–270, doi:10.1016/J.SSMPH.2018.02.005.
105. Amoah, P.A. The Relationship among Functional Health Literacy, Self-Rated Health, and Social Support among Younger and Older Adults in Ghana. *International journal of environmental research and public health* **2019**, *16*, doi:10.3390/IJERPH16173188.
106. Amoah, P.A.; Phillips, D.R. Health Literacy and Health: Rethinking the Strategies for Universal Health Coverage in Ghana. *Public health* **2018**, *159*, 40–49, doi:10.1016/J.PUHE.2018.03.002.
107. Amoah, P.A.; Phillips, D.R. Socio-Demographic and Behavioral Correlates of Health Literacy: A Gender Perspective in Ghana. *Women & health* **2020**, *60*, 123–139, doi:10.1080/03630242.2019.1613471.
108. Amoah, P.A.; Musalia, J.; Busia, K.A. Health Behaviors and Health Literacy: Questing the Role of Weak Social Ties Among Older Persons in Rural and Urban Ghana. *Frontiers in Public Health* **2022**, *10*, doi:10.3389/fpubh.2022.777217.
109. Amoah, P.A.; Koduah, A.O.; Gyasi, R.M.; Nyamekye, K.A.; Phillips, D.R. Association of Health Literacy and Socioeconomic Status with Oral Health Among Older Adults in Ghana: A Moderation Analysis of Social Capital. *Journal of applied gerontology : the official journal of the Southern Gerontological Society* **2022**, *41*, 671–679, doi:10.1177/07334648211028391.
110. Arthur, Y.A.; Boardman, G.H.; Morgan, A.J.; McCann, T.V. Effectiveness of a Problem-Solving, Story-Bridge Mental Health Literacy Programme in Improving Ghanaian Community Leaders' Attitudes towards People with Mental Illness: A Cluster Randomised Controlled Trial. *Issues in mental health nursing* **2021**, *42*, 332–345, doi:10.1080/01612840.2020.1799273.
111. Arthur, Y.A.; Boardman, G.H.; Morgan, A.J.; McCann, T.V. Cluster Randomised Controlled Trial of a Problem-Solving, Story-Bridge Mental Health Literacy Programme for Improving Ghanaian Community Leaders' Knowledge of Depression. *Journal of Mental Health* **2020**, doi:10.1080/09638237.2020.1793122.
112. Arthur, Y.A.; Boardman, G.H.; McCann, T.V. Qualitative Process Evaluation of a Problem-Solving and Story-Bridge Based Mental Health Literacy Program with Community Leaders in Ghana. *International Journal of Mental Health Nursing* **2021**, *30*, 683–693, doi:10.1111/inm.12832.
113. Arthur, Y.A. *Evaluation of a Mental Health Literacy Programme on Community Leaders' Knowledge about and Attitude towards People with Mental Disorders in Ghana: Cluster Randomised Controlled Trial*; 2018;
114. Boateng, M.A.; Agyei-Baffour, P.; Angel, S.; Enemark, U. Translation, Cultural Adaptation and Psychometric Properties of the Ghanaian Language (Akan; Asante Twi) Version of the Health Literacy Questionnaire. *BMC health services research* **2020**, *20*, doi:10.1186/S12913-020-05932-W.
115. Boateng, M.A.; Agyei-Baffour, E.; Angel, S.; Asare, O.; Prempeh, B.; Enemark, U. Co-Creation and Prototyping of an Intervention Focusing on Health Literacy in Management of Malaria at Community-Level in Ghana. *Research involvement and engagement* **2021**, *7*, doi:10.1186/S40900-021-00302-0.
116. Evans, A.-Y.; Anthony, E.; Gabriel, G. Comprehensive Health Literacy Among Undergraduates: A Ghanaian University-Based Cross-Sectional Study. *Health literacy research and practice* **2019**, *3*, doi:10.3928/24748307-20190903-01.
117. Gupta, S.; Tutu, R.A.; Boateng, J.; Busingye, J.D.; Elavarthi, S. Self-Reported Functional, Communicative, and Critical Health Literacy on Foodborne Diseases in Accra, Ghana. *Tropical medicine and health* **2018**, *46*, doi:10.1186/S41182-018-0097-6.
118. Koduah, A.O.; Leung, A.Y.M.; Leung, D.Y.L.; Liu, J.Y.W. "I Sometimes Ask Patients to Consider Spiritual Care": Health Literacy and Culture in Mental Health Nursing Practice. *International journal of environmental research and public health* **2019**, *16*, doi:10.3390/IJERPH16193589.
119. Koduah, A.O.; Amoah, P.A.; Nkansah, J.O.; Leung, A.Y.M. A Comparative Analysis of Student and Practising Nurses' Health Literacy Knowledge in Ghana. *Healthcare (Basel, Switzerland)* **2021**, *9*, doi:10.3390/HEALTHCARE9010038.
120. Kpobi, L.; Swartz, L. Explanatory Models of Mental Disorders among Traditional and Faith Healers in Ghana. *International Journal of Culture and Mental Health* **2018**, 1–11, doi:10.1080/17542863.2018.1468473.
121. Kugbey, N.; Meyer-Weitz, A.; Oppong Asante, K. Access to Health Information, Health Literacy and Health-Related Quality of Life among Women Living with Breast Cancer: Depression and Anxiety as Mediators. *Patient Education and Counseling* **2019**, *102*, 1357–1363, doi:10.1016/j.pec.2019.02.014.
122. Lori, J.R.; Munro, M.L.; Chuey, M.R. Use of a Facilitated Discussion Model for Antenatal Care to Improve Communication. *International Journal of Nursing Studies* **2016**, *54*, 84–94, doi:10.1016/j.ijnurstu.2015.03.018.

123. Lori, J.R.; Ofosu-Darkwah, H.; Boyd, C.J.; Banerjee, T.; Adanu, R.M.K. Improving Health Literacy through Group Antenatal Care: A Prospective Cohort Study. *BMC pregnancy and childbirth* **2017**, *17*, doi:10.1186/S12884-017-1414-5.
124. Lori, J.R.; Dahlem, C.H.Y.; Ackah, J.V.; Adanu, R.M.K. Examining Antenatal Health Literacy in Ghana. *Journal of Nursing Scholarship* **2014**, *46*, 432–440, doi:10.1111/jnu.12094.
125. Nangsangna, R.D.; Vroom, F.D.-C. Factors Influencing Online Health Information Seeking Behavior among Patients in Kwahu West Municipal, Nkawkaw, Ghana. *Online Journal of Public Health Informatics* **2019**, *11*, doi:10.5210/ojphi.v11i2.10141.
126. Seneadza, N.A.H.; Insaidoo, G.; Boye, H.; Ani-Amponsah, M.; Leung, T.; Meek, J.; Enweronu-Laryea, C. Neonatal Jaundice in Ghanaian Children: Assessing Maternal Knowledge, Attitude, and Perceptions. *PloS one* **2022**, *17*, doi:10.1371/JOURNAL.PONE.0264694.
127. Tutu, R.A.; Gupta, S.; Elavarthi, S.; Busingye, J.D.; Boateng, J.K. Exploring the Development of a Household Cholera-Focused Health Literacy Scale in James Town, Accra. *Journal of infection and public health* **2019**, *12*, 62–69, doi:10.1016/J.JIPH.2018.08.006.
128. Tutu, R.A.; Gupta, S.; Busingye, J.D. Examining Health Literacy on Cholera in an Endemic Community in Accra, Ghana: A Cross-Sectional Study. *Tropical medicine and health* **2019**, *47*, doi:10.1186/S41182-019-0157-6.
129. Darteh, E.K.M.; Dickson, K.S.; Amu, H. Understanding the Socio-Demographic Factors Surrounding Young Peoples' Risky Sexual Behaviour in Ghana and Kenya. *Journal of Community Health* **2020**, *45*, 141–147, doi:10.1007/s10900-019-00726-6.
130. McGinn, T.; Allen, K. Improving Refugees' Reproductive Health through Literacy in Guinea. *Global public health* **2006**, *1*, 229–248, doi:10.1080/17441690600680002.
131. Dyrehave, C.; Rasmussen, D.N.; Hønge, B.L.; Jespersen, S.; Correia, F.G.; Medina, C.; Wejse, C.; Rodkjaer, L. Nonadherence Is Associated with Lack of HIV-Related Knowledge: A Cross-Sectional Study among HIV-Infected Individuals in Guinea-Bissau. *Journal of the International Association of Providers of AIDS Care* **2016**, *15*, 350–358, doi:10.1177/2325957415599211.
132. Abajobir, A.; Groot, R. de; Wainaina, C.; Njeri, A.; Maina, D.; Njoki, S.; Mbaya, N.; Donfouet, H.P.P.; Pradhan, M.; Janssens, W.; et al. The Impact of I-PUSH on Maternal and Child Health Care Utilization, Health Outcomes, and Financial Protection: Study Protocol for a Cluster Randomized Controlled Trial Based on Financial and Health Diaries Data. *Trials* **2021**, *22*, doi:10.1186/S13063-021-05598-7.
133. Kassaman, D.; Mushani, T.; Kiraithe, P.; Brownie, S.; Barton-Burke, M. Fear, Faith and Finances: Health Literacy Experiences of English and Swahili Speaking Women Newly Diagnosed with Breast and Cervical Cancer. *Ecancermedicalscience* **2022**, *16*, 1350, doi:10.3332/ecancer.2022.1350.
134. Khares, I.B.; Nduati, R.; Khares, I.B.; Laigong, P. *QUALITY OF CARE PROVIDED TO ADOLESCENTS AGED BETWEEN 10 AND 19 YEARS IN KENYATTA NATIONAL HOSPITAL*; 2018; Vol. 95;.
135. Marangu, E.; Mansouri, F.; Sands, N.; Ndetei, D.; Muriithi, P.; Wynter, K.; Rawson, H. Assessing Mental Health Literacy of Primary Health Care Workers in Kenya: A Cross-Sectional Survey. *International journal of mental health systems* **2021**, *15*, doi:10.1186/S13033-021-00481-Z.
136. McMahon, D.E.; Singh, R.; Chemtai, L.; Semeere, A.; Byakwaga, H.; Grant, M.; Laker-Oketta, M.; Lagat, C.; Collier, S.; Maurer, T.; et al. Barriers and Facilitators to Chemotherapy Initiation and Adherence for Patients with HIV-Associated Kaposi's Sarcoma in Kenya: A Qualitative Study. *Infect Agent Cancer* **2022**, *17*, 37, doi:10.1186/s13027-022-00444-0.
137. Muga, T.; Mbuthia, G.; Gatimu, S.M.; Rossiter, R. A Pilot Study Exploring Nursing Knowledge of Depression and Suicidal Ideation in Kenya. *Issues in mental health nursing* **2019**, *40*, 15–20, doi:10.1080/01612840.2018.1489922.
138. Mutiso, V.N.; Pike, K.M.; Musyimi, C.N.; Rebello, T.J.; Tele, A.; Gitonga, I.; Thornicroft, G.; Ndetei, D.M. Changing Patterns of Mental Health Knowledge in Rural Kenya after Intervention Using the WHO mhGAP-Intervention Guide. *Psychological medicine* **2019**, *49*, 2227–2236, doi:10.1017/S0033291718003112.
139. Mwaisaka, J.; Gonsalves, L.; Thiongo, M.; Waithaka, M.; Sidha, H.; Alfred, O.; Mukiira, C.; Gichangi, P. Young People's Experiences Using an On-Demand Mobile Health Sexual and Reproductive Health Text Message Intervention in Kenya: Qualitative Study. *JMIR mHealth and uHealth* **2021**, *9*, e19109, doi:10.2196/19109.
140. Mwiti, B.K.; Ambole, A.; Osanjo, L. 'Bottom up' Approach: A Community-Based Intervention in Fighting Non-Communicable Diseases in Urban Informal Settlements Kenya. *East African Medical Journal* **2019**, *95*.
141. Raufman, J.; Blansky, D.; Lounsbury, D.W.; Mwangi, E.W.; Lan, Q.; Olloquequi, J.; Hosgood, H.D. Environmental Health Literacy and Household Air Pollution-Associated Symptoms in Kenya: A Cross-Sectional Study. *Environmental health : a global access science source* **2020**, *19*, doi:10.1186/S12940-020-00643-5.
142. Sripad, P.; Merritt, M.W.; Kerrigan, D.; Abuya, T.; Ndwiga, C.; Warren, C.E. Determining a Trusting Environment for Maternity Care: A Framework Based on Perspectives of Women, Communities, Service Providers, and Managers in Peri-Urban Kenya. *Frontiers in global women's health* **2022**, *3*, 818062, doi:10.3389/fgwh.2022.818062.
143. Mugomeri, E.; Ramathebane, M.V.; Maja, L.; Chatanga, P.; Moletsane, L. Knowledge of Disease Condition and Medications among Hypertension Patients in Lesotho. *Journal of the American Society of Hypertension* **2016**, *10*, 41–46, doi:10.1016/j.jash.2015.10.009.
144. Reid, M.; Nel, M.; Rensburg-Bonthuyzen, E.J. van Development of a Sesotho Health Literacy Test in a South African Context. *African journal of primary health care & family medicine* **2019**, *11*, doi:10.4102/PHCFM.V11I1.1853.
145. Brick, K.; Cooper, J.L.; Mason, L.; Faeflen, S.; Monmia, J.; Dubinsky, J.M. Training-of-Trainers Neuroscience and Mental Health Teacher Education in Liberia Improves Self-Reported Support for Students. *Frontiers in Human Neuroscience* **2021**, *15*, doi:10.3389/fnhum.2021.653069.
146. Asgary, R.; Liu, M.; Naderi, R.; Grigoryan, Z.; Malachovsky, M. Malnutrition Prevalence and Nutrition Barriers in Children under 5 Years: A Mixed Methods Study in Madagascar. *International Health* **2015**, *7*, 426–432, doi:10.1093/inthealth/ihv016.
147. David Rakotonandrasana Harimbola, M., PhD; Kaori Mizumoto, P. Individual and Household Risk Factors for Severe Acute Malnutrition among Under-Five Children in the Analamanga Region, Madagascar. *International journal of MCH and AIDS* **2018**, *7*, 38–46, doi:10.21106/IJMA.248.

148. Jumbe, S.; Nyali, J.; Simbeye, M.; Zakeyu, N.; Motshewa, G.; Pulapa, S.R. “We Do Not Talk about It”: Engaging Youth in Malawi to Inform Adaptation of a Mental Health Literacy Intervention. *PloS one* **2022**, *17*, doi:10.1371/JOURNAL.PONE.0265530.
149. Kalanda, B.F.; Buuren, S.V.; Verhoeff, F.H.; Brabin, B.J. Catch-up Growth in Malawian Babies, a Longitudinal Study of Normal and Low Birthweight Babies Born in a Malarious Endemic Area. *Early human development* **2005**, *81*, 841–850, doi:10.1016/J.EARLHUMDEV.2005.06.006.
150. Kohler, I.V.; Bandawe, C.; Ciancio, A.; Kämpfen, F.; Payne, C.F.; Mwera, J.; Mkandawire, J.; Kohler, H.-P. Cohort Profile: The Mature Adults Cohort of the Malawi Longitudinal Study of Families and Health (MLSFH-MAC). *BMJ Open* **2020**, *10*, e038232, doi:10.1136/bmjopen-2020-038232.
151. Kululanga, L.I.; Kadango, A.; Lungu, G.; Jere, D.; Ngwale, M.; Kumbani, L.C. Knowledge Deficit on Health Promotion Activities during Pregnancy: The Case for Adolescent Pregnant Women at Chiladzulu District, Malawi. *BMC pregnancy and childbirth* **2020**, *20*, doi:10.1186/S12884-020-03386-W.
152. Kutcher, S.; Udedi, M.; Gilberds, H.; Brown, A.; Chapota, R.; Perkins, K. Clinic Outcomes of the Pathway to Care Model: A Cross-Sectional Survey of Adolescent Depression in Malawi. *Malawi medical journal : the journal of Medical Association of Malawi* **2017**, *29*, 97–102, doi:10.4314/MMJ.V29I2.4.
153. Kutcher, S.; Gilberds, H.; Morgan, C.; Greene, R.; Hamwaka, K.; Perkins, K. Improving Malawian Teachers’ Mental Health Knowledge and Attitudes: An Integrated School Mental Health Literacy Approach. *Global mental health (Cambridge, England)* **2015**, *2*, doi:10.1017/GMH.2014.8.
154. Ogunrinu, T.; Gamboa-Maldonado, T.; Ngewa, R.N.; Saunders, J.; Crounse, J.; Misiri, J. A Qualitative Study of Health Education Experiences and Self-Management Practices among Patients with Type 2 Diabetes at Malamulo Adventist Hospital in Thyolo District, Malawi. *Malawi medical journal : the journal of Medical Association of Malawi* **2017**, *29*, 118–123, doi:10.4314/MMJ.V29I2.8.
155. Patel, P.; Adebisi, Y.A.; Steven, M.; Lucero-Prisno, D.E. Addressing COVID-19 in Malawi. *The Pan African medical journal* **2020**, *35*, 1–2, doi:10.11604/PAMJ.SUPP.2020.35.2.23960.
156. Tilly, A.E.; Ellis, G.K.; Chen, J.S.; Manda, A.; Salima, A.; Mtangwanika, A.; Tewete, B.; Kaimila, B.; Kasonkanji, E.; Kayira, E.; et al. Implementation and Evaluation of Educational Videos to Improve Cancer Knowledge and Patient Empowerment. *JCO global oncology* **2022**, *8*, doi:10.1200/GO.21.00315.
157. Uwamahoro, N.S.; Ngwira, B.; Vinther-Jensen, K.; Rowlands, G. Health Literacy among Malawian HIV-Positive Youth: A Qualitative Needs Assessment and Conceptualization. *Health promotion international* **2020**, *35*, 1137–1149, doi:10.1093/HEAPRO/DAZ107.
158. Ménard, S.; Jbilou, J.; Lauzier, S. Family Caregivers’ Reported Nonadherence to the Controller Medication of Asthma in Children in Casablanca (Morocco): Extent and Associated Factors. *Journal of Asthma* **2018**, *55*, 1362–1372, doi:10.1080/02770903.2017.1414235.
159. Park, S.; Moon, N.; Oh, B.; Park, M.; Kang, K.; Sentissi, I.; Bae, S.H. Improving Treatment Adherence with Integrated Patient Management for TB Patients in Morocco. *International journal of environmental research and public health* **2021**, *18*, doi:10.3390/IJERPH18199991.
160. Howard, L.M.; Tique, J.A.; Gaveta, S.; Sidat, M.; Rothman, R.L.; Vermund, S.H.; Ciampa, P.J. Health Literacy Predicts Pediatric Dosing Accuracy for Liquid Zidovudine. *AIDS (London, England)* **2014**, *28*, 1041–1048, doi:10.1097/QAD.0000000000000197.
161. Lindberg, L.; Nhambongo, I.; Nhampossa, T.; Munguambe, K.; Priebe, G. A Qualitative Study of Mothers’ Health Literacy Related to Malnutrition in under 5-Year-Old Children in Southern Mozambique. *Public health nutrition* **2021**, doi:10.1017/S1368980021004365.
162. Tique, J.A.; Howard, L.M.; Gaveta, S.; Sidat, M.; Rothman, R.L.; Vermund, S.H.; Ciampa, P.J. Measuring Health Literacy Among Adults with HIV Infection in Mozambique: Development and Validation of the HIV Literacy Test. *AIDS and behavior* **2017**, *21*, 822–832, doi:10.1007/S10461-016-1348-3.
163. Nair, M.; Baltag, V.; Bose, K.; Boschi-Pinto, C.; Lambrechts, T.; Mathai, M. Improving the Quality of Health Care Services for Adolescents, Globally: A Standards-Driven Approach. *The Journal of adolescent health : official publication of the Society for Adolescent Medicine* **2015**, *57*, 288–298, doi:10.1016/J.JADOHEALTH.2015.05.011.
164. Theron, G.; Peter, J.; Zijenah, L.; Chanda, D.; Mangu, C.; Clowes, P.; Rachow, A.; Lesosky, M.; Hoelscher, M.; Pym, A.; et al. Psychological Distress and Its Relationship with Non-Adherence to TB Treatment: A Multicentre Study. *BMC infectious diseases* **2015**, *15*, doi:10.1186/S12879-015-0964-2.
165. Hanlon, C.; Luitel, N.P.; Kathree, T.; Murhar, V.; Shrivasta, S.; Medhin, G.; Ssebunnya, J.; Fekadu, A.; Shidhaye, R.; Petersen, I.; et al. Challenges and Opportunities for Implementing Integrated Mental Health Care: A District Level Situation Analysis from Five Low- and Middle-Income Countries. *PLoS ONE* **2014**, *9*, doi:10.1371/journal.pone.0088437.
166. Ramazanu, S.; Bautista, C.; Green, T.; Rhudy, L.M.; Rogado, M.I.C.; Baby, P.; Woon, C.; Martinez, R.C.K.P.; Gaudecker, J.R. von; Nydahl, P.; et al. Challenges and Opportunities in Stroke Nursing Research: Global Views From a Panel of Nurse Researchers. *The Journal of neuroscience nursing : journal of the American Association of Neuroscience Nurses* **2022**, *54*, 111–115, doi:10.1097/JNN.0000000000000643.
167. Grady, M.; Venugopal, U.; Robert, K.; Hurrell, G.; Schnell, O. Health Care Professionals’ Clinical Perspectives and Acceptance of a Blood Glucose Meter and Mobile App Featuring a Dynamic Color Range Indicator and Blood Sugar Mentor: Online Evaluation in Seven Countries. *JMIR Human Factors* **2019**, *6*, e13847, doi:10.2196/13847.
168. Aung, M.N.; Stein, C.; Chen, W.-T.; Garg, V.; Sitepu, M.S.; Thu, N.T.D.; Gundran, C.P.D.; Hassan, M.R.; Suthutvoravut, U.; Soe, A.N.; et al. Community Responses to COVID-19 Pandemic First Wave Containment Measures: A Multinational Study. *The Journal of Infection in Developing Countries* **2021**, *15*, 1107–1116, doi:10.3855/jidc.15254.
169. Robertson, N.M.; Siddharthan, T.; Pollard, S.L.; Alupo, P.; Flores-Flores, O.; Rykiel, N.A.; Romani, E.D.; Ascencio-Días, I.; Kirenga, B.; Checkley, W.; et al. Development and Validity Assessment of a Chronic Obstructive Pulmonary Disease Knowledge Questionnaire in Low- and Middle-Income Countries. *Annals of the American Thoracic Society* **2021**, *18*, 1298–1305, doi:10.1513/AnnalsATS.202007-884OC.
170. Pleasant, A.; Kuruvilla, S. A Tale of Two Health Literacies: Public Health and Clinical Approaches to Health Literacy. *Health promotion international* **2008**, *23*, 152–159, doi:10.1093/heapro/dan001.
171. Mogobe, K.D.; Shaibu, S.; Matshediso, E.; Sabone, M.; Ntsayagae, E.; Nicholas, P.K.; Portillo, C.J.; Corless, I.B.; Rose, C.D.; Johnson, M.O.; et al. Language and Culture in Health Literacy for People Living with HIV: Perspectives of Health Care Providers and Professional Care Team Members. *AIDS research and treatment* **2016**, *2016*, doi:10.1155/2016/5015707.
172. McClintock, H.F.; Alber, J.M.; Schrauben, S.J.; Mazzola, C.M.; Wiebe, D.J. Constructing a Measure of Health Literacy in Sub-Saharan African Countries. *Health Promotion International* **2020**, *35*, 907–915, doi:10.1093/heapro/daz078.

173. Nachega, J.B.; Morroni, C.; Zuniga, J.M.; Schechter, M.; Rockstroh, J.; Solomon, S.; Sherer, R. HIV Treatment Adherence, Patient Health Literacy, and Health Care Provider-Patient Communication: Results from the 2010 AIDS Treatment for Life International Survey. *Journal of the International Association of Physicians in AIDS Care* **2012**, *11*, 128–133, doi:10.1177/1545109712437244.
174. Popoola, B.O. Involving Libraries in Improving Health Literacy to Achieve Sustainable Development Goal-3 in Developing Economies: A Literature Review. *Health information and libraries journal* **2019**, *36*, 111–120, doi:10.1111/HIR.12255.
175. Weist, M.D.; Bruns, E.J.; Whitaker, K.; Wei, Y.; Kutcher, S.; Larsen, T.; Holsen, I.; Cooper, J.L.; Geroski, A.; Short, K.H. School Mental Health Promotion and Intervention: Experiences from Four Nations. *School Psychology International* **2017**, *38*, 343–362, doi:10.1177/0143034317695379.
176. Bowser, D.; Marqusee, H.; Koussa, M.E.; Atun, R. Health System Barriers and Enablers to Early Access to Breast Cancer Screening, Detection, and Diagnosis: A Global Analysis Applied to the MENA Region. *Public health* **2017**, *152*, 58–74, doi:10.1016/J.PUHE.2017.07.020.
177. Kutcher, S.; Perkins, K.; Gilberds, H.; Udedi, M.; Ubuguyu, O.; Njau, T.; Chapota, R.; Hashish, M. Creating Evidence-Based Youth Mental Health Policy in Sub-Saharan Africa: A Description of the Integrated Approach to Addressing the Issue of Youth Depression in Malawi and Tanzania. *Frontiers in psychiatry* **2019**, *10*, doi:10.3389/FPSYT.2019.00542.
178. Hirvonen, N.; Enwald, H.; Mayer, A.K.; Korpelainen, R.; Pyky, R.; Salonurmi, T.; Savolainen, M.J.; Nengomasha, C.; Abankwah, R.; Uutoni, W.; et al. Screening Everyday Health Information Literacy among Four Populations. *Health information and libraries journal* **2020**, *37*, 192–203, doi:10.1111/HIR.12304.
179. Pavarini, G.; Booysen, C.; Jain, T.; Lai, J.; Manku, K.; Foster-Estwick, A.; Gatera, G.; Juma, D.O.; Karorero, D.; Philip-Joe, K.; et al. Agents of Change for Mental Health: A Survey of Young People's Aspirations for Participation Across Five Low- and Middle-Income Countries. *The Journal of adolescent health : official publication of the Society for Adolescent Medicine* **2022**, doi:10.1016/j.jadohealth.2021.10.037.
180. Seytre, B.; Barros, C.; Bona, P.; Fall, B.; Konate, B.; Rodrigues, A.; Varela, O.; Yoro, M.B. Revisiting COVID-19 Communication in Western Africa: A Health Literacy-Based Approach to Health Communication. *The American journal of tropical medicine and hygiene* **2021**, *105*, 708–712, doi:10.4269/AJTMH.21-0013.
181. Al-Rousan, T.; Pesantes, M.A.; Dadabhai, S.; Kandula, N.R.; Huffman, M.D.; Miranda, J.J.; Vidal-Perez, R.; Dzudie, A.; Anderson, C.A.M. Patients' Perceptions of Self-Management of High Blood Pressure in Three Low- and Middle-Income Countries: Findings from the BPMONITOR Study. *Global health, epidemiology and genomics* **2020**, *5*, doi:10.1017/GHEG.2020.5.
182. louyeh, A.A.; Takian, A.; Ahmadi, B.; Arab, M.; Kiakalayeh, A.D. Patterns of the Social Approach to Health in Selected Countries and Iran: A Comparative Study. *Medical journal of the Islamic Republic of Iran* **2021**, *35*, 1–11, doi:10.47176/MJIRI.35.171.
183. Velden, A.W. van der; Sessa, A.; Altiner, A.; Pignatari, A.C.C.; Shephard, A. Patients with Sore Throat: A Survey of Self-Management and Healthcare-Seeking Behavior in 13 Countries Worldwide. *Pragmatic and observational research* **2020**, *11*, 91–102, doi:10.2147/POR.S255872.
184. Austvoll-Dahlgren, A.; Guttersrud, Ø.; Nsangi, A.; Semakula, D.; Oxman, A.D. Measuring Ability to Assess Claims about Treatment Effects: A Latent Trait Analysis of Items from the "Claim Evaluation Tools" Database Using Rasch Modelling. *BMJ Open* **2017**, *7*, doi:10.1136/bmjopen-2016-013185.
185. Korhonen, J.; Axelin, A.; Katajisto, J.; Lahti, M. Construct Validity and Internal Consistency of the Revised Mental Health Literacy Scale in South African and Zambian Contexts. *Nursing open* **2022**, *9*, doi:10.1002/NOP2.1132.
186. Lahti, M.; Groen, G.; Mwape, L.; Korhonen, J.; Breet, E.; Chapima, F.; Coetzee, M.; Ellilä, H.; Jansen, R.; Jonker, D.; et al. Design and Development Process of a Youth Depression Screening M-Health Application for Primary Health Care Workers in South Africa and Zambia: An Overview of the MEGA Project. *Issues in Mental Health Nursing* **2020**, *41*, 24–30, doi:10.1080/01612840.2019.1604919.
187. Kagee, A.; Remien, R.H.; Berkman, A.; Hoffman, S.; Campos, L.; Swartz, L. Structural Barriers to ART Adherence in Southern Africa: Challenges and Potential Ways Forward. *Global public health* **2011**, *6*, 83–97, doi:10.1080/17441691003796387.
188. Paschen-Wolff, M.M.; Reddy, V.; Matebeni, Z.; Southey-Swartz, I.; Sandfort, T. HIV and Sexually Transmitted Infection Knowledge among Women Who Have Sex with Women in Four Southern African Countries. *Culture, Health and Sexuality* **2020**, *22*, 705–721, doi:10.1080/13691058.2019.1629627.
189. Laisser, R.; Woods, R.; Bedwell, C.; Kasengele, C.; Nsemwa, L.; Kimaro, D.; Kuzenza, F.; Lyangenda, K.; Shayo, H.; Tuwele, K.; et al. The Tipping Point of Antenatal Engagement: A Qualitative Grounded Theory in Tanzania and Zambia. *Sexual & reproductive healthcare : official journal of the Swedish Association of Midwives* **2022**, *31*, doi:10.1016/J.SRHC.2021.100673.
190. Bedrosian, S.R.; Young, C.E.; Smith, L.A.; Cox, J.D.; Manning, C.; Pechta, L.; Telfer, J.L.; Gaines-McCollom, M.; Harben, K.; Holmes, W.; et al. Lessons of Risk Communication and Health Promotion - West Africa and United States. *MMWR supplements* **2016**, *65*, 68–74, doi:10.15585/MMWR.SU6503A10.
191. Fowler, R.A.; Fletcher, T.; Fischer, W.A.; Lamontagne, F.; Jacob, S.; Brett-Major, D.; Lawler, J.V.; Jacquerioz, F.A.; Houlihan, C.; O'Dempsey, T.; et al. Caring for Critically Ill Patients with Ebola Virus Disease. Perspectives from West Africa. *American journal of respiratory and critical care medicine* **2014**, *190*, 733–737, doi:10.1164/RCCM.201408-1514CP.
192. Munangatire, T.; Tomas, N.; Mareka, V. Nursing Students' Understanding of Health Literacy and Health Practices: A Cross-Sectional Study at a University in Namibia. *BMC nursing* **2022**, *21*, doi:10.1186/S12912-021-00776-Z.
193. Li, W.; Han, L.Q.; Guo, Y.J.; Sun, J. Using WeChat Official Accounts to Improve Malaria Health Literacy among Chinese Expatriates in Niger: An Intervention Study. *Malaria journal* **2016**, *15*, 1–13, doi:10.1186/S12936-016-1621-Y.
194. Abaraogu, U.O.; Ezema, C.I.; Igwe, S.E.; Egwuonwu, A.V.; Okafor, U.C. Work-Related Back Discomfort and Associated Factors among Automotive Maintenance Mechanics in Eastern Nigeria: A Cross Sectional Study. *Work* **2016**, *53*, 813–823, doi:10.3233/WOR-162247.
195. Abiodun, O.A.; Tunde-Ayinmode, M.F.; Ayinmode, B.A.; Adegunloye, O.A. Detecting Child Psychiatric Disorders during Routine Clinic Work: A Pre-Interventional Study of Primary Care Physicians in Ilorin, Nigeria. *South African Journal of Psychiatry* **2011**, *17*, 5, doi:10.4102/sajpsychiatry.v17i3.252.
196. Adanri, O.; Riedel, E.; Adanri, O.A. Maternal Health Literacy, Antenatal Care, and Pregnancy Outcomes, Walden Dissertations and Doctoral Studies, 2017.

197. Adedimeji, A.A.; Lounsbury, D.; Popoola, O.; Asuzu, C.; Lawal, A.; Oladoyin, V.; Crifase, C.; Agalliu, I.; Shankar, V.; Adebiyi, A. Improving Outcomes in Cancer Diagnosis, Prevention and Control: Barriers, Facilitators and the Need for Health Literacy in Ibadan Nigeria. *Psycho-Oncology* **2017**, *26*, 1455–1462, doi:10.1002/pon.4158.
198. Adefabi, R.A. Inclusive Economic Growth: The Pathway to Sustainable Development. *African Journal of Sustainable Development* **2018**, *8*, 13–29.
199. Adekoya-Cole, T.O.; Akinmokun, O.I.; Enweluzo, G.O.; Badmus, O.O.; Alabi, E.O. Poor Health Literacy in Nigeria<. Causes, Consequences and Measures to Improve It. *Nig. Qt J. Hosp. Med* **2015**, *25*, 112–117.
200. Ajuwon, G.A.; Ajuwon, A.J. Teaching High School Students to Use Online Consumer Health Resources on Mobile Phones: Outcome of a Pilot Project in Oyo State, Nigeria. *Journal of the Medical Library Association* **2019**, *107*, 194–202, doi:10.5195/jmla.2019.536.
201. Akangbe, J.A.; Asiyanbi, A.O.; Ibraheem, A.F.; Sola, K.E.; Animashaun, J.O. An Assessment of Health Practises among Small-Scale Farmers in Kwara State, Nigeria. *Annals of agricultural and environmental medicine : AAEM* **2015**, *22*, 488–490, doi:10.5604/12321966.1167720.
202. Al-Mujtaba, M.; Shobo, O.; Oyebola, B.C.; Ohemu, B.O.; Omale, I.; Shuaibu, A.; Anyanti, J. Assessing the Acceptability of Village Health Workers' Roles in Improving Maternal Health Care in Gombe State, Nigeria a Qualitative Exploration from Women Beneficiaries. *PloS one* **2020**, *15*, doi:10.1371/JOURNAL.PONE.0240798.
203. Aluh, D.O.; Dim, O.F.; Anene-Okeke, C.G. Mental Health Literacy among Nigerian Teachers. *Asia-Pacific Psychiatry* **2018**, *10*, doi:10.1111/appy.12329.
204. Aluh, D.O.; Okonta, M.J.; Odili, V.U. Cross-Sectional Survey of Mental Health Literacy among Undergraduate Students of the University of Nigeria. *BMJ open* **2019**, *9*, doi:10.1136/BMJOPEN-2019-028913.
205. Aluh, D.O.; Anyachebelu, O.C.; Ajaraonye, C.I. Comparison of Pharmacists' Mental Health Literacy: Developed versus Developing Countries. *Journal of the American Pharmacists Association* **2020**, *60*, S64–S72, doi:10.1016/j.japh.2020.05.003.
206. Anchang, K.Y.; Mbunwe, T.K. A Stated Preference Discrete Choice Health Literacy Intervention Framework for the Control of Non-Communicable Diseases (NCDs) in Africa. In *International Handbook of Health Literacy. Research, Practice and Policy across the Lifespan*; Okan, O., Bauer, U., Levin-Zamir, D., Pinheiro, P., Soerensen, K., Eds.; The Policy Press: Bristol, 2019; pp. 335–345 ISBN 1-4473-4451-0.
207. Arulogun, O.S.; Hurst, S.; Owolabi, M.O.; Akinyemi, R.O.; Uvere, E.; Saulson, R.; Ovbiagele, B. Experience of Using an Interdisciplinary Task Force to Develop a Culturally Sensitive Multipronged Tool to Improve Stroke Outcomes in Nigeria. *eNeurologicalSci* **2016**, *4*, 10–14, doi:10.1016/j.ensci.2016.04.003.
208. Atilola, O.; Olayiwola, F. The Nigerian Home Video Boom: Should Nigerian Psychiatrists Be Worried? Lessons from Content Review and Views of Community Dwellers. *International Journal of Social Psychiatry* **2012**, *58*, 470–476, doi:10.1177/0020764011408544.
209. Atilola, O.; Olayiwola, F. Stigmatisation of Mental Illness in Nigerian Home Videos.
210. Bella-Awusah, T.; Adedokun, B.; Dogra, N.; Omigbodun, O. The Impact of a Mental Health Teaching Programme on Rural and Urban Secondary School Students' Perceptions of Mental Illness in Southwest Nigeria. *Journal of Child and Adolescent Mental Health* **2014**, *26*, 207–215, doi:10.2989/17280583.2014.922090.
211. Dogra, N.; Omigbodun, O.; Adedokun, T.; Bella, T.; Ronzoni, P.; Adesokan, A. Nigerian Secondary School Children's Knowledge of and Attitudes to Mental Health and Illness. *Clinical Child Psychology and Psychiatry* **2012**, *17*, 336–353, doi:10.1177/1359104511410804.
212. Eguzo, K.; Camazine, B. Beyond Limitations: Practical Strategies for Improving Cancer Care in Nigeria. *Asian Pacific Journal of Cancer Prevention* **2013**, *14*, 3363–3368, doi:10.7314/APJCP.2013.14.5.3363.
213. Etokidem, A.; Nkpoyen, F.; Ekanem, C.; Mpama, E.; Isika, A. Potential Barriers to and Facilitators of Civil Society Organization Engagement in Increasing Immunization Coverage in Odukpani Local Government Area of Cross River State, Nigeria: An Implementation Research. *Health research policy and systems* **2021**, *19*, doi:10.1186/S12961-021-00697-Y.
214. Eze, B.I.; Okoye, O.; Aguwa, E.N. Public's Knowledge of the Differences between Ophthalmologists and Optometrists: A Critical Issue in Eye Care Service Utilisation. *International Journal of Ophthalmology* **2016**, doi:10.18240/ijo.2016.09.18.
215. Famuyiwa, A.O.; Entwistle, J.A. Characterising and Communicating the Potential Hazard Posed by Potentially Toxic Elements in Indoor Dusts from Schools across Lagos, Nigeria. *Environmental science. Processes & impacts* **2021**, *23*, 867–879, doi:10.1039/D0EM00445F.
216. Gabriel, I.; Creedy, D.; Coyne, E. Feasibility of a Socio-Spiritual Intervention to Improve Quality of Life of Adult Nigerians with Cancer and Their Family Caregivers: Protocol for a Randomised Controlled Trial. *Contemporary clinical trials communications* **2021**, *22*, doi:10.1016/J.CONCTC.2021.100802.
217. Gabriel, I.; Creedy, D.; Coyne, E. Quality of Life and Associated Factors among Adults Living with Cancer and Their Family Caregivers. *Nursing and Health Sciences* **2021**, *23*, 419–429, doi:10.1111/nhs.12823.
218. Hamilton-Ekeke, J.-T.; Abam, R.P.; Ogobiri, E. Health Literacy in the Promotion of Wellness among Secondary School Students in Bayelsa State, Nigeria. *Journal of Health Education Teaching* **2020**, *11*, 1–7.
219. Igwesi-Chidobe, C.N.; Kitchen, S.; Sorinola, I.O.; Godfrey, E.L. Evidence, Theory and Context: Using Intervention Mapping in the Development of a Community-Based Self-Management Program for Chronic Low Back Pain in a Rural African Primary Care Setting - the Good Back Program. *BMC public health* **2020**, *20*, doi:10.1186/S12889-020-8392-7.
220. Ikwuka, U.; Galbraith, M.N.; Manktelow, K.; Chen-Wilson, P. of P.J.; Oyebode, F.; Muomah, P.C.D.H.R.; Igboaka, A. Ideological vs. Instrumental Barriers to Accessing Formal Mental Health Care in the Developing World: Focus on South-Eastern Nigeria. *Journal of health care for the poor and underserved* **2016**, *21*, 157–175, doi:10.1353/HPU.2016.0025.
221. Kuyinu, Y.A.; Femi-Adebayo, T.T.; Adebayo, B.I.; Abdurraheem-Salami, I.; Odusanya, O.O. Health Literacy: Prevalence and Determinants in Lagos State, Nigeria. *PloS one* **2020**, *15*, doi:10.1371/JOURNAL.PONE.0237813.
222. Liu, D.Y.; Maki, A.W.; Maitland, A.; Meyer, E.R.; Sorensen, J.S.; Galvin, S. Enhancing Knowledge in Informal Settlements: Assessing Health Beliefs and Behaviors in Nigeria. *Annals of Global Health* **2020**, *86*, 121, doi:10.5334/aogh.2648.
223. Mojoyinola, J.K. Influence of Maternal Health Literacy on Healthy Pregnancy and Pregnancy Outcomes of Women Attending Public Hospitals in Ibadan, Oyo State, Nigeria. *An International Multi-Disciplinary Journal, Ethiopia* **2011**, *5*.
224. Nwaozuru, U.; Ezepue, C.; Iwelunmor, J.; Obiezu-Umeh, C.; Uzoaru, F.; Tshiswaka, D.I.; Okubadejo, N.; Edgell, R.; Ezechi, O.; Gbajabiamila, T.; et al. Addressing Stroke Literacy in Nigeria Through Music: A Qualitative Study of Community Perspectives. *Journal of stroke and cerebrovascular diseases : the official journal of National Stroke Association* **2020**, *29*, doi:10.1016/J.JSTROKECEREBROVASDIS.2020.105312.

225. Obaremi, O.D.; Olatokun, W.M. A Survey of Health Information Source Use in Rural Communities Identifies Complex Health Literacy Barriers. *Health information and libraries journal* **2022**, *39*, 59–67, doi:10.1111/hir.12364.
226. Obasola, O.I.; Mabawonku, I.M. Mothers' Perception of Maternal and Child Health Information Disseminated via Different Modes of ICT in Nigeria. *Health information and libraries journal* **2018**, *35*, 309–318, doi:10.1111/HIR.12235.
227. Ofole, N.M.; Ohakwe, P.C.N. Therapeutic Outcome of Self-Control and Social Interaction Interventions on Negative Body Image among In-School Adolescents with Low Health Literacy in Southwest Nigeria. *International Journal of Education and Literacy Studies* **2021**, *9*, 238–246, doi:10.7575/aiac.ijels.v.9n.4p.238.
228. Ogunrin, O.; Taiwo, F.; Frith, L. Genomic Literacy and Awareness of Ethical Guidance for Genomic Research in Sub-Saharan Africa: How Prepared Are Biomedical Researchers? *Journal of empirical research on human research ethics : JERHRE* **2019**, *14*, 78–87, doi:10.1177/1556264618805194.
229. Ohaeri, J.U.; Fido, A.A. The Opinion of Caregivers on Aspects of Schizophrenia and Major Affective Disorders in a Nigerian Setting. *Soc Psychiatry Psychiatr Epidemiol* **2001**, *36*, 493–499.
230. O, O.A.; O, A.A.; O, C.E.; K, I.O.; O, O.O.; Oladunjoye, A.O. Health Literacy Amongst Tuberculosis Patient in a General Hospital in North Central Nigeria. *Journal of Community Medicine and Primary Health Care* **2012**, *24*.
231. Olusegun, S.T. The Roles of Yoruba Songs on Pregnancy, Labour and Baby Care in Antenatal and Postnatal Clinic in Southwestern Nigerian Hospitals. *Lwati: A Journal of Contemporary Research* **2019**, *16*, 116–135, doi:10.4314/lwati.v6i1.
232. Sokefun, E.E.; Atulomah, N.O. Predictors of Infant-Survival Practices among Mothers Attending Paediatric Clinics in Ijebu-Ode, Ogun State, Nigeria. *BMC Public Health* **2020**, *20*, doi:10.1186/s12889-020-09310-3.
233. Ukpabi, N.S. Relationship Among Health Literacy Superstitious Beliefs and Relationship Among Health Literacy Superstitious Beliefs and Self-Care Among Diabetic Patients in Warri, Nigeria Self-Care Among Diabetic Patients in Warri, Nigeria, Walden University, 2021.
234. Ukwenya, V.O.; Fuwape, T.A.; Ilesanmi, O.S. *COVID-19 Health Literacy, Coping Strategies and Perception of COVID-19 Containment Measures among Community Members in a Southwestern State in Nigeria*; 2021; Vol. 11, p. 478;.
235. Uwatt, L.E.; Ogar, D.I.; Odey, J.E. Literacy and Health Seeking Behaviours among Patients 57 Literacy and Health Seeking Behaviours among Patients in Benue and Cross River States of Nigeria. *LWATI: A Journal of Contemporary Research* **2010**, *7*, 58–63.
236. Väisänen, H.; Moore, A.M.; Owolabi, O.; Stillman, M.; Fatusi, A.; Akinyemi, A. Sexual and Reproductive Health Literacy, Misoprostol Knowledge and Use of Medication Abortion in Lagos State, Nigeria: A Mixed Methods Study. *Studies in family planning* **2021**, *52*, 217–237, doi:10.1111/SIFP.12156.
237. Mbada, C.E.; Johnson, O.E.; Oyewole, O.O.; Adejube, O.J.; Fatoye, C.; Idowu, O.A.; Odeyemi, R.V.; Akinirinbola, K.B.; Ganiyu, D.; Fatoye, F. Cultural Adaptation and Psychometric Evaluation of the Yoruba Version of the Health Literacy Questionnaire. *Annali di igiene : medicina preventiva e di comunita* **2022**, *34*, 54–69, doi:10.7416/ai.2021.2470.
238. Cubaka, V.K.; Schriver, M.; Kayitare, J.B.; Cotton, P.; Maindal, H.T.; Nyirazinyoye, L.; Kallestrup, P. "He Should Feel Your Pain": Patient Insights on Patient-Provider Communication in Rwanda. *African journal of primary health care & family medicine* **2018**, *10*, doi:10.4102/PHCFM.V10I1.1514.
239. Ingabire, C.M.; Hakizimana, E.; Kateera, F.; Rulisa, A.; Borne, B.V.D.; Nieuwold, I.; Muvunyi, C.; Koenraadt, C.J.M.; Vugt, M.V.; Mutesa, L.; et al. Using an Intervention Mapping Approach for Planning, Implementing and Assessing a Community-Led Project towards Malaria Elimination in the Eastern Province of Rwanda. *Malaria journal* **2016**, *15*, doi:10.1186/S12936-016-1645-3.
240. Linden, A.F.; Maine, R.G.; Hedt-Gauthier, B.L.; Kamanzi, E.; Gauvey-Kern, K.; Mody, G.; Ntakiyiruta, G.; Kansayisa, G.; Ntaganda, E.; Niyonkuru, F.; et al. Validation of a Community-Based Survey Assessing Nonobstetric Surgical Conditions in Burera District, Rwanda. *Surgery (United States)* **2016**, *159*, 1217–1226, doi:10.1016/j.surg.2015.10.012.
241. Lygidakis, C.; Uwizihiwe, J.P.; Kallestrup, P.; Bia, M.; Condo, J.; Vögele, C. Community- and mHealth-Based Integrated Management of Diabetes in Primary Healthcare in Rwanda (D^2Rwanda): The Protocol of a Mixed-Methods Study Including a Cluster Randomised Controlled Trial. *BMJ open* **2019**, *9*, doi:10.1136/BMJOPEN-2018-028427.
242. Mukanoheli, V.; Uwamahoro, M.C.; Mbarushimana, V.; Meharry, P. Functional Health Literacy and Self-Care Behaviors Among Type 2 Diabetic Patients at a University Teaching Hospital in Kigali. *Rwanda Journal of Medicine and Health Sciences* **2020**, *3*, 49–59, doi:10.4314/rjmhs.v3i1.7.
243. Umubyeyi, A.; Mogren, I.; Ntaganira, J.; Krantz, G. Help-Seeking Behaviours, Barriers to Care and Self-Efficacy for Seeking Mental Health Care: A Population-Based Study in Rwanda. *Social psychiatry and psychiatric epidemiology* **2016**, *51*, 81–92, doi:10.1007/S00127-015-1130-2.
244. Dieng, S.; Cisse, D.; Lombrail, P.; Azogui-Lévy, S. Mothers' Oral Health Literacy and Children's Oral Health Status in Pikine, Senegal: A Pilot Study. *PloS one* **2020**, *15*, doi:10.1371/JOURNAL.PONE.0226876.
245. Glik, D.; Massey, P.; Gipson, J.; Dieng, T.; Rideau, A.; Prelip, M. Health-Related Media Use among Youth Audiences in Senegal. *Health promotion international* **2016**, *31*, 73–82, doi:10.1093/HEAPRO/DAU060.
246. Kim, T.Y.; Haider, M.; Hancock, G.R.; Boudreaux, M.H. The Role of Health Literacy in Family Planning Use among Senegalese Women. *Journal of Health Communication* **2019**, *24*, 244–261, doi:10.1080/10810730.2019.1601299.
247. Kennedy, A.; Abosi, U.; Gilbert, C.; Mustapha, J. Factors Associated with Adherence to Treatment in Patients with Open Angle Glaucoma in Sierra Leone, West Africa: Patient Demographics and Questionnaire. *International Ophthalmology* **2022**, doi:10.1007/s10792-022-02347-w.
248. Keles, E.; Hassan-Kadle, M.A.; Osman, M.M.; Eker, H.H.; Abusoglu, Z.; Baydili, K.N.; Osman, A.M. Clinical Characteristics of Acute Liver Failure Associated with Hepatitis A Infection in Children in Mogadishu, Somalia: A Hospital-Based Retrospective Study. *BMC infectious diseases* **2021**, *21*, doi:10.1186/S12879-021-06594-7.
249. Aggarwal, S.; Taljard, L.; Wilson, Z.; Berk, M. Evaluation of Modified Patient Health Questionnaire-9 Teen in South African Adolescents. *Indian Journal of Psychological Medicine* **2017**, *39*, 143–145, doi:10.4103/0253-7176.203124.
250. Aggarwal, S.; Berk, M.; Taljard, L.; Wilson, Z. South African Adolescents' Beliefs about Depression. *International Journal of Social Psychiatry* **2016**, *62*, 198–200, doi:10.1177/0020764015618226.

251. Andersson, L.M.C.; Schierenbeck, I.; Strumpher, J.; Krantz, G.; Topper, K.; Backman, G.; Ricks, E.; Rooyen, D.V. Help-Seeking Behaviour, Barriers to Care and Experiences of Care among Persons with Depression in Eastern Cape, South Africa. *Journal of Affective Disorders* **2013**, *151*, 439–448, doi:10.1016/j.jad.2013.06.022.
252. Babatunde, G.B.; Rensburg, A.J.V.; Bhana, A.; Petersen, I. Stakeholders' Perceptions of Child and Adolescent Mental Health Services in a South African District: A Qualitative Study. *International journal of mental health systems* **2020**, *14*, doi:10.1186/S13033-020-00406-2.
253. Babatunde, G.B.; Rensburg, A.J. van; Bhana, A.; Petersen, I. Identifying Multilevel and Multisectoral Strategies to Develop a Theory of Change for Improving Child and Adolescent Mental Health Services in a Case-Study District in South Africa. *Child and adolescent psychiatry and mental health* **2022**, *16*, doi:10.1186/S13034-022-00484-9.
254. Bennin, F.; Rother, H.A. "But It's Just Paracetamol": Caregivers' Ability to Administer over-the-Counter Painkillers to Children with the Information Provided. *Patient education and counseling* **2015**, *98*, 331–337, doi:10.1016/J.PEC.2014.11.025.
255. Bobbins, A.C.; Manhanzva, R.; Bhandankar, M.; Srinivas, S.C. Balanced Nutrition and Hand Hygiene for Children in South Africa. *Health promotion international* **2019**, *34*, 333–343, doi:10.1093/HEAPRO/DAX090.
256. Braathen, S.H.; Vergunst, R.; Mji, G.; Mannan, H.; Swartz, L. Understanding the Local Context for the Application of Global Mental Health: A Rural South African Experience. *International Health* **2013**, *5*, 38–42, doi:10.1093/inthealth/ihs016.
257. Campbell, M.M.; Matshabane, O.P.; Mqulwana, S.; Mndini, M.; Nagdee, M.; Stein, D.J.; Vries, J.D. Evaluating Community Engagement Strategies to Manage Stigma in Two African Genomics Studies Involving People Living with Schizophrenia or Rheumatic Heart Disease. *Global Health* **2021**, *2021*, 1–9, doi:10.1155/2021/9926495.
258. Clarke, H.; Voss, M. The Role of a Multidisciplinary Student Team in the Community Management of Chronic Obstructive Pulmonary Disease. *Primary health care research & development* **2016**, *17*, 415–420, doi:10.1017/S1463423616000013.
259. Davis, B.; Jansen, C.J.M. Deploying a Fotonovela to Combat Methamphetamine Abuse among South Africans with Varying Levels of Health Literacy. *International journal of environmental research and public health* **2021**, *18*, doi:10.3390/IJERPH18126334.
260. De, V.; Martins-Reis, O.; Santos, J.N. Maximizing Health Literacy and Client Recall in a Developing Context: Speech-Language Therapist and Client Perspectives. *International Journal of Language and Communication Disorders* **2012**, *17*, 113–117.
261. Sorsdahl, K.R.; Flisher, A.J.; Wilson, Z.; Stein, D.J. Explanatory Models of Mental Disorders and Treatment Practices among Traditional Healers in Mpumulanga, South Africa. *African journal of psychiatry* **2010**, *13*, 284–290, doi:10.4314/AJPSY.V13I4.61878.
262. Devillé, W.; Tempelman, H. Feasibility and Robustness of an Oral HIV Self-Test in a Rural Community in South-Africa: An Observational Diagnostic Study. *PloS one* **2019**, *14*, doi:10.1371/JOURNAL.PONE.0215353.
263. Dowse, R.; Lecoko, L.; Ehlers, M.S. Applicability of the REALM Health Literacy Test to an English Second-Language South African Population. *Pharmacy World & Science* **2010**, *32*, 464–471, doi:10.1007/s11096-010-9392-y.
264. Ducray, J.F.; Kell, C.M.; Basdav, J.; Haffejee, F. Cervical Cancer Knowledge and Screening Uptake by Marginalized Population of Women in Inner-City Durban, South Africa: Insights into the Need for Increased Health Literacy. *Women's health (London, England)* **2021**, *17*, doi:10.1177/17455065211047141.
265. Engelbrecht, M.C.; Kigozi, N.G.; Heunis, J.C. Factors Associated with Limited Vaccine Literacy: Lessons Learnt from COVID-19. *Vaccines* **2022**, *10*, 865, doi:10.3390/vaccines10060865.
266. Etheredge, H.; Penn, C.; Watermeyer, J. Opt-in or Opt-out to Increase Organ Donation in South Africa? Appraising Proposed Strategies Using an Empirical Ethics Analysis. *Developing World Bioethics* **2018**, *18*, 119–125, doi:10.1111/dewb.12154.
267. Field, S.; Abrahams, Z.; Woods, D.L.; Turner, R.; Onah, M.N.; Kaura, D.K.; Honikman, S. Accessible Continued Professional Development for Maternal Mental Health. *African journal of primary health care & family medicine* **2019**, *11*, e1–e7, doi:10.4102/phcfm.v11i1.1902.
268. Fincham, D.; Kagee, A.; ChB, M.M.M. Dietary and Fluid Adherence among Haemodialysis Patients Attending Public Sector Hospitals in the Western Cape. *S Afr J Clin Nutr* **2008**, *21*, 7–12.
269. Galappaththi-Arachchige, H.N.; Zulu, S.G.; Kleppa, E.; Lillebo, K.; Qvigstad, E.; Ndhlovu, P.; Vennervald, B.J.; Gundersen, S.G.; Kjetland, E.F.; Taylor, M. Reproductive Health Problems in Rural South African Young Women: Risk Behaviour and Risk Factors. *Reproductive health* **2018**, *15*, doi:10.1186/S12978-018-0581-9.
270. Gordon, T.; Booysen, F.; Mbonigaba, J. Socio-Economic Inequalities in the Multiple Dimensions of Access to Healthcare: The Case of South Africa. *BMC public health* **2020**, *20*, doi:10.1186/S12889-020-8368-7.
271. Haricharan, H.J.; Heap, M.; Hacking, D.; Lau, Y.K. Health Promotion via SMS Improves Hypertension Knowledge for Deaf South Africans. *BMC public health* **2017**, *17*, doi:10.1186/S12889-017-4619-7.
272. Hathorn, S.K.; Lochner, C.; Stein, D.J.; Bantjes, J. Help-Seeking Intention in Obsessive-Compulsive Disorder: Predictors and Barriers in South Africa. *Frontiers in psychiatry* **2021**, *12*, doi:10.3389/FPSYT.2021.733773.
273. Hunter-Adams, J.; Rother, H.A. A Qualitative Study of Language Barriers between South African Health Care Providers and Cross-Border Migrants. *BMC health services research* **2017**, *17*, 1–9, doi:10.1186/S12913-017-2042-5.
274. Hussain-Alkhateeb, L.; Fottrell, E.; Petzold, M.; Kahn, K.; Byass, P. Local Perceptions of Causes of Death in Rural South Africa: A Comparison of Perceived and Verbal Autopsy Causes of Death. *Global health action* **2015**, *8*, doi:10.3402/GHA.V8.28302.
275. Joubert, K.; Githinji, E. Quality and Readability of Information Pamphlets on Hearing and Paediatric Hearing Loss in the Gauteng Province, South Africa. *International Journal of Pediatric Otorhinolaryngology* **2014**, *78*, 354–358, doi:10.1016/j.ijporl.2013.12.018.
276. Kagee, A. Adherence to Antiretroviral Therapy in the Context of the National Roll-Out in South Africa: Defining a Research Agenda for Psychology. *South African Journal of Psychology* **2008**, *38*, 413–428, doi:10.1177/008124630803800211.

277. Keikelame, M.J.; Swartz, L. Lost Opportunities to Improve Health Literacy: Observations in a Chronic Illness Clinic Providing Care for Patients with Epilepsy in Cape Town South Africa. *Epilepsy & behavior : E&B* **2013**, *26*, 36–41, doi:10.1016/J.YEBEH.2012.10.015.
278. Kemp, C.G.; Mntambo, N.; Weiner, B.J.; Grant, M.; Rao, D.; Bhana, A.; Gigaba, S.G.; Luvuno, Z.P.B.; Simoni, J.M.; Hughes, J.P.; et al. Pushing the Bench: A Mixed Methods Study of Barriers to and Facilitators of Identification and Referral into Depression Care by Professional Nurses in KwaZulu-Natal, South Africa. *SSM. Mental health* **2021**, *1*, 100009, doi:10.1016/J.SSMMH.2021.100009.
279. Kometsi, M.J.; Mkhize, N.J.; Pillay, A.L. Mental Health Literacy: Conceptions of Mental Illness among African Residents of Sisonke District in KwaZulu-Natal, South Africa. *South African Journal of Psychology* **2020**, *50*, 347–358, doi:10.1177/0081246319891635.
280. Korhonen, J.; Axelin, A.; Grobler, G.; Lahti, M. Content Validation of Mental Health Literacy Scale (MHLS) for Primary Health Care Workers in South Africa and Zambia ─ a Heterogeneous Expert Panel Method. *Global health action* **2019**, *12*, doi:10.1080/16549716.2019.1668215.
281. Kubheka, B.Z.; Carter, V.; Mwaura, J. Social Media Health Promotion in South Africa: Opportunities and Challenges. *African journal of primary health care & family medicine* **2020**, *12*, doi:10.4102/PHCFM.V12I1.2389.
282. Lopes, H.; McKay, V. Adult Learning and Education as a Tool to Contain Pandemics: The COVID-19 Experience. *International Review of Education* **2020**, *66*, 575–602, doi:10.1007/s11159-020-09843-0.
283. Lothe, A.; Zulu, N.; Øyhus, A.O.; Kjetland, E.F.; Taylor, M. Treating Schistosomiasis among South African High School Pupils in an Endemic Area, a Qualitative Study. *BMC infectious diseases* **2018**, *18*, doi:10.1186/S12879-018-3102-0.
284. Madlala, D.; Joubert, P.M.; Masenge, A. Community Mental Health Literacy in Tshwane Region 1: A Quantitative Study. *The South African journal of psychiatry : SAJP : the journal of the Society of Psychiatrists of South Africa* **2022**, *28*, doi:10.4102/SAJPSYCHIATRY.V28I0.1661.
285. Mafutha, N.G.; Mogotlane, S.; Swardt, H.C. de Development of a Hypertension Health Literacy Assessment Tool for Use in Primary Healthcare Clinics in South Africa, Gauteng. *African journal of primary health care & family medicine* **2017**, *9*, doi:10.4102/PHCFM.V9I1.1305.
286. Mall, S.; Sorsdahl, K.; Struthers, H.; Joska, J.A. Mental Health in Primary Human Immunodeficiency Virus Care in South Africa: A Study of Provider Knowledge, Attitudes, and Practice. *Journal of Nervous and Mental Disease* **2013**, *201*, 196–201, doi:10.1097/NMD.0b013e3182845c24.
287. Manhanzva, R.; Marara, P.; Duxbury, T.; Bobbins, A.C.; Pearse, N.; Hoel, E.; Mzizi, T.; Srinivas, S.C. Gender and Leadership for Health Literacy to Combat the Epidemic Rise of Noncommunicable Diseases. *Health care for women international* **2017**, *38*, 833–847, doi:10.1080/07399332.2017.1332062.
288. Masuku, K.P.; Mophosho, M.; Tshabalala, M. "I Felt Pain. Deep Pain…": Experiences of Primary Caregivers of Stroke Survivors with Aphasia in a South African Township. *African journal of disability* **2018**, *7*, doi:10.4102/AJOD.V7I0.368.
289. Matima, R.; Murphy, K.; Levitt, N.S.; BeLue, R.; Oni, T. A Qualitative Study on the Experiences and Perspectives of Public Sector Patients in Cape Town in Managing the Workload of Demands of HIV and Type 2 Diabetes Multimorbidity. *PloS one* **2018**, *13*, doi:10.1371/JOURNAL.PONE.0194191.
290. Medina-Marino, A.; Glockner, K.; Grew, E.; Vos, L.D.; Olivier, D.; Klausner, J.; Daniels, J. The Role of Trust and Health Literacy in Nurse-Delivered Point-of-Care STI Testing for Pregnant Women Living with HIV, Tshwane District, South Africa. *BMC public health* **2020**, *20*, doi:10.1186/S12889-020-08689-3.
291. Mhlongo, M.; Marara, P.; Bradshaw, K.; Srinivas, S.C. Health Education on Diabetes at a South African National Science Festival. *African Journal of Health Professions Education* **2018**, *10*, 26, doi:10.7196/AJHPE.2018.v10i1.887.
292. Minty, Y.; Moosa, M.Y.H.; Jeenah, F.Y. Mental Illness Attitudes and Knowledge in Non-Specialist Medical Doctors Working in State and Private Sectors. *The South African journal of psychiatry : SAJP : the journal of the Society of Psychiatrists of South Africa* **2021**, *27*, doi:10.4102/SAJPSYCHIATRY.V27I0.1592.
293. Mohamed-Kaloo, Z.; Laher, S. Perceptions of Mental Illness among Muslim General Practitioners in South Africa. *South African medical journal = Suid-Afrikaanse tydskrif vir geneeskunde* **2014**, *104*, 350–352, doi:10.7196/SAMJ.7863.
294. Mokwena, K.E.; Ndlovu, J. Why Do Patients with Mental Disorders Default Treatment? A Qualitative Enquiry in Rural Kwazulu-Natal, South Africa. *Healthcare (Basel, Switzerland)* **2021**, *9*, doi:10.3390/HEALTHCARE9040461.
295. Molete, M.P.; Daly, B.; Hlungwani, T.M. Oral Health Promotion in Gauteng: A Qualitative Study. *Global Health Promotion* **2013**, *20*, 50–58, doi:10.1177/1757975913476906.
296. Monnapula-Mazabane, P.; Petersen, I. Mental Health Stigma Experiences among Caregivers and Service Users in South Africa: A Qualitative Investigation. *Current psychology (New Brunswick, N.J.)* **2021**, doi:10.1007/S12144-021-02236-Y.
297. Moodley, N.; Saimen, A.; Zakhura, N.; Motau, D.; Setswe, G.; Charalambous, S.; Chetty-Makkan, C.M. "They Are Inconveniencing Us" - Exploring How Gaps in Patient Education and Patient Centred Approaches Interfere with TB Treatment Adherence: Perspectives from Patients and Clinicians in the Free State Province, South Africa. *BMC public health* **2020**, *20*, 454, doi:10.1186/s12889-020-08562-3.
298. Moroe, N.F. Occupational Noise Induced Hearing Loss in the Mining Sector in South Africa: Perspectives from Occupational Health Practitioners on How Mineworkers Are Trained. *The South African journal of communication disorders = Die Suid-Afrikaanse tydskrif vir Kommunikasieafwykings* **2020**, *67*, 1–6, doi:10.4102/SAJCD.V67I2.676.
299. Müller, I.; Smith, D.; Adams, L.; Aerts, A.; Damons, B.P.; Degen, J.; Gall, S.; Gani, Z.; Gerber, M.; Gresse, A.; et al. Effects of a School-Based Health Intervention Program in Marginalized Communities of Port Elizabeth, South Africa (the KaziBantu Study): Protocol for a Randomized Controlled Trial. *JMIR Research Protocols* **2019**, *8*, e14097, doi:10.2196/14097.
300. Murphy, K.; Chuma, T.; Mathews, C.; Steyn, K.; Levitt, N. A Qualitative Study of the Experiences of Care and Motivation for Effective Self-Management among Diabetic and Hypertensive Patients Attending Public Sector Primary Health Care Services in South Africa. *BMC health services research* **2015**, *15*, doi:10.1186/S12913-015-0969-Y.
301. Musakwa, N.O.; Bor, J.; Nattey, C.; Lönnermark, E.; Nyasulu, P.; Long, L.; Evans, D. Perceived Barriers to the Uptake of Health Services among First-Year University Students in Johannesburg, South Africa. *PloS one* **2021**, *16*, doi:10.1371/JOURNAL.PONE.0245427.

302. Naidoo, S.; Taylor, M. HIV Health Literacy, Sexual Behaviour and Self-Reports of Having Tested for HIV among Students. *African Journal of AIDS Research* **2015**, *14*, 107–115, doi:10.2989/16085906.2015.1040808.
303. Noncungu, T.M.; Chipps, J.A. Information-Seeking in First Visit Pregnant Women in Khayelitsha, South Africa. *Health SA = SA Gesondheid* **2020**, *25*, 1–6, doi:10.4102/HSAG.V25I0.1478.
304. Norris, S.A.; Draper, C.E.; Prioreschi, A.; Smuts, C.M.; Ware, L.J.; Dennis, C.L.; Awadalla, P.; Bassani, D.; Bhutta, Z.; Briollais, L.; et al. Building Knowledge, Optimising Physical and Mental Health and Setting up Healthier Life Trajectories in South African Women (Bukhali): A Preconception Randomised Control Trial Part of the Healthy Life Trajectories Initiative (HeLTI). *BMJ open* **2022**, *12*, e059914, doi:10.1136/BMJOPEN-2021-059914.
305. Patel, S.; Dowse, R. Understanding the Medicines Information-Seeking Behaviour and Information Needs of South African Long-Term Patients with Limited Literacy Skills. *Health Expectations* **2015**, *18*, 1494–1507, doi:10.1111/hex.12131.
306. Petersen, I.; Lund, C. Mental Health Service Delivery in South Africa from 2000 to 2010: One Step Forward, One Step Back. *South African Medical Journal* **2011**, *101*, 751757.
307. Rensburg, Z.J. van Levels of Health Literacy and English Comprehension in Patients Presenting to South African Primary Healthcare Facilities. *African journal of primary health care & family medicine* **2020**, *12*, doi:10.4102/PHCFM.V12I1.2047.
308. Sande, M. van de; Dippenaar, H.; Rutten, G.E.H.M. The Relationship between Patient Education and Glycaemic Control in a South African Township. *Primary Care Diabetes* **2007**, *1*, 87–91, doi:10.1016/j.pcd.2007.04.007.
309. Selohilwe, O.; Bhana, A.; Garman, E.C.; Petersen, I. Evaluating the Role of Levels of Exposure to a Task Shared Depression Counselling Intervention Led by Behavioural Health Counsellors: Outcome and Process Evaluation. *International journal of mental health systems* **2019**, *13*, doi:10.1186/S13033-019-0299-2.
310. Smythe, T.; Inglis-Jassiem, G.; Conradie, T.; Kamalakannan, S.; Fernandes, S.; van-Niekerk, S.; English, R.; Webster, J.; Hameed, S.; Louw, Q. Access to Health Care for People with Stroke in South Africa: A Qualitative Study of Community Perspectives. *BMC health services research* **2022**, *22*, doi:10.1186/S12913-022-07903-9.
311. Sorsdahl, K.R.; Mall, S.; Stein, D.J.; Joska, J.A. Perspectives towards Mental Illness in People Living with HIV/AIDS in South Africa. *AIDS Care - Psychological and Socio-Medical Aspects of AIDS/HIV* **2010**, *22*, 1418–1427, doi:10.1080/09540121003758655.
312. Sorsdahl, K.; Stein, D.J.; Myers, B. Negative Attributions towards People with Substance Use Disorders in South Africa: Variation across Substances and by Gender. *BMC psychiatry* **2012**, *12*, doi:10.1186/1471-244X-12-101.
313. Sorsdahl, K.; Stein, D.J.; Lund, C. Mental Health Services in South Africa: Scaling up and Future Directions. *Afr J Psychiatry (Johannesbg)* **2012**, *15*, 168–171, doi:10.4314/ajpsy.v15i3.21.
314. Spedding, M.F.; Stein, D.J.; Naledi, T.; Sorsdahl, K. Pregnant Women's Mental Health Literacy and Perceptions of Perinatal Mental Disorders in the Western Cape, South Africa. *Mental Health and Prevention* **2018**, *11*, 16–23, doi:10.1016/j.mhp.2018.05.002.
315. Strecker, M.; Stuttaford, M.; London, L. Health Rights Pamphlets: Critical Literacy and Inclusive Citizenship, South Africa. *Health promotion international* **2014**, *29*, 339–348, doi:10.1093/HEAPRO/DAS067.
316. Surka, S.; Steyn, K.; Everett-Murphy, K.; Gaziano, T.A.; Levitt, N. Knowledge and Perceptions of Risk for Cardiovascular Disease: Findings of a Qualitative Investigation from a Low-Income Peri-Urban Community in the Western Cape, South Africa. *African journal of primary health care & family medicine* **2015**, *7*, doi:10.4102/PHCFM.V7I1.891.
317. Thutloa, A.; Stroud, C. Does Active Participation in Health Enhance Health Outcomes and Healthcare Delivery Systems? *Stellenbosch Papers in Linguistics Plus* **2013**, *41*, doi:10.5842/41-0-87.
318. Treffry-Goatley, A.; Lessells, R.J.; Moletsane, R.; Oliveira, T.D.; Gaede, B. Community Engagement with HIV Drug Adherence in Rural South Africa: A Transdisciplinary Approach. *Medical humanities* **2018**, *44*, 239–246, doi:10.1136/MEDHUM-2018-011474.
319. Wasserman, Z.; Wright, S.C.D.; Maja, T.M. Assessment of the English Literacy Level of Patients in Primary Health Care Services in Tshwane, Gauteng Province: Part 2. *Health SA Gesondheid* **2010**, *15*, doi:10.4102/hsag.v15i1.469.
320. Wasserman, Z.; Wright, S.C.D.; Maja, T.M. Adaptation of the Rapid Estimate of Adult Litera Cy in Medicine Revised (REALM -R) to the South African Context: Part 1. *Health SA Gesondheid* **2010**, *15*, doi:10.4102/hsag.v15i1.468.
321. Worley, S.; Didiza, Z.; Nomatshila, S.; Porter, S.; Makwedini, N.; Macharia, D.; Hoos, D. Wellness Programmes for Persons Living with HIV/AIDS: Experiences from Eastern Cape Province, South Africa. *Global Public Health* **2009**, *4*, 367–385, doi:10.1080/17441690801994301.
322. Wouters, E.; Damme, W.V.; Loon, F.V.; Rensburg, D. van; Meulemans, H. Public-Sector ART in the Free State Province, South Africa: Community Support as an Important Determinant of Outcome. *Social Science and Medicine* **2009**, *69*, 1177–1185, doi:10.1016/j.socscimed.2009.07.034.
323. Zanoni, B.C.; Archary, M.; Sibaya, T.; Musinguzi, N.; Kelley, M.E.; McManus, S.; Haberer, J.E. Development and Validation of the HIV Adolescent Readiness for Transition Scale (HARTS) in South Africa. *Journal of the International AIDS Society* **2021**, *24*, doi:10.1002/JIA2.25767.
324. Atilola, O. Mental Health Service Utilization in Sub-Saharan Africa: Is Public Mental Health Literacy the Problem? Setting the Perspectives Right. *Global Health Promotion* **2016**, *23*, 30–37, doi:10.1177/1757975914567179.
325. Atilola, O. Level of Community Mental Health Literacy in Sub-Saharan Africa: Current Studies Are Limited in Number, Scope, Spread, and Cognizance of Cultural Nuances. *Nordic Journal of Psychiatry* **2015**, *69*, 93–101, doi:10.3109/08039488.2014.947319.
326. Beia, T.; Kielmann, K.; Diaconu, K. Changing Men or Changing Health Systems? A Scoping Review of Interventions, Services and Programmes Targeting Men's Health in Sub-Saharan Africa. *International journal for equity in health* **2021**, *20*, doi:10.1186/S12939-021-01428-Z.
327. Dzifa, D.; Boima, V.; Yorke, E.; Yawson, A.; Ganu, V.; Mate-Kole, C. Predictors and Outcome of Systemic Lupus Erythematosus (SLE) Admission Rates in a Large Teaching Hospital in Sub-Saharan Africa. *Lupus* **2018**, *27*, 336–342, doi:10.1177/0961203317742710.
328. Mutebi, M.; Scroggins, D.; Simons, V.; Oti, N.O.; Hammad, N. Engaging Patients for Clinical Trials in Africa: Patient-Centered Approaches. *JCO global oncology* **2020**, *6*, 942–947, doi:10.1200/JGO.19.00190.

329. Okumu, M.; Nyoni, T.; Byansi, W. Alleviating Psychological Distress and Promoting Mental Wellbeing among Adolescents Living with HIV in Sub-Saharan Africa, during and after COVID-19. *Global public health* **2021**, *16*, 964–973, doi:10.1080/17441692.2021.1912137.
330. Sarfo, F.S.; Ovbiagele, B. Stroke Minimization through Additive Anti-Atherosclerotic Agents in Routine Treatment (SMAART): A Pilot Trial Concept for Improving Stroke Outcomes in Sub-Saharan Africa. *Journal of the neurological sciences* **2017**, *377*, 167–173, doi:10.1016/J.JNS.2017.04.012.
331. Thompson, J.; Havenga, Y.; Naude, S. The Health Literacy Needs of Women Living with HIV/AIDS. *Health SA Gesondheid* **2015**, *20*, 11–21, doi:10.1016/j.hsag.2015.03.001.
332. Wiginton, J.M.; Mathur, S.; Gottert, A.; Pilgrim, N.; Pulerwitz, J. Hearing From Men Living With HIV: Experiences With HIV Testing, Treatment, and Viral Load Suppression in Four High-Prevalence Countries in Sub-Saharan Africa. *Frontiers in public health* **2022**, *10*, doi:10.3389/FPUBH.2022.861431.
333. Mohale, H.; Sweet, L.; Graham, K. Maternity Health Care: The Experiences of Sub-Saharan African Women in Sub-Saharan Africa and Australia. *Women and Birth* **2017**, *30*, 298–307, doi:10.1016/j.wombi.2016.11.011.
334. Bastien, S. Access, Agency and Ambiguity: Communication about AIDS among Young People in Northern Tanzania. *Culture, health & sexuality* **2009**, *11*, 751–765, doi:10.1080/13691050903362632.
335. Dalsmo, I.E.; Haraldstad, K.; Johannessen, B.; Hovland, O.J.; Chiduo, M.G.; Fegran, L. "Now I Feel That I Can Achieve Something": Young Tanzanian Women's Experiences of Empowerment by Participating in Health Promotion Campaigns. *International journal of environmental research and public health* **2021**, *18*, doi:10.3390/IJERPH18168747.
336. Higgins, C. Constructing Identities through Literacy Events in HIV/AIDS Education. *Journal of Multilingual and Multicultural Development* **2014**, *35*, 709–723, doi:10.1080/01434632.2014.908892.
337. Higgins, C. Discursive Enactments of the World Health Organization's Policies: Competing Cultural Models in Tanzanian HIV/AIDS Prevention. *Language Policy* **2010**, *9*, 65–85, doi:10.1007/s10993-009-9151-x.
338. Hovland, O.J.; Hole, A.F.; Chiduo, M.G.; Johannessen, B. Experiences from Cross-Cultural Collaboration in Health Campaigns in Tanzania: A Qualitative Study. *Archives of public health = Archives belges de sante publique* **2021**, *79*, doi:10.1186/S13690-021-00730-0.
339. Kutcher, S.; Wei, Y.; Gilberds, H.; Brown, A.; Ubuguyu, O.; Njau, T.; Sabuni, N.; Magimba, A.; Perkins, K. The African Guide: One Year Impact and Outcomes from the Implementation of a School Mental Health Literacy Curriculum Resource in Tanzania. *Journal of Education and Training Studies* **2017**, *5*, 64, doi:10.11114/jets.v5i4.2049.
340. Kutcher, S.; Wei, Y.; Gilberds, H.; Ubuguyu, O.; Njau, T.; Brown, A.; Sabuni, N.; Magimba, A.; Perkins, K. A School Mental Health Literacy Curriculum Resource Training Approach: Effects on Tanzanian Teachers' Mental Health Knowledge, Stigma and Help-Seeking Efficacy. *International journal of mental health systems* **2016**, *10*, doi:10.1186/S13033-016-0082-6.
341. Pallangyo, P.; Misidai, N.; Komba, M.; Mkojera, Z.; Swai, H.J.; Hemed, N.R.; Mayala, H.; Bhalia, S.; Millinga, J.; Mollel, U.W.; et al. Knowledge of Cardiovascular Risk Factors among Caretakers of Outpatients Attending a Tertiary Cardiovascular Center in Tanzania: A Cross-Sectional Survey. *BMC cardiovascular disorders* **2020**, *20*, doi:10.1186/S12872-020-01648-1.
342. Perri-Moore, S.; Routen, T.; Shao, A.F.; Rambaud-Althaus, C.; Swai, N.; Kahama-Maro, J.; D'Acremont, V.; Genton, B.; Mitchell, M. Using an eIMCI-Derived Decision Support Protocol to Improve Provider–Caretaker Communication for Treatment of Children Under 5 in Tanzania. *Global Health Communication* **2015**, *1*, 41–47, doi:10.1080/23762004.2016.1181486.
343. Stone, C.A.; Siril, H.; Nampanda, E.; Garcia, M.E.; Tito, J.; Nambiar, D.; Chalamilla, G.; Kaaya, S.F. "I Didn't Know That ..." Patient Perceptions of Print Information, Education, and Communication Related to HIV/AIDS Treatment. *Tanzania journal of health research* **2011**, *13*, doi:10.4314/THRB.V13I2.56112.
344. York, K.J.; Kabole, I.; Mrisho, M.; Berry, D.M.; Schmidt, E. Factors Affecting Community Participation in the CDTI Program in Morogoro, Tanzania. *Journal of Nursing Scholarship* **2015**, *47*, 96–104, doi:10.1111/jnu.12121.
345. Jaiteh, F.; Masunaga, Y.; Okebe, J.; D'Alessandro, U.; Balen, J.; Bradley, J.; Gryseels, C.; Ribera, J.M.; Grietens, K.P. Community Perspectives on Treating Asymptomatic Infections for Malaria Elimination in The Gambia. *Malaria journal* **2019**, *18*, doi:10.1186/S12936-019-2672-7.
346. Touray, S.; Sanyang, B.; Zandrow, G.; Touray, I. Incidence and Outcomes after Out-of-Hospital Medical Emergencies in Gambia: A Case for the Integration of Prehospital Care and Emergency Medical Services in Primary Health Care. *Prehospital and Disaster Medicine* **2018**, *33*, 650–657, doi:10.1017/S1049023X1800105X.
347. Akena, D.; Kiguba, R.; Muhwezi, W.W.; Kwesiga, B.; Kigozi, G.; Nakasujja, N.; Lukwata, H. The Effectiveness of a Psycho-Education Intervention on Mental Health Literacy in Communities Affected by the COVID-19 Pandemic-a Cluster Randomized Trial of 24 Villages in Central Uganda-a Research Protocol. *Trials* **2021**, *22*, doi:10.1186/S13063-021-05391-6.
348. Bakeera, S.K.; Wamala, S.P.; Galea, S.; State, A.; Peterson, S.; Pariyo, G.W. Community Perceptions and Factors Influencing Utilization of Health Services in Uganda. *International journal for equity in health* **2009**, *8*, doi:10.1186/1475-9276-8-25.
349. Cattamanchi, A.; Miller, C.R.; Tapley, A.; Haguma, P.; Ochom, E.; Ackerman, S.; Davis, J.L.; Katamba, A.; Handley, M.A. Health Worker Perspectives on Barriers to Delivery of Routine Tuberculosis Diagnostic Evaluation Services in Uganda: A Qualitative Study to Guide Clinic-Based Interventions. *BMC health services research* **2015**, *15*, doi:10.1186/S12913-014-0668-0.
350. Chang, A.Y.; Nabbaale, J.; Nalubwama, H.; Okello, E.; Ssinabulya, I.; Longenecker, C.T.; Webel, A.R. Motivations of Women in Uganda Living with Rheumatic Heart Disease: A Mixed Methods Study of Experiences in Stigma, Childbearing, Anticoagulation, and Contraception. *PloS one* **2018**, *13*, doi:10.1371/JOURNAL.PONE.0194030.
351. Guttersrud, Ø.; Naigaga, M.D.A.S.; Pettersen, K.S. Measuring Maternal Health Literacy in Adolescents Attending Antenatal Care in Uganda. *J Nurs Meas* **2015**, *23*, 55E – 66.
352. Jatho, A.; Mugisha, N.M.; Kafeero, J.; Holoya, G.; Okuku, F.; Niyonzima, N.; Orem, J. Capacity Building for Cancer Prevention and Early Detection in the Ugandan Primary Healthcare Facilities: Working toward Reducing the Unmet Needs of Cancer Control Services. *Cancer medicine* **2021**, *10*, 745–756, doi:10.1002/CAM4.3659.
353. Jatho, A.; Bikaitwoha, M.E.; Mugisha, N.M. Socio-Culturally Mediated Factors and Lower Level of Education Are the Main Influencers of Functional Cervical Cancer Literacy among Women in Mayuge, Eastern Uganda. *Ecancermedicalscience* **2020**, *14*, doi:10.3332/ECANCER.2020.1004.
354. Jones, S.; Norton, B. On the Limits of Sexual Health Literacy: Insights From Ugandan Schoolgirls. *Diaspora, Indigenous, and Minority Education* **2007**, *1*, 285–305, doi:10.1080/15595690701563998.
355. Kaddumukasa, M.; Smith, P.J.; Kaddumukasa, M.N.; Kajumba, M.; Almojuela, A.; Bobholz, S.; Chakraborty, P.; Sinha, D.D.; Nakasujja, N.; Kakooza-Mwesige, A.; et al. Epilepsy Beliefs and Misconceptions among Patient and Community Samples in Uganda. *Epilepsy and Behavior* **2021**, *114*, doi:10.1016/j.yebeh.2020.107300.

356. Kendrick, M.; Mutonyi, H. Meeting the Challenge of Health Literacy in Rural Uganda: The Critical Role of Women and Local Modes of Communication. *Diaspora, Indigenous, and Minority Education* **2007**, *1*, 265–283.
357. Lynch, H.M.; Green, A.S.; Nanyonga, R.C.; Gadikota-Klumpers, D.D.; Squires, A.; Schwartz, J.I.; Heller, D.J. Exploring Patient Experiences with and Attitudes towards Hypertension at a Private Hospital in Uganda: A Qualitative Study. *International journal for equity in health* **2019**, *18*, doi:10.1186/S12939-019-1109-9.
358. Miller, A.P.; Ziegel, L.; Mugamba, S.; Kyasanku, E.; Wagman, J.A.; Nkwanzi-Lubega, V.; Nakigozi, G.; Kigozi, G.; Nalugoda, F.; Kigozi, G.; et al. Not Enough Money and Too Many Thoughts: Exploring Perceptions of Mental Health in Two Ugandan Districts Through the Mental Health Literacy Framework. *Qualitative Health Research* **2021**, *31*, 967–982, doi:10.1177/1049732320986164.
359. Miller, A.N.; Napakol, A.; Kujak, M.K. Representation of Mental Illness in Leading Ugandan Daily Newspapers: A Content Analysis. *Health Communication* **2020**, *35*, 1782–1790, doi:10.1080/10410236.2019.1663469.
360. Naigaga, M.D.; Guttersrud, Ø.; Pettersen, K.S. Measuring Maternal Health Literacy in Adolescents Attending Antenatal Care in a Developing Country - the Impact of Selected Demographic Characteristics. *Journal of Clinical Nursing* **2015**, *24*, 2402–2409, doi:10.1111/jocn.12796.
361. Nalukenge, W.; Martin, F.; Seeley, J.; Kinyanda, E. Knowledge and Causal Attributions for Mental Disorders in HIV-Positive Children and Adolescents: Results from Rural and Urban Uganda. *Psychology, Health and Medicine* **2019**, *24*, 21–26, doi:10.1080/13548506.2018.1467021.
362. Nsangi, A.; Semakula, D.; Oxman, A.D.; Austvoll-Dahlgren, A.; Oxman, M.; Rosenbaum, S.; Morelli, A.; Glenton, C.; Lewin, S.; Kaseje, M.; et al. Effects of the Informed Health Choices Primary School Intervention on the Ability of Children in Uganda to Assess the Reliability of Claims about Treatment Effects, 1-Year Follow-up: A Cluster-Randomised Trial. *Trials* **2020**, *21*, doi:10.1186/S13063-019-3960-9.
363. Nwosu, C.; Spears, C.A.; Pate, C.; Gold, D.T.; Bennett, G.; Haglund, M.; Fuller, A. Influence of Caretakers' Health Literacy on Delays to Traumatic Brain Injury Care in Uganda. *Annals of global health* **2020**, *86*, 1–9, doi:10.5334/AOGH.2978.
364. Okello, E.S.; Abbo, C.; Muhwezi, W.W.; Akello, G.; Ovuga, E. *Mental Health Literacy among Secondary School Students in North and Central Uganda: A Qualitative Study*; 2014;
365. Patterson, K.; Clark, S.; Berrang-Ford, L.; Lwasa, S.; Namanya, D.; Twebaze, F.; Harper, S.L.; Ford, J.; Carcamo, C.; Llanos, A.; et al. Acute Gastrointestinal Illness in an African Indigenous Population: The Lived Experience of Uganda's Batwa. *Rural and remote health* **2020**, *20*, doi:10.22605/RRH5141.
366. Semakula, D.; Nsangi, A.; Oxman, A.D.; Oxman, M.; Austvoll-Dahlgren, A.; Rosenbaum, S.; Morelli, A.; Glenton, C.; Lewin, S.; Nyirazinyoye, L.; et al. Effects of the Informed Health Choices Podcast on the Ability of Parents of Primary School Children in Uganda to Assess the Trustworthiness of Claims about Treatment Effects: One-Year Follow up of a Randomised Trial. *Trials* **2020**, *21*, 187, doi:10.1186/s13063-020-4093-x.
367. Swahn, M.; Braunstein, S.; Kasirye, R. Demographic and Psychosocial Characteristics of Mobile Phone Ownership and Usage among Youth Living in the Slums of Kampala, Uganda. *Western Journal of Emergency Medicine* **2014**, *15*, 600–603, doi:10.5811/westjem.2014.4.20879.
368. Wandera, M.N.; Kasumba, B. "Ebinyo"-The Practice of Infant Oral Mutilation in Uganda. *Frontiers in public health* **2017**, *5*, doi:10.3389/FPUBH.2017.00167.
369. Yantzi, R.; Walle, G. van de; Lin, J. "The Disease Isn't Listening to the Drug": The Socio-Cultural Context of Antibiotic Use for Viral Respiratory Infections in Rural Uganda. *Global public health* **2019**, *14*, 750–763, doi:10.1080/17441692.2018.1542017.
370. Davies, S.; Mulwanda, J.; Gorman, D.R.; Davies, A. Testing the Health Literacy of Nurses in Zambia. *Southern African Linguistics and Applied Language Studies* **2009**, *27*, 439–452, doi:10.2989/SALALS.2009.27.4.5.1025.
371. Mwambwa-Johnson, E.Y. Mental Health Literacy Among Rural and Urban Young Adults in Zambia, Walden University, 2021.
372. Schrauben, S.J.; Wiebe, D.J. Health Literacy Assessment in Developing Countries: A Case Study in Zambia. *Health promotion international* **2017**, *32*, 475–481, doi:10.1093/heapro/dav108.
373. Sharpe, D.; Rajabi, M.; Chileshe, C.; Joseph, S.M.; Sesay, I.; Williams, J.; Sait, S. Mental Health and Wellbeing Implications of the COVID-19 Quarantine for Disabled and Disadvantaged Children and Young People: Evidence from a Cross-Cultural Study in Zambia and Sierra Leone. *BMC psychology* **2021**, *9*, doi:10.1186/S40359-021-00583-W.
374. Topp, S.M.; Sharma, A.; Chileshe, C.; Magwende, G.; Henostroza, G.; Moonga, C.N. The Health System Accountability Impact of Prison Health Committees in Zambia. *International journal for equity in health* **2018**, *17*, doi:10.1186/S12939-018-0783-3.
375. Underwood, C.; Serlemitsos, E.; Macwangi, M. Health Communication in Multilingual Contexts: A Study of Reading Preferences, Practices, and Proficiencies among Literate Adults in Zambia. *Journal of Health Communication* **2007**, *12*, 317–337, doi:10.1080/10810730701325962.
376. Smythe, T.; Mabhena, T.; Murahwi, S.; Kujinga, T.; Kuper, H.; Rusakaniko, S. A Path toward Disability-Inclusive Health in Zimbabwe Part 1: A Qualitative Study on Access to Healthcare. *African journal of disability* **2022**, *11*, doi:10.4102/AJOD.V11I0.990.
377. Terry, P.E.; Masvaure, T.B.; Gavin, L. HIV/AIDS Health Literacy in Zimbabwe Focus Group Findings from University Students. *Methods Inf Med.* **2005**, *44*, 288–292.
