# Supplementary material for: Health Literacy in Africa—A Scoping Review of Scientific Publications
Source: Int J Environ Res Public Health. 2024 Oct 31;21(11):1456. doi: 10.3390/ijerph21111456 (PMC11594271; doi:10.3390/ijerph21111456)
Supplement: Supplementary file 1 [file ijerph-21-01456-s001.zip › AfricanHL_Supplement file S3.pdf]

Supplement File S3: Trends of health literacy research in the various African countries

Country	Number studies(1)	Year	Health literacy focus
African but not individual country specific records			
Africa	16	2008	HL, CD (Covid-19), NCD (cancer), oral health, maternal health, reproductive health, patient & palliative care
Sub-Saharan Africa	10	2015	Mental health, CD (HIV/Aids), NCD (stroke), HL, men's health, maternal health, disease literacy
**Northern Africa**			
Algeria	0/1	2019	Acceptance of app (diabetes)
Egypt	7/2	2015	Health literacy, NCD (cancer), antibiotic resistance
Libya	0/0	n.d.	n.d.
Morocco	2/0	2018	CD (tuberculosis), asthma medication adherence
Sudan	0/1	2021	CD (Covid-19)
Tunisia	0/0	n.d.	n.d.
**Eastern Africa**			
Burundi	0/1	2022	mental health
Comoros	0/0	n.d.	n.d.
Djibouti	0/0	n.d.	n.d.
Eritrea	0/0	n.d.	n.d.
Ethiopia	25/3	2012	CD (Covid-19, Podoconiosis, tuberculosis), NCD (diabetes, cancer), maternal health, child health, mental health, ehealth, health literacy (including functional HL, communicative HL) health service provision, medication HL
Kenya	11/2	2010	NCD (cancer), mental health, maternal and child health, sexual and reproductive health, environmental HL, HL
Madagascar	2/0	2015	Nutrition, maternal HL
Malawi	10/4	2015	CD (Covid-19, HIV), NCD (diabetes, cancer), maternal health, child health, Mental health, /mental health
Mauritius	0/0	n.d.	n.d.
Mozambique	3/0	2014	HIV, nutrition
Réunion	0/0	n.d.	n.d.
Rwanda	6/1	2016	CD (Malaria), NCD (diabetes), HL, diseases literacy, mental health, patient-provider communication/HL
Seychelles	0/0	n.d.	n.d.
Somalia	1/0	2021	Language in health care
South Sudan	0/0	n.d.	n.d.
Uganda	23/4	2007	CD (tuberculosis), NCD (heart disease, cancer, gastrointestinal, hypertension,) health care utilization,

Country	Number studies(1)	Year	Health literacy focus
			HL, medication, maternal health, mental health, sexual HL, infant oral mutilation
Tanzania	11/3	2009	HL, mental health, NDC (cardiovascular disease) empowerment, Mental health/antenatal HL
Zambia	5/7	2007	HL, mental health, prison committee/HL, antenatal HL
Zimbabwe	2/2	2005	Disability, HL/HIV
**Central Africa**			
Angola	1/0	2022	HL questionnaire
Cameroon	1/2	2021	NCD (cancer)
Central African Republic	0/0	n.d.	n.d.
Chad	0/0	n.d.	n.d.
Congo	0/1	n.d.	n.d.
Democratic Republic of the Congo	1/1	2018	Heart failure, health literacy
Equatorial Guinea	0/0	n.d.	n.d.
Gabon	0/0	n.d.	n.d.
Sao Tome and Principe	0/0	n.d.	n.d.
**Southern Africa**			
Botswana	1/2	2016	HIV, life-style (alcohol)
Eswatini	1/0	2022	Mental health
Lesotho	2/1	2016	Hypertension, HL/HL
Namibia	1/3	2020	Health literacy/HL
South Africa	74/11	2008	CD (HIV, Covid-19, tuberculosis, schistosomiasis); NCD (cancer, stroke, diabetes, heart disease, chronic), HL, mental health, nutrition, occupational H, oral H, organ donation, reproductive health, social media, drug prevention, health care
**Western Africa**			
Benin	1/1	2015	Oral / health care service
Burkina Faso	3/1	2013	Covid-19, reproductive health, oral health, Covid-19
Cabo Verde	1/1	2015	Sexual and reproductive health, cardiovascular diseases / Covid-19
Côte d'Ivoire	1/1	2020	HIV&Aids / HL
The Gambia	2/0	2019	Malaria
Ghana	26/4	2014	Mental health, maternal/antenatal health, CD (malaria, cholera, HIV), NDC (breast cancer), foodborne disease, HL, oral health, sexual and reproductive health, social participation/HL
Guinea	1/1	2006	Reproductive health/health literacy
Guinea-Bissau	1/1	2016	HIV / Covid-19

Country	Number studies(1)	Year	Health literacy focus
Liberia	1/1	2021	Mental health
Mali	0/0	n.d.	n.d.
Mauritania	0/0	n.d.	n.d.
Niger	1/1	2016	CD (malaria)
Nigeria	45/4	2010	HL, maternal and child health, mental health, CD (tuberculosis, vaccine, Covid-19), NCD (cancer, diabetes, stroke), sexual and reproductive health), occupational HL, oral health, genomic literacy, eye care, environmental HL, ehealth
Saint Helena	0/0	n.d.	n.d.
Senegal	3/0	2016	Oral health, health information, family planning
Sierra Leone	1/3	2022	Treatment adherence/HL
Togo	0/1	2020	n.d./HL

Note: Classification based on the UN-Classification URL: https://unstats.un.org/unsd/methodology/m49/

(1) First number indicates records that include only that specific country, second number indicates whether country is included in a multi-country study
